# Probabilistic reporting and algorithms in forensic science: Stakeholder perspectives within the American criminal justice system

**DOI:** 10.1016/j.fsisyn.2022.100220

**Published:** 2022-02-12

**Authors:** H. Swofford, C. Champod

**Affiliations:** School of Criminal Justice, Forensic Science Institute, University of Lausanne, Switzerland

**Keywords:** Forensic science, Pattern evidence, Probabilities, Statistics, Algorithms

## Abstract

In recent years, there have been efforts to promote probabilistic reporting and the use of computational algorithms across several forensic science disciplines. Reactions to these efforts have been mixed—some stakeholders argue they promote greater scientific rigor whereas others argue that the opacity of algorithmic tools makes it challenging to meaningfully scrutinize the evidence presented against a defendant resulting from these systems. Consequently, the forensic community has been left with no clear path to navigate these concerns as each proposed approach has countervailing benefits and risks. To explore these issues further and provide a foundation for a path forward, this study draws on semi-structured interviews with fifteen participants to elicit the perspectives of key criminal justice stakeholders, including laboratory managers, prosecutors, defense attorneys, judges, and other academic scholars, on issues related to interpretation and reporting practices and the use of computational algorithms in forensic science within the American legal system.

## 1 Introduction

Forensic science has long been considered a cornerstone for advancing investigations and establishing facts in question to support criminal and civil litigation. Under the powerful aura of science, interpretations and conclusions made by forensic experts are often presented as tantamount to fact—the silent witness—that courts can rely on in their pursuit of justice. For decades on end, forensic evidence was broadly considered infallible and rarely questioned. In February 2009, however, that all changed with the release of the National Research Council's (NRC) report on the needs of the forensic science community, highlighting that “[t]he law's greatest dilemma in its heavy reliance on forensic evidence, however, concerns the question of whether—and to what extent—there is *science* in any given forensic science discipline” [1]. Following their analysis of several forensic science disciplines, the NRC noted: “The simple reality is that the interpretation of forensic evidence is not always based on scientific studies to determine its validity. This is a serious problem. Although research has been done in some disciplines, there is a notable dearth of peer-reviewed, published studies establishing the scientific bases and validity of many forensic methods.” The NRC goes on to assert “no forensic method other than nuclear DNA analysis has been rigorously shown to have the capacity to consistently and with a high degree of certainty support conclusions about ‘individualization’ (more commonly known as ‘matching’ of an unknown item of evidence to a specific known source)” [1]. The NRC report, although positive in the sense that it raised awareness of the need for greater resources, offered damning critiques to a body of evidence that was often presented, and perceived, as essentially infallible.

In the years that followed, these types of critiques have become commonplace—particularly as it relates to concerns over the high reliance on subjectivity and lack of statistical foundations supporting the interpretation of results, as well as concerns over the expression of conclusions asserting a level of certainty that implies infallibility. For example, in 2012 a committee supported by the National Institute of Standards and Technology (NIST) and the National Institute of Justice (NIJ) issued several recommendations specific to improving friction ridge examinations, claiming: “Because empirical evidence and statistical reasoning do not support a source attribution to the exclusion of all other individuals in the world, latent print examiners should not report or testify, directly or by implication, to a source attribution to the exclusion of all others in the world” [2]. This was followed by another landmark report offered by the President's Council of Advisors on Science and Technology (PCAST) in 2016, asserting: “Statements claiming or implying greater certainty than can be demonstrated by empirical evidence are scientifically invalid. Forensic examiners should therefore report their findings with clarity and restraint, explaining in each case that the fact that two samples satisfy a method's criteria for a proposed match does not necessarily imply that the samples come from a common source. … [C]ourts should never permit scientifically indefensible claims” [3]. Finally, in 2017, the friction ridge community was faced with, yet again, another critique, but this time coming from the American Association for the Advancement of Science (AAAS)—the world's largest scientific society. Following a scientific gap assessment of the research supporting the existing methods, the AAAS committee stated: “Examiners should be careful not to make statements in reports or testimony that exaggerate the certainty of their conclusions …. [T]hey should avoid statements that claim or imply that the pool of possible sources is limited to a single person. Terms like ‘match,’ ‘identification,’ ‘individualization,’ and their synonyms, imply more than the science can sustain” [4].

In light of these concerns, increasing calls have been made for the introduction of probabilistic reasoning and the use of validated statistical methods into forensic practice—particularly in the pattern evidence disciplines—to formally recognize and articulate the uncertainties inherent in forensic interpretation and to reduce the heavy reliance on subjective judgment [[1], [2], [3], [4]]. Over the years, a number of reputable efforts have been made by researchers to explore the optimal approach for expressing forensic conclusions to maximize lay fact-finders’ interpretation (e.g., see Ref. [5]) and, in the friction ridge discipline in particular, to introduce probabilistic models—often through computational algorithms^1^—to provide statistical foundations to the analysis and evaluation of evidence [[6], [7], [8], [9], [10], [11], [12], [13], [14], [15], [16], [17], [18], [19], [20], [21], [22], [23], [24], [25], [26], [27], [28], [29], [30], [31], [32], [33]]. Although probabilistic reporting is often presented as a scientifically superior approach to expressing forensic results compared to traditional categorical assertions, it is often more difficult for lay fact-finders to interpret [5]. Likewise, although algorithmic tools generally possess remarkable potential to provide advanced scientific capabilities and promote more objective foundations to the evaluation of forensic evidence, they often do so at the cost of transparency and explainability [[34], [35], [36], [37], [38], [39], [40]], which have been argued to stifle meaningful scrutiny and accountability of the evidence resulting from these tools thereby infringing on criminal defendants’ Constitutional rights (e.g., see Refs. [34,35,37,38]). Consequently, the forensic community has been left with no clear path forward on how to navigate these mounting concerns as each proposed solution seemingly has countervailing benefits and risks. In recent work, we began to explore some of these issues in greater detail based on perspectives that have been raised in the literature thus far and provided some initial recommendations relating to the operational implementation of computational algorithms [41]. This current study further explores those issues with greater breadth and depth, but it is only a start to what we consider to be a much needed, and much more extensive, discussion on these issues so that the forensic and legal communities can begin addressing these challenges that are no longer over the horizon.

As the forensic community continues to grapple with these issues, widespread reform efforts have been understandably slow. However, a few notable steps have been taken in an effort to heed the recommendations from various scientific committees. In 2015, the United States Army Criminal Investigation Laboratory (USACIL), the primary forensic laboratory supporting the criminal investigative mission of the Department of Defense, announced a policy change to abandon the term “identification” and report their findings in a probabilistic framework (albeit in the absence of a computational algorithm) [42]. In 2017, USACIL went a step further and announced the implementation of a statistical software application, *FRStat*, to provide statistical support to fingerprint associations [33,43]. This has been considered by some as a step in the right direction to reduce variability and improve overall consistency between analysts (e.g., Refs. [44,45]). Then, in 2018, the Organization of Scientific Area Committees (OSAC) for Forensic Science, Friction Ridge Subcommittee (OSAC FRS), which is responsible for the promulgation of standards and best practices related to the forensic examination of friction ridge skin impression evidence throughout the United States, released the proposed standard for Friction Ridge Examination Conclusions [46], taking an additional step toward promoting probabilistic expressions on a national level. While the proposed standard maintains the term “identification,” which has traditionally been used to express categorical conclusions, it was redefined in a probabilistic framework as a qualitative (non-numeric) expression of a likelihood ratio. In addition to the revised definition, the OSAC FRS stated that “an examiner shall not assert that a source identification is the conclusion that two impressions were made by the same source or imply an individualization to the exclusion of all other sources” [46], a claim which has been a common hallmark of categorical statements.

Despite these efforts, probabilistic reporting and statistical interventions continue to be a contentious topic within the forensic science community, with some forensic friction ridge practitioners welcoming it with open arms as a more “scientifically defensible” approach while others express passive skepticism or outright opposition [47]. Although significant resistance remains across the friction ridge discipline and probabilistic reporting remains rare, approximately one-third of survey participants who currently report categorically seem to be receptive to the idea of reporting probabilistically, but remain hesitant to adopt for one reason or another [47]. Practitioners’ perspectives have been instrumental in highlighting a number of social scientific issues that are believed to have contributed to this hesitancy (i.e., educational, philosophical, psychological and complex judicial implications and longstanding cultural and institutional norms) thereby allowing us to consider strategies to address their concerns [47]. While forensic practitioners will ultimately be responsible for implementing the proposed solutions, it would be incomplete to focus *solely* on perspectives of forensic practitioners.

To fully understand the issues and more effectively facilitate improvements to traditional practices, we must also account for the perspectives of *all* stakeholders within the criminal justice system—not just forensic practitioners. Recognizing that prior work has captured the broad perspectives of friction ridge practitioners (i.e., [47]), this study aims to explore the individual perspectives of other key criminal justice stakeholders based on their different roles in the criminal justice system—including forensic laboratory managers, prosecuting attorneys, defense attorneys, judges, and other academic scientists and scholars—to provide a better understanding of their distinct values and interests on issues related to: (i) interpretation and reporting practices (with or without algorithmic tools) and (ii) the implications of the use of algorithms in legal settings as a means of calculating the probabilistic values assigned to the evidence.

## 2 Materials & methods

This study was conducted as one-on-one semi-structured interviews between the first author and each individual stakeholder using the video-based virtual meeting platform Zoom®. Although the qualitative nature of this approach prohibits broad generalizations and quantitative representations, it does allow us to explore these various perspectives in greater depth and with more clarity than if it were presented as a structured survey. Participants were solicited by invitation (see Appendix I) based on having been actively engaged in issues concerning forensic science policies, procedures, and practices. These participants have occupied prominent roles in their disciplines (e.g., senior and executive level positions in their organizations and professional societies), have been selected to serve on boards and committees steering policy and practice recommendations (e.g., National Commission on Forensic Science, Organization of Scientific Area Committees for Forensic Science), have made academic contributions to forensic science practices through professional publications and presentation, or have influenced the practices of others across the broader community, either directly through supervision or indirectly through training and continuing education activities. Overall, a total of twenty-two individuals were invited to participate in the study and seven individuals declined to participate (four individuals did not respond to the invitation [one forensic laboratory manager, one prosecuting attorney, and two judges], two individuals cited competing priorities and commitments to participate within the intended timeframe [one forensic laboratory manager and one judge], and one individual expressed support for the study but felt unable to answer the questions related to the use of algorithms [academic scholar]). Invitations were extended to potential participants until three individuals agreed to participate for each stakeholder group (forensic laboratory managers, prosecuting attorneys, defense attorneys, judges, and other academic scientists and scholars) resulting in a total of fifteen participants. Specific details related to the backgrounds and experiences for those individuals who agreed to participate are provided in the Results section for each stakeholder group.

Interviews were conducted between September and November 2021 and were scheduled based on participants' availability, thereby enabling an arbitrary sequence of participants (i.e., stakeholder participants were arbitrarily spread throughout and not interviewed in any particular sequence). Participants’ personal identities are not disclosed or publicly attributed to any specific statements. Each participant was assigned a unique identifier within their stakeholder group to distinguish among responses from individual participants. Prior to the study commencing and as part of the initial invitation, participants were provided an Information and Informed Consent sheet that summarized the structure of the study (see Appendix II), a summary of the purpose and background of the study that included specific terms and definitions related to the interview questionnaire (see Appendix III), and a general outline along with a set of structured questions to guide the interview (see Appendix IV).

Participants were first presented with a series of questions pertaining to their demographics (occupation, experience, education, and exposure to algorithms). Participants were then asked a series of structured questions addressing various topics (described below) pertaining to their perspectives related to interpretation and reporting and the use of computational algorithms for court purposes. Although most participants offered responses to all of the structured questions, in a few instances some questions were omitted during the interviews due to time constraints; thus, not every participant provided a separate response to each individual question. Throughout the interview, unstructured questions were raised *ad hoc* to explore participants’ responses in further detail and to elicit their perspectives related to responses provided by other participants interviewed thus far.

Questions related to the broader issue of interpretation and reporting sought to elicit participants’ perspectives around four broad topics:

The first topic focuses on the validity, appropriateness, benefits, and limitations/risks of categorical reporting compared to probabilistic reporting methods. These concepts have become central to the broader discourse concerning how forensic science testimony should be delivered and have been at the forefront of the friction ridge discipline for over a decade (e.g., see Refs. [[1], [2], [3], [4],^47^])—often resulting in heated debates within the forensic practitioner community [47].

The second topic points to salient concerns raised by friction ridge practitioners as it relates to the use of probabilistic reporting. In a recent study surveying various reasons for practitioners’ opposition to probabilistic reporting, the most common concerns cited by friction ridge practitioners related to how defense attorneys might (mis)use probabilistic reporting to “create reasonable doubt” and whether jurors would understand the conclusion being conveyed [47]. The findings from this survey raise other questions concerning the role/duties of experts as it relates to the limits of their testimony and whether, and to what extent, such factors ought to be taken into account by forensic practitioners when considering the most appropriate means of expressing forensic conclusions. In other words, should forensic practitioners focus on not only the validity and appropriateness of such claims, but also how those conclusions might factor into litigation strategies for one or both sides or be perceived by fact-finders? All these concerns are relevant, but how they should be addressed and by whom remains an open question.

The third topic focuses on whether it is necessary for forensic practitioners to disclose underpinnings or statistical data to support their testimony. This topic was motivated primarily by the PCAST argument that “[s]tatements claiming or implying greater certainty than can be demonstrated by empirical evidence are scientifically invalid” and “[n]othing—not personal experience nor professional practices—can substitute for adequate empirical demonstration of accuracy” [3]. Such claims by the PCAST suggest all forensic testimony must be accompanied by empirical foundations underpinning such claims. It also raises the question whether statistical data is meant to be the means for providing the empirical foundations. This is impactful to friction ridge practitioners, as traditional practices encourage experts to base their conclusions on “training and experience” and to couch their conclusions as an expression of their opinion rather than basing them on statistical measurements. It raises the question as to whether other stakeholder groups share the perspective suggested by the PCAST and how this might be more explicitly required in the longer term. Indeed, proposed amendments to Federal Rule 702 have been made to address “the problem of overstating results” and “emphasize that the court must focus on the expert's opinion, and must find that the opinion actually proceeds from a reliable application of the methodology” when considering the admissibility of expert testimony [48]. The full implications of such a proposal, however, remains unclear.

The fourth topic focuses on what participants view as the most significant challenges facing the pattern evidence disciplines relating to examination and reporting. This topic is intended to highlight how the pattern evidence disciplines might need to consider adapting in light of the various perspectives raised by the different stakeholders on this broader issue of interpretation and reporting.

Questions related to the broader issue of the use of computational algorithms for court purposes sought to elicit participants’ perspectives around five broad topics:

The first topic focuses on the role computational algorithms should play in forensic science for court purposes along with the benefits and limitations/risks of such applications. These issues have become central to the broader discussion of responsible applications AI in society. As computational algorithms have advanced and automated decision systems have become more accessible, researchers, advocates, and policy makers are debating when and where these systems are appropriate—including particularly sensitive domains such as criminal justice [49]. Questions have been raised on how to fully assess the short and long-term impacts of these systems and the appropriateness of their applications given many operate as “black-boxes” [49]. These are broad questions for which stakeholders often disagree. In the context of forensic science, perspectives on these issues have yet to be fully explored.

The second topic focuses on the concept of “trust” with computational algorithms and what artifacts are needed for stakeholders to be comfortable with the use of an algorithmic tool. For example, is source code a necessary requisite for an algorithm to be trusted? In recent years, particularly in the context of probabilistic genotyping algorithms, courts have grappled with legal issues surrounding whether they can or should compel disclosure of source code due to countervailing positions related to trade secret violations. These issues have become a growing source of controversy affecting whether algorithms should be used in forensic science more broadly [34].

The third topic expands on the concept of “trust” and points specifically to computational algorithms based on AI/ML methods. Recognizing that source code has often been the focus of legal debates as it relates to the admissibility of algorithms based on human interpretable rules or processes, what about algorithms that are based on non-human interpretable processes, such as those developed through AI/ML methods? Computational algorithms based on AI/ML are often “black boxes” even to their developers, irrespective of the availability of source code. Given this additional layer of opacity, is it appropriate to use computational algorithms based on AI/ML methods in forensic science for court purposes? If so, under what circumstances should they be used?

The fourth topic addresses the issue of regulating computational algorithms. This issue was motivated by recently proposed legislation, the Justice in Forensic Algorithms Act of 2019, to “prohibit the use of trade secrets privileges to prevent defense access to evidence in criminal proceedings, provide for the establishment of Computational Forensic Algorithm Testing Standards and a Computational Forensic Algorithm Testing Program, and for other purposes” [50]. Among other implications of this proposed legislation, it would prohibit the use of computational forensic algorithms unless they have been tested by the Computational Forensic Algorithm Testing Program and the developers of the algorithmic tools agree to waive any and all legal claims related to the defense analyzing or testing the computational forensic software [50]. Although this proposed legislation remains early stage, it raises the question of whether computational algorithms should be regulated, and, if so, by whom and how. Is the adversarial system sufficiently positioned to regulate computational algorithms as they currently do with the admissibility of expert testimony? Should specific algorithmic tools be “approved” by an external authority prior to authorizing their use? If so, should it be administered by a government entity (federal, state or local) or other non-government institution? The issue of regulation raises several other complex questions and takes on several different dimensions that have yet to be fully explored.

The fifth topic focuses on what participants view as the most significant challenges facing the pattern evidence disciplines relating to the operational use of computational algorithms in forensic science for court purposes. This topic is intended to highlight how the pattern evidence disciplines might need to consider adapting in light of the various perspectives raised by the different stakeholders on this broader issue of the use of computational algorithms.

Interviews were recorded (audio and video) using the Zoom® virtual meeting platform. The full recording was transcribed using the Descript® transcription platform [51] using a two-stage approach. First, transcriptions were initially performed using the Descript® commercial machine transcription software to automatically detect speakers, transcribe the audio, and align transcribed text to the audio and video [51]. Second, using the manual transcription editing features with the text, audio, and video, aligned within the Descript® platform [51], the machine transcription was reviewed by the first author to confirm accurate transcription and manually correct any errors. The transcribed interview was then exported to a Microsoft Word® document. Overall, this resulted in over 20 h of recorded interviews and over three hundred pages of written transcripts. The transcribed text from the interviews were then qualitatively analyzed by categorizing participants' responses based on the specific topics being explored (e.g., within the broader issue of “interpretation and reporting,” participants' responses that were related to the validity, appropriateness, benefits, and limitations/risks of categorical reporting were categorized separately from the other topics described earlier). Then, within the categorized responses for each participant, specific excerpts were identified that succinctly represented each participant's viewpoint. This approach allows us to capture specific comments made by individual participants *in their own words*, summarize participants' perspectives for each topic explored, and compare those perspectives both within and between the different stakeholder groups.

The perspectives of each stakeholder group are presented separately. This enables us to understand the source(s) of the different perspectives and compare those perspectives across the different stakeholder groups, which is a key objective of this study. Although all stakeholders share a common goal for an effective administration of justice, they each serve very different roles and responsibilities, and therefore may view various issues differently based on those roles. For example, forensic laboratory managers are responsible for ensuring they have the personnel, resources, and equipment to examine cases effectively and efficiently to keep pace with the growing demands and are therefore often focused on ways of increasing capacity while maintaining acceptable quality standards. Prosecuting attorneys, as legal representatives of the government, are responsible for convincing a court that a particular individual is guilty of committing the crimes that they have been charged with and are therefore often focused on presenting their arguments in a manner that is comprehensible to lay fact-finders. Defense attorneys, as legal representatives of the defendant, are responsible for defending their client's interests and rights and are therefore often focused on confronting and challenging the evidence presented against them to ensure it meets the appropriate legal standards. Judges are responsible for overseeing the legal process and are therefore often focused on ensuring that applicable rules, regulations, and laws are followed by all parties and that the integrity of the process is upheld. Finally, other scientific and academic scholars are responsible for researching complex issues and making recommendations for improving policy, procedure, or practice, and therefore are often focused on considering issues in terms of scientific or legal ideals. Understanding the different perspectives from each stakeholder group and how their interests may differ as they relate to fulfilling their specific roles and responsibilities within the criminal justice system is important for us to lay the foundation and begin to navigate a path forward on these issues that is responsive to the needs of all stakeholder groups.

In order to provide such an analysis and synthesis of these various stakeholder perspectives, we have organized the information into two distinct sections. In the Results section, we present a summary of each participant's background and experiences and responses to questions addressing key topics related to the broader issues of “interpretation and reporting practices” and “use of algorithms” within each stakeholder group. Organizing the Results of the interviews in this manner allows us to compare the extent to which perspectives from individual participants are consistent with others *within* the same stakeholder group. In the Discussion section, we characterize the collective perspective representing each stakeholder group by topic and compare those perspectives across the different groups. Organizing the Discussion in this manner allows us to consider the extent to which perspectives may vary *between* different stakeholder groups and begin to understand the sources of those differences and lay a foundation for us to explore why those differences might exist. Throughout the Results section, we provide short specific quotes from individual participants to illustrate certain views or discussion points. While these quotes are intended to be illustrative, we recognize that some readers might desire to consider participants' statements in greater context of their responses from the interviews. Although full transcripts cannot be released to protect the anonymity of participants, in Appendices V and VI we provide more elaborate quotes from participants related to each topic discussed in the interview. In the Discussion section, we provide a fewer set of more elaborate quotes from participants, primarily from responses to *ad hoc* questions presented to participants throughout the interviews to illustrate other interesting points.

## 3 Results

### 3.1 Laboratory managers

#### 3.1.1 Background & experience

Three laboratory managers participated in the study—all male. All three laboratory managers are actively working in large metropolitan jurisdictions in the United States and have between 20 and 38 years of experience in forensic science. One participant's experience is dominated by trace evidence, including physical match comparisons, shoe print, tire track, textile, hair comparisons, and fiber comparisons as well as forensic serology and DNA (LM#1). The other two participants experiences were dominated by toxicology (LM#2) and analytical chemistry (LM#3). All three participants, however, currently serve as the director for their respective laboratory system, overseeing a wide range of forensic disciplines, including DNA, drug chemistry, toxicology, fingerprints, firearms, and crime scene, among others. Participants' experiences working with algorithms are varied, and include analytical instrumentation (e.g., GCMS, LCMS, etc.), breathalyzers for breath alcohol quantitation, database searching (e.g., AFIS), imaging technologies (e.g., 3D imaging for firearms), and DNA mixture interpretation (e.g., probabilistic genotyping software). One participant (LM#3) has experience developing computer software and teaches computer science (among other courses, such as physics and chemistry) at the local college. All three participants are actively engaged in national and international professional bodies and have been vocal representatives of the needs of forensic laboratories throughout the United States.

#### 3.1.2 Interpretation & reporting practices

All three laboratory managers expressed the perspective that categorical reporting in pattern evidence disciplines using terms such as “Identification” or “Individualization” have the potential to mask the uncertainty and limitations associated with the conclusion. All of the participants acknowledged that the forensic science community has historically made claims in various disciplines that were overly generalized and implied greater certainty than can be supported by the empirical evidence. However, as long as the examiners caveated the claims as being their opinion, the participants were less concerned. For example:

*Absolutes and conclusions, I think, are probably inappropriate. I, however, do not have a problem with experts giving their opinion. I think we have very good experts. I think expertise matters. I think exposure to casework matters. I do agree with a lot of the defense experts and the academics that we need a reasonably good way to express uncertainty (LM#3).*

Participants suggested that probabilistic reporting, in theory, is superior to categorical reporting because it explicitly acknowledges the uncertainty in the conclusion; however, all three participants suggested probabilistic reporting in practice had its own pitfalls. Participants were concerned that probabilistic statements would be confusing or incorrectly interpreted by lay fact-finders or would be relied upon too heavily by fact-finders assuming the numerical references were based on empirical measurements. One participant made it clear that probabilistic statements with numbers should not be used unless it was clearly based on some empirical data source (LM#3). For example:

*I like [numbers] because it provides [context]. On the other hand, even numbers have their limitations. … How do you throw somebody just a number and expect them to understand it? … It's still not standalone (LM#1).*

*From a philosophical standpoint, I think it is more appropriate. What I see though, is a hell of a lot of confusion on the part of the lay person and lawyers and juries (LM#2).*

*I have no problem with subjective interpretations [such as] “in my experiences,” [or] “is very likely,” just as a subjective conclusion, but if you're going to put a number on it, I think you need to have some basis [of] where you're pulling the number from (LM#3).*

Overall, participants generally considered the benefits of categorical reporting as its simplicity and ease for fact-finders to base their decision and it provides a more holistic assessment of the examination. However, categorical reporting is “fuzzier” and can mask the uncertainty associated with the conclusion. Participants generally considered probabilistic reporting as favorable in principle. However, noting the confusion that often accompanies probabilistic references, participants were hesitant to suggest probabilistic reporting was superior in practice. Ultimately, all participants suggested applying both approaches as part of examiners’ explanation of the evidence.

When responding to concerns raised by practitioners as it relates to probabilistic reporting, participants agreed with practitioners, expressing the view that probabilistic reporting would be confusing to lay fact-finders. However, participants did not consider this as a reason not to adopt probabilistic reporting. Two participants suggested the challenges would not be insurmountable (LM#1 and LM#2). The other participant was more cautious, suggesting the optimal approach moving forward is to adopt probabilistic reporting as supplemental to traditional categorical reporting. For example:

*Watching what I've seen happened with biology, yes, it will be confusing. Is it irrevocably confusing? No. I think everybody in the system can learn how to deal with it and how to explain it. … The practitioners are confused by it right now. But that is (1) not a reason to not go there, and (2) not an indelible absolute. The confusion will subside. The confusion will abate and people will get better about explaining it (LM#2).*

*I think the type of testimony that we're currently giving plus this is the best model for the future (LM#3).*

Participants were also sympathetic to practitioners’ expressing concerns that defense attorneys would use probabilistic reporting to create “reasonable doubt;” however, none of the participants expressed the view that it should be a reason not to consider probabilistic reporting. Rather, it represents an additional barrier that will need to be addressed by proponents of probabilistic reporting. Two of the participants considered this reaction from practitioners as reinforcement for their perspective that probabilistic reporting should not be use alone—it should always be combined with an expert opinion providing an overall conclusion (LM#1 and LM#3). The other participant expressed the view that it should not be a concern from the standpoint of being rational and neutral to the issues, but at the same time recognized the human side of practitioners and suggests that it is impractical for people to be completely divorced from the emotional aspects that motivate them to be forensic scientists to start with (LM#2).

*The last thing I want is to put something out there that can be misused. … That's why you should have the opinion that we believe that this has a likelihood of association, then you throw in the number but you give the whole package as opposed to just reporting a number that potentially could be misinterpreted (LM#1).*

*I think there is a huge grade of the concerns that all come back to the fear of the uncertainty … their fear is if we change this, I don't know what's going to happen on the other side of it (LM#2).*

When responding to questions raised about the role and duties of experts and the limits of their testimony, participants expressed the view that it is incumbent upon experts to convey those limitations to ensure the results are properly interpreted, and the conclusions are not overstated or understated. One participant pointed to consensus-based guidelines to drive how the results should be framed in order to ensure greater standardization across the field (LM#1). The other two participants recognized the challenges associated with conveying the limitations, suggesting there is not a straightforward solution (LM#2 and LM#3). One participant claimed the limitations should be explicit on the report so that stakeholders did not have to pull it out during testimony, although acknowledged this is a practice they have not yet implemented and are still working through how to accomplish it (LM#2). The other participant expressed frustration that courts have made it challenging to convey limitations unless they are directly asked, but even then, the participant recognized the difficulty of conveying them (LM#3). For example:

*I think it is an inherent obligation on the part of the expert to convey those limitations and do the best they can trying to explain the inherent uncertainty there. … [However,] this is not saying that we have effectively managed to accomplish this, we haven't (LM#2).*

*I think all of us have an ethical obligation to understand the limitations of what we're saying …. [However,] most of the time the court hearings won't allow us [to express those limitations] unless they directly ask us …. So, articulating that uncertainty is something we're not perfect [doing] yet. But, it's also one of the reasons why we don't say to the exclusion of all others [for example] (LM#3).*

When asked about whether participants find it acceptable for experts to express their opinion in court without disclosing the underpinnings or statistical data to support those opinions, all three participants strongly advised to do so; however, they also recognized it does not always come out in practice and, in some situations, suggested it may not be absolutely necessary. One participant expressed frustration that despite the laboratory's best efforts to convey those details, the legal system makes it challenging for the experts to do so during testimony (LM#2). Another participant echoed similar challenges but seemed to be more resigned to the realities of the court room environment (LM#3). For example:

*I would strongly encourage they do it because I feel it makes their opinion better, stronger (LM#1).*

*This is one of the things that I'm finding myself getting a little bit more worked up about these days, of this issue of it was the laboratory that didn't express the extent and limitations of the testing. No, the lab is willing to do that, the lab wants to do that, all the rest of the system cut it off at the knees (LM#2).*

Finally, when asked what participants would describe as the greatest challenges facing the pattern and impression evidence disciplines as it relates to examination and reporting methods, participants pointed to both cultural and resource challenges, the greatest factor being limited resources. One participant lamented that many of these scientific issues that have been at the forefront of debates seem to be trivial compared to the greater challenges of effectively managing the caseload and data management (LM#2). The other two participants referenced cultural and educational challenges (LM#1 and LM#3) as well as the inability for crime laboratories to actively engage in research given their limited resources and pressures to stay abreast of casework (LM#3). For example:

*There is still a little bit of resistance that you're taking away the expertise [the experts] already have and supplanting it with something else. That, to me, I think is completely false if you agree to integrate them both together. … The other biggest reason is that [for] crime labs, it's not our mission to do research, unfortunately. I love research and it's wonderful, but we are under so much pressure to get casework done. We just don't have the time, energy or money to do it. It's unfortunate because we're really the best place to do it, but we just don't have the money to do it (LM#3).*

#### 3.1.3 Use of algorithms

Laboratory managers offered generally consistent perspectives as it relates to the use of algorithms in court and the benefits and limitations of them. All three participants expressed favorable viewpoints of using algorithms; however, participants were clear that the algorithms should be used to supplement the judgments of examiners and not to replace them. Participants recognized the value algorithms can provide by promoting greater objectivity and consistency in the results. One participant expanded on the utility of the algorithms to be a “force multiplier” to “build capacity” to help offset the limited analysts available and keep pace with caseload and throughput demands (LM#2). However, all three participants cautioned the urge to rely too heavily on the algorithms and supplant the expert, or to blindly rely on them without fully vetting them. All three participants viewed expert judgment, while subjective, as a valued asset that can account for factors that the algorithm cannot and to help interpret and convey the output of the algorithm to judicial stakeholders. For example:

*I think that's an excellent thing to assist in better understanding why you came up with this opinion. But the danger is that people then rely too much on the number (LM#1).*

*I think the greatest benefit on the algorithms is the relative consistency of the result case over case. … [However,] I think the biggest risk is becoming overly reliant and we just exchange the categorical certain answer from the spectacle nerd for now, an infallible algorithm (LM#2).*

When asked about concerns over how algorithms can be trusted for use in court, including issues concerning the disclosure of source code, participants largely pointed to validation. Two of the participants expressed views that source-code was unnecessary and requests for disclosure were legal tactics versus genuine efforts to evaluate the algorithm (LM#1 and LM#2); however, participants were willing to support disclosure if requested and all three participants stated they would factor source code disclosure as an element when selecting a commercial vendor. One participant took it a step further and suggested algorithms should include internal controls on every single application to help establish trust rather than simply rely on an initial validation prior to casework applications (LM#2). The third participant offered a slightly different view on these issues than the other two, expressing a stronger emphasis on disclosure. This participant, (LM#3), expressed the viewpoint that understanding the internal workings of the algorithm was key for establishing trust, and source code disclosure was a way to accomplish this. This participant pointed out that validations have limitations and, while informative and important, were not a complete substitute for understanding the innerworkings of the algorithm itself, which could be obtained through public disclosure and open explanations of the conceptual operations. For example:

*I understand the concerns [of trust], but that just means we've got to do our job in showing these tools are valid before we actually apply them to the case. … I do believe that having appropriate validation data and showing that you don't have to see in the black box to see that it's reliable. … I think largely revealing source codes is just a tactic …. It's a waste of time, but you know what, knock yourself out, here it is as long as it's protected (LM#1).*

*The problem with validation is I don't have a perfect world [and] validation is subject to some limitations based on what I fed it. … It doesn't mean the validations are not important. They are, but they are only black box validations. I don't know what's in the box. … [That said,] I'm a big proponent of intellectual property, but that's not necessarily for courtroom use. … [In] the perfect world, if you're dealing with people's lives in the courtroom, knowing everything about how decisions are made is a better approach (LM#3).*

When algorithms are based on AI/ML, however, participants were receptive to the idea of using these, particularly if validation testing demonstrated superior performance. None of the participants expressed concern over the opaqueness of the algorithms and the inability to disclose source-code, provided there was adequate validation demonstrating its performance. One participant (LM#2) recognized the difficulties with truly understanding the full limits of a black box system; however, this participant's concerns were mitigated as long as “best efforts” were made to explore these issues during validation and the use of the system was confined by the limits of what was tested. Another participant (LM#3) expressed caution if the limitations are not fully understood. For example:

*I can test the black box and show it's fit for purpose. … Here's my acceptance criteria. I do my testing. It meets the criteria. It works. It's fit for purpose …. So, you can't turn over source code, [well] I didn't really see that as being a real problem before. … If it provides a better value of results, which I should show through my validation, my ongoing testing, I should always be picking the one that's better (LM#1).*

*I don't think using it is a bad thing, as long as you know the limitations. If we don't know those limitations, taking it to court then could cause more damage than good, and that's a problem. Those limitations have to be understood before it's actually used (LM#3).*

When asked about regulation of algorithms, the participants recognized the need for better coordination and guidance to establish best practice and minimize duplication of efforts; however, they stopped short of suggesting full regulation. All three participants considered full-fledged regulation as potential overreach and causing other political and bureaucratic challenges. One participant considered the value of regulation, in theory, as similar to discussions around the requirement to license analysts and accredit laboratories, but questioned whether regulation of specific algorithms would work in practice (LM#2). Overall, participants seemed to express the view that regulation should come in the form of best practice recommendations and validation data that the legal system can consider within the course of case-by-case litigations. For example:

*I feel that a weakness of our forensic science enterprise is that we don't have a cohesive, guidance mechanism as much as I think maybe we should …. I think [full regulation] would probably be considered by many as an overreach, but the court system in a way should be self-regulating to a point …. I think it's been fairly reasonable so far and I think the defense community is pretty well interconnected that when [issues] come out, they're on top of it and that information diffuses (LM#1).*

*I'm not sure I've got a good answer for that …. I'd love to think [that an oversight regulatory body] was an advantage, but I've seen a lot of places where it gets to be a hindrance really quick (LM#2).*

Finally, when asked what participants would describe as the greatest challenges facing the operational use of computational algorithms for court purposes, all three participants pointed to resources—specifically, resources to maintain current caseload requirements while enabling the examiners to gain the foundational training and education to fully understand the systems, validate the systems, and integrate them into day-to-day workflows. One participant (LM#2) offered a detailed description of the competing priorities and challenging decisions laboratory managers are faced with when choosing where to direct their focus. This participant went further by expanding on several other elements that would need dedicated resources to support the implementation of an algorithmic tool, such as the peripheral data management and infrastructure requirements. Another participant (LM#3) highlighted the challenges with developing the algorithms and ensuring they have the proper datasets to start with, which can be challenging given privacy issues preventing open sharing and coordination between public and private institutions. For example:

*Resources. To stay on top of how quick things are developing, it's taking more and more resources. We all have backlogs and we're focusing on those. To take people off of [casework] to train them, then get these new things up to speed and implement them and then change people's minds [takes resources] (LM#1).*

### 3.2 Prosecutors

#### 3.2.1 Background & experience

Three prosecutors participated in the study—one male and two female. All prosecutors are actively working in large metropolitan jurisdictions in the United States and have between 17 and 40 years of experience litigating criminal cases involving forensic science. Each participant serves as the lead prosecutor specializing in litigating forensic science issues within their jurisdiction, including directing and training other litigators on issues related to forensic science. Participants' experiences span across a broad scope of disciplines, including both pattern evidence (e.g., fingerprints, handwriting, firearms), trace evidence (e.g., microscopy), and DNA, as well as across a range of different types of cases, such as street crime, sexual assault, and homicide. One participant expressed experience handling appeals related to forensic science all the way up to the Supreme Court. Participants’ experience litigating algorithms primarily involved those related to probabilistic genotyping algorithms for DNA. Two of the three participants had experience litigating probabilistic genotyping algorithms as part of admissibility hearings. The third participant had experience litigating probabilistic genotyping algorithms “on paper” without an actual legal hearing.

#### 3.2.2 Interpretation & reporting practices

All three prosecutors expressed the perspective that categorical reporting in pattern evidence disciplines using terms such as “Identification” or “Individualization” was the most appropriate and preferred means of expressing conclusions and they disagreed with the claims that those terms imply “absolute certainty.” Participants expressed the perspective that they are both appropriate and easily understandable. Two of the participants agreed that there should be limitations related to those claims, such as not asserting 100% certainty and “to the exclusion of all others” (P#1 and P#2); however, none of the participants expressed any reservations about forensic practitioners providing their opinion on matters related to source attribution (i.e., that a specific individual or item is the source of a questioned impression). For example:

*I don't think saying identification implies absolute certainty (P#1).*

*I don't have a problem with the use of a categorical response. It's easy to understand. It's easy for the jury to grasp, and I believe that it is the true opinion of the scientist who's giving us that opinion (P#3).*

Participants were not completely opposed to probabilistic reporting, in general, however. Participants’ have been exposed to probabilistic reporting through DNA and they all feel it is appropriate in that context, primarily because there is a quantitative basis to the probability and the participants have a general conceptual understanding of how the numbers are produced. In pattern evidence, however, one participant was ambivalent and deferential to the practitioners (P#1), two participants expressed concern that probabilistic statements would be more confusing to interpret among fact-finders (P#1 and P#2), and one participant questioned whether there is a scientific basis to such probabilistic statements (P#3). For example:

*So obviously probabilistic language has been used in reporting DNA results forever …. I don't have any information or knowledge as to how something similar would be done in a pattern discipline …. I would be open to considering it (P#1).*

*A probabilistic conclusion is a lot looser and as a result is much less clear what that means (P#2).*

Overall, participants generally considered the benefits of categorical reporting as being its clarity and simplicity to express and understand. One participant added that an additional benefit is the certainty categorical expressions provide to the opinion, but also noted that it is just one small piece of the overall case (P#3). None of the participants expressed any significant risks to categorical reporting; however, two of the participants reasserted their concern over probabilistic reporting as creating additional complications to the conclusions. For example:

*I think it gets messier the more you start complicating the conclusions in pattern matching disciplines (P#2).*

*The benefit for categorical is the certainty of the opinion (P#3).*

When responding to concerns raised by practitioners as it relates to probabilistic reporting, participants agreed with the risk that it would be confusing to lay fact-finders and believed it was appropriate for them to take this into consideration when debating how to express their conclusions. For example:

*I think that they should be worried about it to a certain extent. They should be cognizant of whether what they are saying at trial is an accurate description of their opinion (P#1).*

However, participants were less sympathetic to practitioners’ expressing concerns that defense attorneys would use probabilistic reporting to create “reasonable doubt.” Although one participant speculated the practitioners were concerned that defense attorneys would attempt to unfairly undermine their opinion with illegitimate attacks (P#1), which could be in the purview of the analyst to be concerned over, the other two participants expressed the perspective that practitioners should focus on what is scientifically appropriate and leave it to the litigators to argue their cases (P#2 and P#3). For example:

*A defense attorney has an obligation to defend the interests of their clients. So, they can take anything in a case and try to create reasonable doubt. That's their job (P#2).*

When responding to questions raised about the role and duties of experts and the limits of their testimony, all three participants were clear that they expect the expert to accurately and impartially convey their opinion and limit their testimony to what is supported by the science. For example:

*The roles and duties of forensic experts are to test the evidence and follow their rules and the best practices within their discipline and to accurately and impartially convey those opinions (P#1).*

*A scientist, in my opinion, should give their opinion as to what the science can say (P#2).*

When asked about whether participants find it acceptable for experts to express their opinion in court without disclosing the underpinnings or statistical data to support those opinions, the participants were generally consistent in their response. Two participants responded by referencing governing evidentiary rules in their jurisdictions (P#1 and P#2) and all three participants suggested it is not required in their viewpoint, although it would not be the best practice to elicit the opinion without providing that foundation. For example:

*There are specific rules of evidence that govern expert testimony in any jurisdiction, and they differ jurisdiction to jurisdiction. [In my jurisdiction], technically the expert doesn't even have to discuss the basis of their opinion. But they can be asked about it on cross (P#1).*

One participant expanded on this question by suggesting courts might tend to be more flexible when testimony is introduced as technical expertise versus scientific expertise and pointed out a growing debate as to whether pattern evidence might be better when presented under this framework. For example:

*I think you're seeing a trend, particularly in microscopic toolmark evidence for firearms where the cases are being argued with technical expertise … and you're seeing some more challenges when it's being offered as scientific. So, it's an interesting question. It's a bigger question, I think, that is going on right now in the community is whether or not some of these pattern matching disciplines should be offered more as technical expertise rather than scientific experts to use, because both of them are legitimate to offer into evidence as expert opinion (P#2).*

Finally, when asked what participants would describe as the greatest challenges facing the pattern and impression evidence disciplines as it relates to examination and reporting methods, the responses were varied—one participant pointed to understanding issues concerning the science (P#2) whereas the other two participants pointed to lawyers and other partisan attacks attempting to undermine forensic evidence overall (P#1 and P#3). For example:

*I think it's a bigger issue that's happening in the community, is to understand what the conclusions are and what the limitations are, and to ensure that we're staying within those boundaries (P#2).*

*I think the challenge is that practitioners and people like you are attempting to appease the defense bar and that's never going to happen …. You are never going to satisfy the defense bar because we are in an adversarial system. … So, I think that the challenge is trying not to fold in the face of that kind of pressure (P#3).*

#### 3.2.3 Use of algorithms

Prosecutors offered varying perspectives as it relates to the use of algorithms in court and the benefits and limitations of them. One participant objected to the use of algorithms in pattern evidence disciplines, claiming they did not believe algorithms were necessary and would unnecessarily confuse and complicate the testimony (making it more challenging for lay fact-finders to interpret) (P#1). Another participant was more skeptical, suggested algorithms could be useful to provide weight to analysts’ conclusions, but cautioned against blind reliance on a computational algorithm without ensuring it is sufficiently valid and appropriate for the intended use (P#2). The third participant was more receptive to the use of algorithms, suggesting algorithms could be useful as a means of enabling the expert to be more efficient and delegate computational tasks to the algorithm that would otherwise be impractical to accomplish in a reasonable timeframe solely by the human, but questioned whether a computational algorithm similar to DNA is even possible for pattern evidence disciplines and expressed concern over how to effectively explain the algorithm to lay fact-finders. For example:

*I think it would overly complicate things and I would not be in favor of it at this point (P#1).*

*[Algorithms] allow the scientists to do computations in seconds that would be undoable in a human timeframe, and so it gives you way more information and helps you weigh the evidence. … I think it's working very well with the DNA [but] I do not see how we establish the numbers or the levels of confidence in pattern matching (P#3).*

When asked about concerns over how algorithms can be trusted for use in court, including issues concerning the disclosure of source code, participants were generally consistent in their viewpoints. On the broader issues of trust, participants tended to be deferential to the forensic experts. On the issue of source code disclosure, although some participants did not feel it was necessary, they all expressed support for disclosure if requested by the defense under terms of confidentiality or protective order. For example:

*[I]f it's scientifically valid and the scientific community is saying this is good science, then as a prosecutor, I'm behind it (P#2)?*

*I'm all in favor of giving the defense every tool that they need to investigate the algorithm (P#3).*

When algorithms are based on AI/ML, however, participants recognized the opaqueness of the algorithms as a potential issue. Although they generally believe AI/ML algorithms would be admissible under existing admissibility standards based on validation data, two participants recognized the potential challenges to admissibility on a constitutional dimension (P#1 and P#3). None of the participants, however, believed the algorithms would be wholly inadmissible, particularly if they were able to explain details about how the algorithms were developed (e.g., parameter selection, training data, etc.) and validated. For example:

*Who am I going to call as a witness at a [admissibility] hearing to explain how this system works that I'm trying to show meets the admissibility standard for my jurisdiction (P#1)?*

*I would think that you would test that kind of algorithm the same way you do any other technology by using known samples. … I can see the confrontation issue. I don't see a due process issue, but I can see the argument that would be made (P#3).*

When asked about regulation of algorithms, the participants were generally deferential to the forensic science community, but were conflicted on whether the legal system was an appropriate means of regulation. One participant believed the legal system was not the appropriate means of regulating algorithms (P#1). Another participant believed the legal system was an appropriate means of regulating algorithms, along with guidelines established by the scientific community (P#2). The third participant recognized the benefits of regulation, but expressed concern that many bodies composed of non-scientists often get “hijacked” by members with alternative agendas (P#3). For example:

*I think [algorithms can be regulated] in the same way that forensic science is already being regulated. It's being regulated through best practice committees and through the court system, and I think that those are putting sufficient limitations around forensic science in general, and that would apply the same with algorithms (P#2).*

*I think that regulation in a reasonable way gives everybody confidence in the science …. [However,] I'm not sure what that regulation would look like, and I'm not sure how, for lack of a better word, political, as opposed to scientific, that regulation would be (P#3).*

Finally, when asked what participants would describe as the greatest challenges facing the operational use of computational algorithms for court purposes, the responses were generally consistent with one another and were concerned that algorithms might create additional challenges when presenting the evidence to lay fact-finders. Participants want to be sure examiners are comfortable and confident in their ability to explain in lay terms to the fact-finders the outcome of that evidence—the more complicated the computational methods, the more challenging it will be. For example:

*I think it's getting stakeholders to understand …. I think [algorithms are] very foreign to people in the entire forensic science community (P#2).*

*I think training the scientists within the labs, to validate it, and to understand it and have confidence in it. I'm not the scientist. I'm using the science and what I want is reliable science that is easy to understand and easy to explain to lay people (P#3).*

### 3.3 Defense attorneys

#### 3.3.1 Background & experience

Three defense attorneys participated in the study—two male and one female. All defense attorneys are actively working in large metropolitan jurisdictions in the United States and have between 20 and 33 years of experience litigating criminal cases involving forensic science—primarily as public defenders. All three participants serve as the lead defense attorney specializing in litigating forensic science issues within their jurisdiction, as well as directing the work of other litigators on issues related to forensic science. One participant specializes strictly on post-conviction litigation. Participants' experiences span across a broad scope of disciplines, including both pattern evidence and analytical disciplines, such as drug identification, fingerprints, firearms, toxicology, dog scent, DNA, etc., as well as across a range of different types of cases, such as street crime, sexual assault, and homicide. Participant's experience litigating algorithms are varied and primarily involve probabilistic genotyping algorithms for DNA, as well as algorithms designed for investigatory purposes, such as “AI policing” and algorithms designed to detect and geolocate gunshots. The general focus of participants' litigation concerns is around issues concerning transparency, validation, and reliable applications of algorithmic tools.

#### 3.3.2 Interpretation & reporting practices

All three defense attorneys expressed a consistent perspective that categorical reporting in pattern evidence disciplines using terms such as “Identification” or “Individualization” is problematic, overstates the value of the evidence, and is not supported by the science. For example:

*If you're going to make an association at all, it should never be categorical, and the association should always allow for the possibility of error or the possibility of a random match (D#1).*

*There's a tremendous amount of concern. Specifically, because there's essentially no scientific foundation for the claims of identification that are being made in almost all of the pattern disciplines (D#3).*

Participants, however, did not necessarily view probabilistic reporting as superior to categorical reporting. The chief concern among participants is the extent to which the conclusions expressed are empirically supported, irrespective if they are reported categorically or probabilistically. Further, one participant expressed the concern that probabilistic reporting, without an adequate empirical foundation, would be misunderstood by fact-finders and misused by prosecutors (D#3). All participants were opposed to the use of probabilistic reporting using numerical references without empirical foundations as to what those numbers were based on. Rather than probabilistic reporting, especially in the absence of validated statistical methods upon which the numbers are based, two participants expressed the view that the optimal approach would be to report associations coupled with clear statements about error rates from black-box studies (D#1 and D#2). The other participant, however, expressed the view that probabilistic reporting would be marginally better (D#3). For example:

*I think the move towards probabilistic language for any forensic discipline that doesn't have reliable rarity data is really problematic. (D#2).*

*There's a significant concern that jurors, number one, don't really understand probabilistic language and that prosecutors will misuse it …. At the end of the day, if there were studies to support that type of language, and if there was some way to ensure that jurors understood what it meant and it was not misstated by either the examiner or by the prosecutor, I think probabilistic language is probably preferable (D#3).*

Overall, participants generally considered the benefits of categorical reporting as the simplicity to express and understand what the expert is attempting to convey; however, all participants believe this is done at the cost of making inaccurate and exaggerated statements that are not supported. On the other hand, the participants generally considered the benefits of probabilistic reporting in that it explicitly conveys limitations, although the extent to which it accurately represents the limitations depends on the extent to which the statements are based on empirical studies. Without well-established validation studies to provide a foundation to probabilistic reporting schemes, especially when numerical quantities are included, could still be problematic since lay fact-finders tend to assume numerical expressions are based on empirical measurements. For example:

*The positive is that [categorical statements] are easy to understand. … But it doesn't really accurately convey the weight of the evidence …. I think very clearly categorical statements overstate the evidence, and that is always a significant danger …. [On the other hand,] I think probabilistic statements they more accurately convey the weight of the evidence, [but] I think they are very difficult for judges, juries and litigators to understand (D#3).*

When responding to concerns raised by practitioners as it relates to probabilistic reporting, participants generally agreed with the risk that it would be confusing to lay fact-finders and believed it was appropriate for them to take this into consideration when debating how to express their conclusions (although one participant [D#1] expressed the view that this is a reflection of the extent to which practitioners do not understand probabilistic concepts). For example:

*I actually do think that the forensic science community does have some obligation for thinking through how information should be accurately reporting. I actually do think it is within their purview because I think that, again, that's something that for years has not been, either intentional or unintentional, but there have been overstatements made in every discipline for years and years and years (D#3).*

However, participants were quite critical of practitioners’ expressing concerns that defense attorneys would use probabilistic reporting to create “reasonable doubt.” Overall, none of the participants expressed a viewpoint that this would be appropriate for them to consider. One participant took it a step further and suggested this finding is indicative of a hidden bias in the criminal justice system (D#2). For example:

*I think [forensic scientists] should stick to the science and let the lawyers worry about what we're going to say (D#1).*

*I would call those results laughable if they didn't concern me so much. … Why are forensic examiners concerned about the outcome of the case? … The fact that 80% of the examiners in a survey are concerned about case outcomes based on shifts of how we report language to me shows the power of the unconscious bias in the criminal justice system (D#2).*

When responding to questions raised about the role and duties of experts and the limits of their testimony, all three participants provided impassioned and consistent responses that forensic scientists base their conclusions on empirical data and be forthright about the limitations of their findings. Some participants went a step further by suggesting forensic scientists routinely fail to fulfill their ethical obligations, in their view (D#2 and D#3). For example:

*The role and duty is to not overstate the science based on a subjective belief in it, or what you've been told by a mentor that isn't verified in science (D#1).*

*Forensic experts have an ethical as well as a legal duty to accurately state the weaknesses and limitations of their forensic method. But forensic examiners don't take this duty seriously. In my 20+ years of litigating many forensic cases, I have never encountered a forensic examiner who took this duty seriously (D#2).*

When asked about whether participants find it acceptable for experts to express their opinion in court without disclosing the underpinnings or statistical data to support those opinions, all three participants were opposed to it. One participant stated a simple “no” without further elaboration (D#2). The other two participants went further to claim it is not legally admissible under existing admissibility standards (D#1 and D#3). One participant openly expressed frustration that such testimony has been admitted in the past and pointed to poor education and poor performance by judges and defense attorneys in the past to have allowed such precedent to be established, but expressed optimism that judges are now beginning to take notice (D#3). For example:

*No opinion should be entered into evidence without a thorough examination for the basis of it. The whole reason that we have a confrontation clause and cross examination is to examine the basis of the opinion (D#1).*

*[Training and experience] are just not a legally sufficient basis for an opinion …. [It's been admitted in the past because] for years and years and years, the defense bar really was, frankly, not educated and did not do a particularly good job of starting to bring to courts the problems with all of these disciplines. So, there's this whole body of case law that's based on either no litigation or very poor litigation (D#3).*

Finally, when asked what participants would describe as the greatest challenges facing the pattern and impression evidence disciplines as it relates to examination and reporting methods, all three participants pointed to the need to conduct the necessary research to provide empirical foundations to the evidence used in criminal cases. One participant (D#1) expressed an impassioned degree of frustration when expressing their viewpoint. This participant seemed to lament the impact of these divided perspectives across stakeholder groups and the lack of enforcement by the courts have had on indignant defendants, suggesting they are the ones that tend to bear the ultimate consequence for what should otherwise be straightforward scientific issues.

*This digging in on the way that this has always been done because of subjective belief that there were no problems with it or because there haven't been tons of wrongful convictions associated with it, is sticking your head in the sand. … The challenge is that courts will . . . . [well, …] I don't know, you know, actually, the truth is there may be no challenge, courts just may not care, because we don't care about the rights of the indigent defendants. In your typical criminal cases, the challenge is scientific integrity. The challenge is trying to claim science when you don't have any (D#1).*

*In pattern matching, I would say it probably continues to be the lack of empirical research (D#2).*

One participant (D#3) went further and described their observation that research tends to be driven by the courts, based on what courts will or will not allow, and this is promoted by forensic scientists looking at court challenges to drive their research priorities. This participant expressed concern that this approach is unscientific and backwards—case outcomes where the admissibility of evidence is limited should not be the factor driving research agendas. Instead, this participant expressed the view that the research should be conducted without consideration of admissibility, then based on those results the courts determine whether the method is useful to the court.

*It was stunning to me that the question that examiners would ask [litigators], essentially “what will the court allow?” And that is not how research should be conducted. It's not what the court will allow. It's what the research shows. … And, then by that same token, I think that, at least in some of the disciplines right now, the research seems to be driven by the courts limiting the testimony. At least in firearms and toolmarks, what I've noticed is a court limits what a firearms examiner can testify to, and then there's a study that comes as a result of that limitation (D#3).*

#### 3.3.3 Use of algorithms

Defense attorneys offered generally consistent perspectives as it relates to the use of algorithms in court and the benefits and limitations of them. All three participants expressed significant caution to widespread adoption of algorithms, specifically over concerns of transparency, validation, and operational uses of algorithms. One participant summarized by stating “that's a complicated question” (D#3). Overall, all three participants were supportive of the use of algorithms, in theory, because, on the one hand they have the potential to provide an empirical basis to examiners' claims, to more accurately reflect the strength of evidence, to promote greater objectivity and consistency in examination results, and to enable examinations to be performed more efficiently. However, on the other hand, all three participants expressed concerns over transparency, validity, and reliability of algorithms when applied operationally. Participants' greatest concern was the lack of transparency surrounding the use of algorithms in criminal justice—specifically when algorithms are used from commercial vendors with proprietary software—which mask the underlying assumptions, parameters, and limitations of the algorithm. Without those details, participants' expressed concern that forensic scientists would apply algorithms operationally without fully understanding their limitations and the conditions upon which they might not be appropriate while at the same time “blindly” relying on the output as if it were factual. For example:

*The greatest benefit would be is that you move away from unsupportable categorical claims into something that has some empirical basis to it and that you would actually have a number that's based on a valid statistical database, a population frequency database that is transparent and known. … [But,] I'm never not going to be concerned about proprietary software being used in these circumstances (D#1).*

*I think, when algorithms replicate the ability of human examiners in their interpretation, I'm much more comfortable with that use of an algorithm. … [However, I am concerned that] inevitably they will be used in the criminal justice system in a role that far exceeds what I'm calling for (D#2).*

When asked about concerns over how algorithms can be trusted for use in court, including issues concerning the disclosure of source code, participants were consistent in their responses and renewed their calls for transparency and greater oversight. All three participants asserted that disclosure of source-code and access to the algorithm and underlying software application to enable them to test was key to gaining trust. One participant went a step further calling for the creation of an independent body of academic experts to assess the algorithm and oversee its operation in casework (D#2). None of the participants expressed a viewpoint that proprietary interests would be at risk if source-code were to be disclosed, particularly under conditions such as a protective order from the court, and each of the participants pointed to civil litigation as an example of courts applying disparate treatment of source-code disclosure in civil litigation versus criminal litigation. One participant expressed the viewpoint that prosecutors shouldn't be using software for which they cannot give access to the source-code and underlying software (D#3). For example:

*What would I need to be comfortable with widespread use and acceptance of an algorithm in the criminal justice system? First, I would need source code. … Developers should not work in any forensic space where the results of their algorithm operation are intended as evidence unless they are willing to publicly disclose their code. … Second, I would need some kind of oversight board—a team of neutral academic experts—provided with the time and resources to analyze the code, stress test it, and publish understandable reports about the assumptions underlying the code, the limits of operation based on stress testing, recommendations for improvement, and recommendations for testimony caveats based on their work. … Third, a pilot period of years, during which a limited deployment in casework is constantly reviewed by the neutral academic team to make sure that the system is being used as intended and that experts do not misstate the value of the evidence in court (D#2).*

*If prosecutors are going to offer this service, then they should be prepared to turn over the discovery, and the discovery that I'm talking about in this context is the access to source code and the software, as well as all validation information and et cetera (D#3).*

When algorithms are based on AI/ML, however, one participant found it challenging to envision how these types of algorithms would be admissible (D#1). The other two participants, however, did not expressly object to the use of these types of algorithms, but re-enforced their concerns over the importance of transparency, accessibility, and oversight when these algorithms might be used (D#2 and D#3). For example:

*You can't have somebody who just turns on the machine and you're coming in and testifying. If we don't know exactly how the machine works, why it works, what its error rates are, how it was developed and why, then it should never be used in criminal court. … It is, in my view, a sixth amendment violation, no matter what—if you were denied your right to confrontation, you were denied due process of law (D#1).*

*I think [admissibility] would have to be on a case-by-case basis …. I think the complication comes in when we try to find out what's behind the black box (D#3).*

When asked about regulation of algorithms, all three participants referenced the need for an independent oversight body responsible for assessing function, validation, operations, and testimony. One participant suggested it should be a neutral government entity, similar to the United States Food and Drug Administration (D#1). Participants also referenced standards set forth by the Institute of Electrical and Electronics Engineers (IEEE), suggesting a similar type of requirements should be established for the development and validation of software applications developed for criminal justice purposes. Finally, all participants expressed strong rejections to the idea of the legal system being an effective means of regulation. One participant went so far as to claim the legal system has “utterly failed” to regulate forensic science in general and therefore expressed no confidence it could not be trusted to effectively regulate algorithms (D#2). For example:

*There should be independent bodies to assess their function, their validation, how they operate, who should be able to review training data, who should be able to require the appropriate caveats during testimony, who should be able to require that proper standards are used to develop [the algorithms], whether it's IEEE standards or others. … [The notion that the legal system could regulate algorithms is] really a laughable position. The criminal justice system has proven to be an utter failure as gatekeepers of forensic evidence (D#2).*

Finally, when asked what participants would describe as the greatest challenges facing the operational use of computational algorithms for court purposes, participants referenced the need for increased investment in education for practitioners that will be expected to use the algorithms operationally, and for judges who will be expected to assess the admissibility of the algorithms. For example:

*[The greatest challenge] is these non-scientists understanding what this machine is doing and the limitations of what the machine [and] results are. [Further,] having a forensic examiner, very few of which have a background in computational … anything, explaining accurately to these lay people what this machine is doing and the limitations of what this machine is doing (D#3).*

### 3.4 Judges

#### 3.4.1 Background & experience

Three judges participated in the study—one male and two female. One participant (J#1) is a sitting federal judge in a large metropolitan jurisdiction, having served for over 25 years as a federal judge and presiding over a wide range of criminal and civil cases, including issues concerning forensic evidence. Prior to being appointed as a federal judge, this participant served as both a federal prosecutor and a criminal defense attorney. Additionally, this participant serves as an adjunct professor at an Ivy League law school, has co-authored books, published numerous articles, delivered several presentations, and served on several professional committees, including those related to forensic science. Another participant (P#2) is a sitting state district court judge, having served six years of the current elected term.^2^ Prior to being elected as a state district court judge, this participant served as a defense attorney, including experience as an assistance state public defender, with extensive experience litigating complex felony cases largely involving forensic science evidence—including issues related to the discovery of source code and admissibility of alcohol breath testing instruments. This participant has provided several presentations and trainings and has served on professional committees on issues related to the use of forensic science in courts. The third participant (J#3) is a former federal judge in a large metropolitan jurisdiction. This participant served as a federal judge for over seven years before stepping down in late 2018 to return to private practice and focus on issues in commercial litigation, including issues involving technology and artificial intelligence. Prior to serving as a federal judge, J#3 served as a litigator in private practice for over 20 years and as the deputy assistant attorney general for the U.S. Department of Justice. While this participant has experience presiding over a wide array of criminal and civil cases, this participant has specialized experience on issues concerning artificial intelligence and algorithmic tools applied to the criminal justice system, having authored a book on the topic, provided several presentations and trainings, and served as an adjunct professor at a reputable law school on issues related to the use and presentation of quantitative methods by litigators, courts and policymakers as they advocate legal and policy positions.^3^

#### 3.4.2 Interpretation & reporting practices

The two participants who provided responses to these questions, (J#1) and (J#2), expressed the perspective that categorical reporting in pattern evidence disciplines using terms such as “Identification” or “Individualization” was challenging because it conveyed a degree of certainty that has not been well established.^4^ These two participants suggested categorical reporting was akin to expressing an opinion “to a reasonable degree of scientific certainty,” and expressed the concern that those statements do not have clear meaning to lay fact-finders and not only mask the level of subjectivity involved in the examination, but also convey a level of certainty that exceeds what can practically be achieved. One participant (J#1) goes further to suggest that the means by which forensic science conclusions are reported is a factor that has contributed to the erroneous conviction of innocent people. The other participant (J#2) expressed a view that categorical statements involving source attribution could be acceptable provided that the examiner could provide adequate foundation to support such a claim and the relevant uncertainties and limitations of the examination are conveyed. However, this participant goes further and openly questions whether it is practical to establish such a foundation and demonstrate that the uncertainty is such that a categorical statement of a source attribution is warranted. For example:

*As I think many people know, bad forensic science has been an element in the conviction of innocent people …. One of the reasons that those inaccuracies [in forensic science] came about [was] because the science itself was much more subjective than was represented to courts and to juries, [and] because they were presented as being certain conclusions. … There's almost no part of science that can claim certainty. If you talk to physicists or chemists or whatever, they won't claim that. Yet here it is, in effect, being claimed by forensic science (J#1).*

*I think it's very challenging to use [categorical statements] for purposes of how to report a result. … How do I know that there's the foundational science to be able to say that, as we're doing this comparison, that I can make the statement, “yes, this impression came from this source?” We get into [things] like, “well, it's a match.” Well, okay. It may be, [but] how do you know that (J#2)?*

Participants considered probabilistic reporting as an improvement over categorical reporting; however, participants cautioned that it may not necessarily address all of the concerns. One participant (J#1) suggested probabilistic reporting is an improvement to categorical reporting, but expressed a concern that lay fact-finders would not be able to meaningfully interpret what was being conveyed or scrutinize the validity of the underlying statistical methodology upon which the probabilistic statement was based. Another participant (J#2) expressed initial reactions of being averse to probabilistic statements given the potential to be misunderstood. However, after reflecting on the issue more, this participant expressed a view that probabilistic statements could be advantageous to categorical reporting because they cause the fact-finders to pause and think through the nuances of what is actually being conveyed rather than relying on familiar colloquial definitions of terms that are often used when reporting categorically (despite such terms having a specific technical definition in the respective forensic discipline). This participant went further, however, to express the view that probabilistic statements should include numbers, and those numbers should be accompanied by a statistical model to provide the source of those values. For example:

*Well, I think [probabilistic statements] would be an improvement, but I worry again about two things. First, the ability of judges and juries to really scrutinize, in a meaningful way, when someone says it's this probability or that probability. And secondly, the validity of the underlying statistical methodology used, which varies considerably …. Nevertheless, I think expressing it as a probability would still be better than expressing it as a certainty. But I do think it still has a great potential to confuse (J#1).*

Overall, participants generally considered the benefits of categorical reporting as its simplicity of the statements; however, they also expressed the concern that such statements are not well-defined and are often interpreted to mean something that is not supportable. One participant (J#1) stated “the greatest risk with categorical is it's stated as a certain thing, and that's just not true.” Another participant (J#2) believed such statements “do not always align with what lay persons' understanding of the definitions would be.” Participants viewed probabilistic reporting as being an improvement over categorical reporting in the sense that probabilistic reporting is more defensible and easier to define, but participants still expressed concern. One participant (J#1) questioned whether statistical methods are appropriate when there is a high degree of subjectivity, and also noted “that the recipients, the judges and juries who are hearing these opinions are very rarely people of statistical sophistication and so they may give a greater weight than it really deserves.” Another participant (J#2) cautioned that “probabilistic models have ways in which they can be misconstrued.”

When one participant (J#1) was asked how such testimony should be permitted, the participant responded, “it varies from discipline to discipline.” The participant elaborated by reference to a prior case opinion they authored:

*The best way to answer that is by talking about an opinion I wrote, United States v. [REDACTED], where the question was whether there was a match between the marks on the bullet and cartridge from the gun …. Originally, I asked the expert, “what's your error rate?” and he said “zero.” I said “zero?” And he said, “because I've never testified in a case in which the defendant wasn't convicted.” … Put[ting] aside that non-sequitur for the moment. More to the point, in the end, what I allowed in that case was for the expert to show great big blow ups of the marks on the bullet and cartridge and the marks on the gun, and to point out some of the similarities between those and to then express the opinion that it was more likely than not that this came from the same gun. That's as far as I felt one could go without misleading the jury. I'm not sure today I would even go that far because I've seen many more examples of wrong, inaccurate forensic science, but I certainly wouldn't go any further than "it's more likely than not in my opinion that this bullet came from that gun.” Of course, it depends on the forensic discipline. When you're talking about, for example, microscopic hair analysis, the error rate is extremely high and I wouldn't allow that in. I might have back at the time of [this case] considered allowing it in the modified way I indicated, but no longer. So, it varies from discipline to discipline. [For fingerprints in particular,] they are not bad forensic science, but they're not DNA either. … I think I would not exclude it. … I think that the evidence is there that fingerprint evidence is not junk science and that with proper limitations, it can be received in evidence. [For example, I would probably allow] the expert to blow up pictures of the two fingerprints to be shown to the jury and point out some of the similarities between those and then express the opinion that it was more likely than not that this [print] came from the same [individual]. … I [also] think maybe you should require as part of [the expert's] direct testimony, to say, “now I've arrived at that [opinion] through experience, not through some sort of scientific formula” (J#1).*

When responding to concerns raised by practitioners as it relates to probabilistic reporting, participants agreed that probabilistic reporting would be more confusing to lay fact-finders, but they did not express the view that the issues were insurmountable.^5^ One participant (J#1) suggested that the risks for confusion, which probabilistic reporting might entail, would be less worrisome than the view fact-finders often take with categorical reporting. The other participant (J#2), while recognizing the potential for confusion, expressed the view that, on the other hand, probabilistic reporting might be useful to cause people to pause and think through the nuances rather than rushing to judgment based on colloquial uses of terms that experts use categorically. For example:

*Well, I do think there is a potential for confusion, but it's not as bad as the view that the jury will take otherwise, that it's an absolute fact. When the jury hears the opinion it's a match, their natural reaction is to say, “okay, it's been scientifically found that it's a match. Period.” (J#1).*

*At first, when I started working with them, I was like, this is way too confusing and there's no way we're going to be able to do this in a way that's meaningful to people, but in some ways, I think there are some things about it that makes it more approachable (J#2).*

However, participants were less sympathetic to practitioners' expressing concerns that defense attorneys would use probabilistic reporting to create “reasonable doubt.”^6^ Participants disagreed with the practitioners’ concern and expressed concern that it would be a factor taken into consideration. One participant (J#1) suggested that this indicates practitioners do not have faith in juries and offered a reminder that the determination of reasonable doubt is what the judicial system is all about. The other participant (J#2) suggested that this indicates a general fear practitioners have for defense attorneys. For example:

*I'm not sure what is meant by the objection that this might create a reasonable doubt. Well, that's what the system is all about, is finding out whether there is, or is not, a reasonable doubt. It sounds like those respondents didn't have much faith in juries (J#1).*

*I think we would need to stop being afraid of defense attorneys. I really do think that we just need to stop that nonsense. These numbers can be misused by everybody because they aren't being understood properly. I don't think a lot of it is even intentional. I just think that it is what it is. So, I think misuse happens for all sorts of reasons and it doesn't have to do with what side you're on. So no, I don't think that it should be a reason that we should not look at [probabilistic reporting] (J#2).*

When responding to questions raised about the role and duties of experts and the limits of their testimony, participants expressed the view that results should be reported in an accurate manner with appropriate foundation to base such conclusions and that the experts should be forthright about the error rate and limitations of the findings. As one participant noted, without being forthright about this information “the jury is deprived of information that is available, that is out there” (J#1).

When asked about whether participants find it acceptable for experts to express their opinion in court without disclosing the underpinnings or statistical data to support those opinions, one participant (J#1) stated “no” without further elaboration. Another participant (J#2) admitted to have struggled with this question, stating that the rules of court require the expert to provide the foundational support for their opinions, but experts should be answering the questions put to them by the lawyers. Instead, this participant, suggested that experts should be more proactive about disclosing these foundational issues earlier, such that it is laid out before the court process, such as on the report that is provided to both parties, which, in turn, would enable either party to further discuss during court as they deem appropriate. The third participant, (J#3), stressed throughout the interview that “the means to the end matter”—both as it relates to expert testimony and the use of algorithms—and that an opinion that is expressed without the reasons for that opinion would be considered *ipse dixit* and cannot be relied upon. For example:

*My view is that [would be] called ipse dixit—"it is because I said it is,” and, under the Daubert standards, the Supreme Court standard for the admissibility of an expert opinion, that's not allowed. … Every judge should require that an opinion be backed up by the reasons for the opinion and that, if an expert gets up there and says, “based upon my experience, this is just the way it is,” … I would say that that's an unreliable opinion (J#3).*

Finally, when asked what participants would describe as the greatest challenges facing the pattern and impression evidence disciplines as it relates to examination and reporting methods, participants pointed to multiple issues.^7^ One participant, (J#1), responded with the need for “good, blind, scientific testing” to strengthen the scientific rigor underlying many forensic science disciplines. This participant, (J#1), elaborated that the “greatest failing” is that many forensic sciences, with the exception of DNA, have been developed by police as investigative tools and began to be introduced as hard evidence without subjecting it to serious testing. The other participant, (J#2), expressed the view that the greatest challenge is to ensure, irrespective of how those results are reported, that everyone understands how to properly interpret the value of the evidence.

#### 3.4.3 Use of algorithms

The judges offered generally consistent perspectives as it relates to the use of algorithms in court and the benefits and limitations of them. All three participants expressed views that algorithms can be helpful—particularly for purposes of augmenting the expert to reduce the degree of subjectivity in the analysis and performing tasks that humans would otherwise be incapable of doing. However, participants also expressed caution about the desire to rely on algorithms without ensuring that there is transparency into how the algorithms operate and clear understanding of the limitations of the systems. One participant (J#1) expressed concerns citing the lack of transparency, logistical, and financial challenges often prohibiting defense counsel to meaningful scrutinize algorithms used in the criminal justice system. Another participant (J#2) expressed the view that the lack of transparency around these algorithms not only creates the opportunity for misuse, but also perpetuates a culture of distrust that already pervades the adversarial system, which ultimately “erodes confidence in the analysis as well as potentially in the system itself.” The third participant (J#3) suggested there needs to be a national conversation on how to create trustworthy and reliable algorithms, and what that means, as it relates to uses for individual liberty determinations. For example:

*I think algorithms can be helpful, to a degree, if they are totally transparent …. I think really good algorithms could reduce the subjective portion of the analysis …. [However,] some companies are obscuring inquiry through trade secrecy laws, but even where that doesn't operate it's very hard for even defense counsel [to review]. … Even in those states where the trade secrecy law objection is overruled, they have to hire an expert … [but often] there's no money available to hire that kind of expert (J#1).*

*I think that algorithms are here to stay …. There's a great potential [with algorithms], [if] done correctly, to create criminal justice reform to a degree that we've never seen before. … [T]hey have an ability to take out some of the human biases that have plagued the criminal justice system. … [B]ut there are certain risks. … What we need is a national conversation on what that means and how to create trustworthy and reliable algorithms that can be used for individual liberty determinations. That's where the rubber meets the road (J#3).*

When asked about concerns over how algorithms can be trusted for use in court, including issues concerning the disclosure of source code, participants were consistent in their views, echoing their prior concerns about transparency and asserting the need for access to source code. Participant (J#3) expanded on the concept of trustworthiness by pointing not only to reliability testing, but also whether the design of the system corresponds to a concept of “fairness.” This participant argues, on a Constitutional basis, that “the means to the end matter” and the “means” are contained within the source code. For example:

*I think [source code] absolutely should be disclosed in every case. I don't see how you can tell the judge, let alone the defense lawyer, [they] can evaluate whether it's a good algorithmic approach or not if you don't know how what went into the source code and what its components were, how they were arrived at it, and so forth. And, give me a break about trades secrets. I appreciate that companies like to make money, but we're talking about human liberty here, and that has to trump any concerns over trade secrets (J#1).*

*I personally think that it should be open source codes, period. … I respect the fact that there's intellectual property issues and so forth that's around that, but I think that we have mechanisms to assist in protecting that (J#2).*

*I think that what it means to be trustworthy is very close to what it means to be reliable, but I think it incorporates something else. Reliability is simply, “does the tool work as it is intended to work?” … Trustworthy certainly incorporates that, but it [also] incorporates something else, which is a concept of fairness. … In my view, if an algorithm is going to be used for a liberty-based decision, a criminal defendant is entitled to have access to the source code, and I would say for an adequate defense, just as a criminal defendant is entitled to the experts that he or she can demonstrate are needed to put on an adequate defense, that same individual is entitled to an expert who can then help them analyze the algorithm (J#3).*

When algorithms are based on AI/ML, however, participants were not completely opposed to their use; however, they did express views that were even more cautious given the lack of transparency. When asked whether the opaqueness of these types of algorithms could present an issue from a Constitutional dimension, such as Due Process or Confrontation, two participants (J#1 and J#2) did not believe, in general, it would be wholly excluded, but did express concern over their use nevertheless. The third participant, (J#3), expressed the view that understanding the design of the algorithm is absolutely critical, and in the absence of such information the evidence generated by the algorithm should be excluded. Ultimately, this participant was unwilling to accept that the conceptual innerworkings and design of the system is incomprehensible, despite the apparent black box nature of the source code file itself that is often the case with AI/ML algorithms, and expressed the view that giving up the ability to understand these issues would be giving up important Constitutional principles. For example:

*At a minimum you need to know what the error rate is …. But, also, I'm a little suspicious about any notion in the legal system where we say, “we don't know why X causes Y, but we know it does.” … I think a lot of scientists, a lot of lawyers, would be very skeptical about the use of that because ultimately the law depends on reason, not on assumptions …. So, I am skeptical of the black box approach (J#1).*

*They fascinate me and scare me all at the same time. I can't say that access to the source code is the “be all and end all” of anything. … [B]ut I don't even know how to begin to assess that stuff. … I really think that if we're going to start using them, that we need to figure out what it is that we do need for purposes of making sure that there's essentially buy-in from everybody, that this is why this is working and that we can have some check on the fact that it is working in the way that we believe that it's working (J#2).*

*Understanding how the instrument was designed is absolutely critical to understanding the calibration of the instrument and the choices. … [Ultimately,] I think there are serious due process issues with a defendant being denied access to understanding information that underlies a tool being used for liberty decision (J#3).*

When asked about regulation of algorithms, the participants expressed views that spanned across the forensic sciences more broadly, not just algorithms, that there should be regulation. Although participants had different views on who and how that regulation should be done, participants did not feel the legal system was effective as-is. For example:

*Yes, [but] not justalgorithms. I think there is a real need for an Institute of Forensic Science staffed by a high-level scientists who could tell us with the neutrality that we deserve, this is good forensic science, this is bad forensic science, this is possible forensic science but it has to be improved and here's how to go about improving it. … I don't think the legal system, ultimately, is well positioned to regulate forensic science. Judges know beans about science. Lawyers know beans about science. The natural thing when you have that kind of problem is to turn it over to the people who do know about science, the scientists (J#1).*

*Yes, [but] the by whom and how is a much harder question …. [Whether the legal system is an appropriate means of regulating forensic science,] no, [but] I will also say I'm not sure the federal government is the place to regulate it either (J#2).*

*In my view, there should be a form of regulation that is for any liberty-based decision. It's a broad question in terms of algorithms and any kind of forensic science, …[but] if it's going to be used for a liberty-based decision for a human being, then they need to meet the constitutional standards, so they should be regulated. … The, how, I think, is extraordinarily complicated, but I don't accept that it can't be done (J#3).*

### 3.5 Other (academic scholars)

#### 3.5.1 Background & experience

Three “other” stakeholders (i.e., academic scholars) participated in the study—two male and one female. One participant (O#1) has over 30 years of experience performing research in forensic science, with the specific aim to provide a more structured foundation to case assessment, evaluation, and interpretation, and served for several years in a chief government role establishing policy governing forensic science practices on a national level.^8^ Another participant (O#2) has over 30 years of experience as an academic scholar at an Ivy League university, primarily focused on research involving human judgment and decision making from a multidisciplinary perspective, including law, psychology, biology, and statistics. The third participant (O#3) has over 35 years of experience as an academic scholar at an Ivy League university, primarily focused on theoretical physics, mathematics, and general scientific issues of public interest. This participant has also served as the president of a large scientific organization comprised of approximately 50,000 members with diverse scientific backgrounds. All three participants are respected in the general scientific community, have doctoral degrees in scientific disciplines, have numerous scientific publications, and have experience serving in senior advisory roles on issues affecting forensic science practices on a national scale.

#### 3.5.2 Interpretation & reporting practices

All three participants expressed the perspective that categorical reporting in pattern evidence disciplines using terms such as “Identification” or “Individualization” was inappropriate and conveyed a level of certainty that was unsubstantiated and outside the realm of what scientific principles can support. One participant recognized the effort that would be involved with promoting such a transition and expressed a perspective that categorical reporting, in the interim, should be accompanied by statements about the limitations of such claims (O#1). The other two participants expressed a much more rigid perspective, suggesting such claims were not scientifically justified and were an overstatement of what can be empirically supported (O#2 and O#3). One participant took it a step further and expressed the view that such claims violated the trust that fact-finders place in forensic scientists and was “immoral” if they made such claims under the auspice of “science” (O#3). For example:

*I think it's clearly not justified scientifically. It's an overstatement of the value of the evidence. We know it's simply not plausible for a discipline, like fingerprinting, that a trained examiner can determine the rarity of the set of features observed [based solely on human judgment] with the precision necessary to know whether it's probability in the population is low enough to support the claim that it’s a unique observation (O#2).*

*I think it is wrong. I think it's immoral to stand in front of a jury and make categorical statements if you are a forensic scientist because the word “scientist” confers in the minds of the jury that you are, well, one way that I heard it expressed is that the words have totemic power. I think it's wrong to abuse that level of trust …. Look, the way I view it, we can make categorical statements, but don't claim it's backed up by science (O#3).*

Participants were not completely consistent with endorsing probabilistic reporting, however. One participant expressed strong views that probabilistic reporting was the path forward (O#1). However, another participant seemed to support probabilistic reporting simply because of the lack of any reasonable alternative and that categorical reporting was not acceptable (O#2). This participant seemed to accept probabilistic reporting as the path forward, but was more interested in how to most effectively articulate probabilistic results to lay factfinders to maximize their comprehension of the information—a topic that this participant believes still requires more research. The third participant, however, expressed views that seemed to reject both categorical reporting (as it is traditionally practiced) and probabilistic reporting (O#3). This participant expressed concern that probabilistic reporting, albeit superior than categorical reporting from a scientific standpoint, would not be well understood by fact-finders. Instead, this participant suggested black-box testing of examiners' performance was the optimal approach, so that examiners’ conclusions can be accompanied by an empirical measure of certainty based on error rate data (O#3). For example:

*I strongly believe that [probabilistic reporting] is the appropriate approach to take …. It is much more scientifically correct and defensible to acknowledge that uncertainty in a probabilistic form (O#1).*

*I have problems with [probabilistic reporting] too, but the problems don't lie on the side of the forensic science community, it lies on the side of the triers of fact. [For example,] I know for a fact, most people don't understand fractions … So, I'm not sure if probabilistic is better, but I know a lot of people are in favor [of it] (O#3).*

Overall, participants generally considered the benefits of categorical reporting as its simplicity to express and understand; however, they all acknowledged that ease of understanding is at a cost of being scientifically valid and transparent about the uncertainty. Participants viewed probabilistic reporting, on the other hand, as being scientifically more defensible, but at the same time, more challenging for lay people to understand and at an increased risk of erroneous interpretations.

When responding to concerns raised by practitioners as it relates to probabilistic reporting, all three participants were sympathetic to the concern that probabilistic reporting would be confusing to lay people. Although one participant (O#3) responded in a way that suggested probabilistic reporting was not the ideal path forward (versus black box testing to derive empirical error rates), the other two participants (O#1 and O#2) did not believe the confusion that would accompany probabilistic reporting was insurmountable or a strong enough reason not to pursue it. For example:

*I think they are right. It may be confusing to a lot of people, but I don't think that's a sufficient reason to go back to an unjustifiable alternative form of reporting (O#2).*

*[I agree,] just ask someone on the corner and say, “I have this problem with fractions. I want you to solve it” and see what kind of reaction you get. So that informs me that for the average person who finds themselves on the jury, a deep understanding of probability is it's like asking them to solve Einstein's equations. It's just not going to occur (O#3).*

Participants were also understanding of practitioners' expressing concerns that defense attorneys would use probabilistic reporting to create “reasonable doubt;” however, participants did not view it as a reason to oppose probabilistic reporting. To the contrary, participants suggested it bolstered the reason to pursue probabilistic reporting if it more effectively represented the certainty of the findings. One participant (O#1) expressed concern that this indicates a deeper cultural challenge that forensic scientists are averse to talking about anything that might undermine the certainty of their findings. Another participant (O#2) noted the irony in the question and highlighted the fact that it is the very job of defense attorneys to highlight anything that should cause fact-finders to doubt the evidence—particularly if the doubt is “reasonable”.^9^ The third participant (O#3) agreed with the practitioners' concerns recognizing probabilistic reporting creates an opportunity to for defense attorneys to abuse it and bolster their arguments, but also suggested categorical reporting that does not acknowledge the uncertainties also creates opportunities for prosecutors to abuse it to bolster their arguments. Considering the risk for both parties to abuse each type of reporting methods, this participant, (O#3), echoed their perspective that empirical measures of accuracy through black box testing is a way to put boundaries around these issues. For example:

*I think they don't want doubt introduced, [and] it scares me actually. It scares me that forensic scientists don't feel confident to talk through uncertainties and anything that is below a hundred percent. We, as scientists, should be comfortable in talking about the limitations of our analysis as much as the strengths of our analysis. It's the job of defense attorneys to introduce reasonable doubt, but it's our job to be sufficiently transparent to allow them to scrutinize the evidence (O#1).*

*[First of all,] creating reasonable doubt is what defense lawyers are supposed to be doing. If there's some reasons to doubt the finding, then the jury should know about them …. [Second,] from my perspective, this portrays a mindset, which is that the goal of forensic science is to produce convictions and anything that gets in the way of producing convictions is a bad thing. I just have a totally different perspective on this (O#2).*

When responding to questions raised about the role and duties of experts and the limits of their testimony, all three participants expressed the view that experts’ number one priority should be ensuring their results that are reported are scientifically defensible. Two of the participants define this in terms of transparency about the uncertainty that might exist to ensure the court has the requisite information to make an informed decision (O#1 and O#2). The other participant (O#3) defines this in terms of ensuring testimony is grounded by measures of repeatability and reproducibility. For example:

*I think the role of a forensic science expert is to assist the court, not the prosecution or the defense but the court, in its evaluating evidence and to use their skill and knowledge that lay people don't have to help evaluate the scientific findings in a way that is helpful to the court—that is transparent about strengths and limitations …. I think it is the role of the court to conduct that final reasoning in the light of the uncertainty that exists (O#1).*

*I think the first duty is to get it right—to say things that are justified scientifically [and] to not go beyond their expertise and not claim more than the science will support. That's duty number one. Do not make unjustifiable claims. Then duty number two is, once you've identified the various claims that might be justifiable, try and choose among them in a way that promotes better understanding for a wider range of people. When in doubt, maybe present the evidence in multiple alternative ways and focus on transparency and a fair characterization of uncertainty (O#2).*

When asked about whether participants find it acceptable for experts to express their opinion in court without disclosing the underpinnings or statistical data to support those opinions, two participants flat out stated “no” without further elaboration or exception (O#2 and O#3). The other participant (O#1) expressed the view that disclosing the underpinnings of the expert opinion is important, but also recognized the dynamics that affect testimony in a court setting. Nevertheless, this participant suggested the foundations for the expert's opinion should be disclosed in the case file so that it is documented and available, if needed. For example:

*I think it is really important to disclose the basis of your opinion. I think when it comes to the actual courtroom, [however,] it depends on so many things—what you actually say in testimony. When it comes to your written statement of evidence and your case file, that contains all your notes, [however,] I think that underpinning has got to be disclosed so at least it should be available for scrutiny by whoever in the court process wants to scrutinize it. I think that when we just give unqualified opinions, it is almost impossible to challenge really, because if you're not giving a reason for your opinion then it just comes down to, “well, that's my opinion” (O#1).*

Finally, when asked what participants would describe as the greatest challenges facing the pattern and impression evidence disciplines as it relates to examination and reporting methods, participants’ responses were quite varied. One participant (O#1) pointed to an on-going narrative that forensic sciences are “in crisis” and implications that they are useless unless perfect. This participant expressed the view that such an aspiration of perfection is unrealistic and fails to recognize the value that many pattern evidence disciplines can give, provided that there is transparency around the limitations and imperfections of the disciplines. Another participant (O#2) pointed to the need for on-going validation of the examination methods and recognition of the limitations of those methods as revealed by validation studies. This participant expressed the view that these validation studies should be on-going and ideally be incorporated into routine casework through blind testing. The other participant (O#3) pointed to resources as the greatest challenge facing the forensic sciences. This participant suggested that the conditions that many forensic scientists are working under is conducive to errors, and calls for greater investment and support of the forensic science community to provide the resources necessary to perform at the level that society expects and needs. For example:

*The greatest challenge that I've observed is actually resources. … I have had a chance to see the conditions that real forensic scientists work under. They're not the conditions that Hollywood tells the public about. The real conditions are often overworked people [and] under-resourced people with no time to get the results out. I mean, that's the real world. To me, that's the greatest challenge to forensic science, to convince our society to put in the resources so that people can do the best job, so that this intuitive expertise that I [believe forensic scientists have], is actually allowed to work without having the pressure that can induce errors (O#3).*

#### 3.5.3 Use of algorithms

The academic scholars offered generally consistent perspectives as it relates to the use of algorithms in court and the benefits and limitations of them. All three participants expressed favorable views of algorithms, in general, but with caveats. One participant (O#1) expressed very favorable views of algorithms for which the underlying operation is understandable and explainable; however, this participant expressed extreme caution when the algorithms are not well understood. This participant went further to question whether it is even practical to fully validate algorithms that are not well understood, or if there is a sufficient legal basis for which to introduce those types of algorithms. Another participant (O#2) recognized the value of algorithmic approaches over human judgment, but conditioned that support on whether the specific algorithm in question was “validated and appropriate,” including assessments in case specific applications. The other participant (O#3) was supportive of algorithms provided they were free of any ties to demographic factors or large characterizations of populations and pointed to algorithms used in “predictive policing” as an example, where the algorithms can perpetuate systemic biases. For example:

*I think algorithms may well be preferable to human examiners giving opinions based upon experience because the use of the algorithm reduces the chances for bias and it may allow better estimation and calibration of that strength of the evidence. … [However,] these models tend to be very complicated and difficult to assess. Algorithms have advantages, but it's going to require a whole new realm of expertise to evaluate them (O#2).*

When asked about concerns over how algorithms can be trusted for use in court, including issues concerning the disclosure of source code, participants all pointed to validation data as the key to demonstrating the performance of the system under various conditions that are representative of the facts of the present case. As part of validation, participants expressed strong views that there needs to be clear understanding of the boundary conditions for which the algorithm performs well, and the circumstances (or combination of circumstances) for which the algorithm might begin to fail. As long as those conditions are well understood, participants suggested that there is reason to trust the output of the algorithm. Participants recognized the importance of transparency in building trust, and disclosure of source code is a key element of transparency. Although all participants encouraged the disclosure of source code to promote greater transparency, none of the participants expressed strong views that the source code *must* be disclosed before an algorithm can be trusted. Rather, participants pointed to the existence of validation studies and conceptual descriptions of how the algorithm operates, along with its limitations, as well as having access to the algorithm for independent testing as being more useful to the typical expert. For example:

*Well, two things: transparency and performance testing. Transparency, because if I were in some sort of legal situation where an algorithm played a role in determining my freedom or even more consequentially my life, I would want my attorney(s) to have the ability to bring their experts to look at the algorithm [and] to make sure that I wasn't a victim of bias. So, transparency is for me, the first thing. The second thing is [that] I want these algorithms tested on a regular basis, looking for failure modes. I want the reliability testing as part of the use of it (O#3).*

When algorithms are based on AI/ML, however, participants expressed views that suggested they were skeptical of whether these algorithms could be validated in a way that fully understood their boundary conditions and limitations in such a way that would be appropriate for court. Although two of the participants did not explicitly reject the idea of AI/ML algorithms (O#1 and O#2), one participant (O#3) opposed the idea altogether. All three participants expressed similar concerns that the level of effort to truly understand the boundary conditions and limitations of the algorithm through performance testing would be impractical to accomplish. For example:

*I think if [the algorithm] is not understood to the developers and it's a total black box, then I struggle to see on what basis that there is fair transparency in the [legal] proceeding (O#1)?*

*Theoretically, it could be acceptable to use these systems if we have reliability testing. [The problem is], the testing has to be large and broad because you don't know where the failure modes are[.] … [That said,] is this type of reliability testing practical? What I've talked about is the ideal. I don't think the idea is actually practical [and] realizable. I don't think you could actually implement it (O#3).*

When asked about regulation of algorithms, the participants recognized the need for oversight, but offered slightly different perspectives. One participant (O#1) suggested that the regulation should be focused on the method—not just the algorithm, which is a narrow part of the overall method—such that regulation addressed the validation of the algorithm as well as the use of the algorithm (including the training and competency of the people, the inputs, and testimony of the results). Another participant (O#2) expressed the need for regulation by an independent oversight body. This participant lamented the current situation where regulation is left to the legal system and expressed strong concerns that the legal system is ineffective at regulating forensic science overall, much less algorithms. The other participant (O#3) felt unqualified to address this question and was cautious to offer an opinion from a professional capacity; however, when asked from a personal capacity, as a citizen and potential consumer of forensic science evidence that could be based on algorithmic tools, this participant stated clearly that they were opposed to the use of any algorithm in court based on machine learning that was a total “black box.”

*It's not the algorithms that need to be regulated, it's the methods, and the methods include the people, the algorithms, the data, and everything else (O#1).*

*Yes, I still think it would be nice if we had a national institute of forensic sciences contemplated by the NAS report in 2009 …. Right now, we're stuck with the regulatory authority being exercised by judges who, for the most part, have not shown a willingness to apply rigorous quality control with regard to validation of forensic science …. So, I'd like to see more federal involvement with agencies that have the ability to make some scientific assessment and set regulations on their own. I think that would be appropriate (O#2).*

Finally, when asked what participants would describe as the greatest challenges facing the operational use of computational algorithms for court purposes, participants offered very different viewpoints. One participant (O#1) highlighted the need for clear understanding of what type of algorithms is being considered because the benefits and risks vary widely, and to ensure scientific debates about the validity and appropriateness of algorithms are done in a scientific setting outside of a specific legal hearing. This participant also pointed out the need for improving education and training for both forensic science practitioners and legal stakeholders on these issues. Another participant (O#2) discussed the need for the development and validation of robust algorithms, but highlighted the challenges associated with their implementation. This participant went a step further and suggested that the move toward algorithms might also necessitate changes around recruitment and selection of forensic science practitioners to include stronger backgrounds in mathematics, statistics, and hard physical sciences that might provide greater exposure and receptivity to algorithmic tools. The other participant (O#3) expressed concerns of the potential for the quality and reliability of algorithmic tools to degrade over time if their development and validation are left to commercial entities with financial interests. For example:

*I think we need to really work on education of practitioners and our legal colleagues in terms of fundamentals of probabilistic [concepts], in terms of what it means to be transparent and to disclose limitations, and how we work with these kinds of new technologies (O#1).*

*We need to be realistic about how easy it is to implement them …. I think we need to think seriously about, given our movement toward these algorithms, the way we train forensic scientists and select them. So, picking people who have higher levels of mathematical and statistical aptitude training might be really important. At the same time, I think we need to be sensitive to current practitioners who are math phobic and, kind of ease them in and select more of those practitioners who have degrees in math and statistics, or the harder physical sciences and, thus, may be capable of moving into the new world with a greater degree of facility than we may see from the typical pattern matching person (O#2).*

## 4 Discussion

This study explored the perspectives of key criminal justice stakeholders, including laboratory managers, prosecuting attorneys, defense attorneys, judges, and other academic scientists and scholars on issues related to: (i) interpretation and reporting practices (with or without algorithmic tools) and (ii) the implications of the use of algorithms in legal settings as a means of calculating the probabilistic values assigned to the evidence. Participants offered a rich and diverse set of perspectives on these issues; however, we caution against generalizing these perspectives too broadly. We cannot suggest, nor do we believe, these perspectives are representative of the different stakeholder groups more broadly. Rather, we believe these perspectives are representative of a small sample of individuals that have been vocal and actively engaged in steering forensic science policy and practice over the last several years. Thus, while we must be careful not to over-generalize these individual viewpoints, we believe they provide valuable insights into the different perspectives affecting the current discourse in forensic science. Ultimately, we hope these insights provide a foundation for stakeholders to navigate a path forward that is cognizant and respectful of those different views, and generally amenable across all stakeholder groups. In the discussion that follows, we present a summary of the responses compared across the different stakeholder groups along with salient observations and key points of view related to these issues.

### 4.1 Interpretation & reporting practices

Participants offered different perspectives related to the validity and/or appropriateness of reporting results categorically versus probabilistically. Prosecutors expressed views that categorical reporting was most appropriate, with most participants citing the ease of understanding and one participant (P#3) citing the benefit of categorical reporting as the certainty it conveys to fact-finders. Defense attorneys, academic scholars, and judges, however, expressed views suggesting that the certainty it conveyed was the very issue of concern, that categorical reporting conveyed a degree of certainty that was outside the realm of what can be scientifically supported and, therefore, was unsubstantiated and inappropriate. Laboratory managers, on the other hand, were more ambivalent to the issue. While laboratory managers recognized the concerns that have been raised related to categorical reporting, specifically, the propensity for categorical reporting to mask the underlying uncertainty in the conclusion, they found categorical reporting acceptable if practitioners caveated the claims as their opinion.

Reporting results probabilistically, however, was not embraced *carte blanch*e by any stakeholder group. All stakeholder groups expressed concerns that probabilistic reporting would be confusing and easily misunderstood by lay fact-finders. While prosecutors expressed the greatest hesitation to probabilistic reporting, all other stakeholder groups expressed views suggesting that probabilistic reporting was superior, in theory, to the alternative (of categorical reporting as it is traditionally expressed); however, probabilistic reporting would need to be carefully implemented to ensure the uncertainties and limitations of such conclusions were appropriately conveyed. Among those stakeholder groups that were receptive to probabilistic reporting, defense attorneys were most concerned about the extent to which the conclusions would be empirically supported by validated statistical methods and the risks that probabilistic expressions would be misused by prosecutors to imply greater certainty than warranted. Judges questioned the extent to which lay fact-finders and other legal actors would be able to meaningfully scrutinize the validity of the underlying statistical methodology, but recognized its utility to cause people to pause and carefully think through what is being conveyed. Laboratory managers acknowledged the benefits of probabilistic expressions and numerical references to provide stronger foundations to expert opinions; however, they suggested probabilistic statements should not stand-alone. Academic scholars offered the least consistent views, with one scholar expressing strong views in favor of the transition to probabilistic reporting (O#1), another scholar expressing a more ambivalent perspective, suggesting there was no other better alternative (O#2), and the third scholar, aligning most closely with defense attorneys, pointing to the need for black-box testing to assess applicable error rates related to the performance of practitioners overall as the most immediate need.

Overall, all stakeholder groups viewed the benefits of categorical reporting as the clarity and simplicity to convey and understand such statements. These findings were not surprising and generally consistent with social science literature on lay understanding of statistical references (e.g., see Ref. [5]). Except for prosecutors, who did not express concern of any risks associated with categorical reporting, particularly under the auspice of an opinion, all other stakeholder groups suggested benefits were counterbalanced by the risk of making statements that were not scientifically valid or defensible (even under the auspice of an opinion) without some explanation around the uncertainties and limitations of the conclusion. On the other hand, most stakeholder groups viewed the benefits of probabilistic reporting as providing a means of conveying the uncertainties and limitations associated with the conclusion. However, all stakeholders noted that the extent to which those uncertainties and limitations are accurately represented depends on the extent to which such statements are based on empirical studies. None of the stakeholders expressed comfort with practitioners expressing conclusions probabilistically using numerical references without such numerical values being based on a validated statistical method. The chief concern being that the numerical values imply a level of precision and statistical basis to the assessment that cannot be substantiated. Thus, the so-called “subjective probabilities” approach, in which numerical values expressed are derived from subjective judgment rather than empirical measurement does not seem to be widely supported by stakeholders in the United States. That said, except for the majority of prosecutors, when sufficient statistical data is not available many of the other stakeholders responded in ways that suggested they were receptive to practitioners expressing conclusions probabilistically using qualitative statements without numerical references. These findings are generally consistent with guidelines set forth by the European Network of Forensic Science Institutes (ENFSI) in their Guidelines for Evaluative Reporting [52] as well as by the United Kingdom Forensic Science Regulator (UK FSR) in their Codes of Practice and Conduct: Development of Evaluative Opinions [53]. The ENFSI encourages numerical values be based on appropriate published statistical data, although as a “last resort” permits them to be based on subjective judgment [52]. The UK FSR, on the other hand, only permits numerical values be expressed if they are based on appropriate statistical data. In the absence of appropriate statistical data, the results shall still be expressed probabilistically but without numerical values [53]. Although the ENFSI and UK FSR advocate for likelihood ratios specifically, research has begun to explore how qualitative probabilistic statements should be phrased to ensure coherent interpretation by lay fact-finders (e.g., Refs. [54,55]). Overall, though, this approach seems to be generally acceptable as an alternative to categorical claims and intermediary until validated statistical methods become accessible.

When presented with the findings from a recent study characterizing practitioners' perspectives related to probabilistic reporting, which found that approximately 80% of practitioners cited concerns that probabilistic reporting would be confusing to lay people and would be misused by defense attorneys to create “reasonable doubt” [47], the different stakeholder groups had mixed reactions. On the former issue, nearly all participants across every stakeholder group agreed that probabilistic reporting would be more confusing to lay people and agreed practitioners should take this into account when debating ways to express their conclusions; however, none of the stakeholder groups suggested the confusion would be insurmountable or was sufficient of a reason to completely oppose probabilistic reporting altogether. On the other hand, to the latter issue, all of the stakeholder groups were critical that practitioners would bear such a concern. Some even suggested that's the very purpose of the legal system, for example, as noted by one judge, “that's what the system is all about, is finding out whether there is, or is not a reasonable doubt” (J#1), and as noted by one scholar, “creating reasonable doubt is what defense lawyers are supposed to be doing” (O#2). Some participants suggested this finding illustrates a cultural bias that is believed to underlie many forensic science disciplines. Others, particularly laboratory managers, while they did not personally support such a concern, recognized it to be an additional barrier that would need to be overcome if probabilistic reporting were to be adopted more widely by practitioners. How to overcome that concern, though, remains an open question. Separation of forensic science laboratories from law enforcement controls, as recommended by the NAS [1] could be a step toward mitigating such undercurrents, but greater understanding of the sources of such biases and the extent to which they can detract from sound scientific practices is needed.

One of the chief complaints with categorical reporting is that such expressions mask the uncertainties and limitations inherent in the interpretation of the evidence. Given the current discourse between categorical versus probabilistic reporting (e.g., Ref. [47]), we inquired what participants viewed as the roles and duties of forensic experts and the limits of their testimony. Admittedly, this question was attempting to elicit perspectives on a more technical nuanced issue, such as whether participants believed it was appropriate for experts to convey a statement about a proposition given a set of observations (i.e., a posterior probability about the source of an impression, or a decision that one proposition is true, such as “the two impressions were made by the same source”) or whether experts' testimony should be more limited to a statement about the observations given a set of propositions (e.g., the observations provide strong support for the proposition the two impressions were made by the same source, and weak support for the proposition the two impressions were made by different sources). However, given the intentional broadness of the question to be careful not to unintentionally steer participants toward a particular response, we found that participants incidentally offered a similarly broad response. Not surprisingly, the responses across every stakeholder group were generally consistent—participants repeatedly echoed the need for forensic experts to accurately and impartially convey their findings and limit the testimony to what is supported by the science, ensuring that the conclusions are neither overstated or understated. Not a single individual disagreed with this sentiment; however, what was most interesting is that there seems to be little agreement as whether practitioners are adequately fulfilling these duties and how they should be conveyed. Defense attorneys expressed very explicit frustration that practitioners rarely take these duties seriously and elaborate on the full scope of the limitations. For example, one defense attorney stated outright: “In my 20+ years of litigating many forensic cases, I have never encountered a forensic examiner who took this duty seriously. … It is always a game of hide and seek for examiners” (D#2). On the other hand, laboratory managers expressed the desire to be transparent about the limitations but expressed challenges in doing so most effectively, and also pointed to litigators and the courts as a factor that makes it even more challenging to convey these details during testimony. One academic stakeholder, however, suggested that the issue might be more deeply rooted in culture, commenting: “I think we just need to be so careful not to try and be so helpful to the court in helping them to get rid of the uncertainty that they don't like [such] that we stray beyond what we can robustly and scientifically say. It's something that I would say I've observed anecdotally over the years. … I think it's dangerous” (O#1).

Related to this issue of disclosing limitations of their examinations, when participants were asked whether they find it acceptable for experts to express their opinion in court without disclosing the underpinnings or statistical data to support those opinions, the responses were divided across stakeholder groups. Although all participants suggested it strengthens the testimony when experts explain the basis for their conclusions, prosecutors and laboratory managers did not believe it was necessary. Prosecutors pointed to their interpretation of statutory requirements as the guiding factor for their responses, and laboratory managers pointed to their past experiences. However, defense attorneys, academic scholars, and judges expressed counter views. Defense attorneys claimed such testimony would effectively be *ipse dixit* without such disclosure and is inadmissible under existing standards (despite courts allowing it in the past). Academic scholars recognized that different dynamics might affect testimony in a court setting but responded that such foundation was necessary from the perspective of sound scientific practice. Judges pointed to Daubert factors and prevailing admissibility standards suggesting such testimony should not be admissible; however, they recognized that many judges tend to admit it in anyways, referencing external pressures and past precedent. As one judge (J#3) explained, in general, “I think that some judges don't like to exclude. They'd rather let in than exclude and let it go to the jury. If there's an arguable basis for the jury to have accepted something, civil or criminal, then they [tend to] let it go to the jury. And that's a relatively safe place for them to be. If they exclude, they're subject to a reversal for an erroneous exclusion.” To illustrate this even further, another judge (J#1) pointed to the *United States v. Llera-Plaza* decision [56], where the judge after only two months reversed his earlier decision that fingerprint evidence did not meet the Daubert standard. For example:

*In his first opinion, [the judge] concluded [the fingerprint evidence] did not pass the Daubert standard. … In the second [opinion], Judge Pollack withdrew his earlier objections. I think frankly, under intense pressure, and that's not a good thing. I hope I'm not being unfair to judge Pollak, but not much had really changed between the first opinion [and the] second opinion. He said things in the second opinion, like, “well, I've learned since that it's accepted by the courts of Great Britain, and, so I'm going to accept it.” Who cares whether it's accepted by the courts of great Britain, they're not doing a Daubert analysis. The question is whether it meets Daubert or doesn't meet Daubert. So, I thought that was a cop-out and sort of revealing of the pressure he was under after his groundbreaking first opinion. [That aside,] I think that the evidence is there that fingerprint evidence is not junk science and that with proper limitations, it can be received in evidence (J#1).*

When asked what types of pressures might judges find themselves under, this judge offered an elaborated response pointing to political pressures, professional incentives, and biases to their own prosecutorial experiences. For example:

*I will speculate. I should tell you, though, the statistics are quite striking. Daubert challenges succeed in civil cases frequently. They succeed in criminal cases almost never. And that shows, I think, that there is a double standard operating. So, why is that? One factor is that in most states trial judges are elected, and if they have to face re-election on the basis they are “soft on crime” because by God, I wouldn't even allow fingerprint evidence in, they're in trouble to be re-elected or even to be renominated by the party of their choice. So, election is an element, but I think a more subtle element is going on in most of these cases. The stakes are so much higher and judges, having seen the other evidence in the case, may think “yea, he's probably guilty, but you never know what a jury is going to do. If I keep out this evidence, maybe there won't be a conviction, and I really think it would be unfair to the prosecutor not to at least be able to present this evidence to the jury and they can take it for what it's worth.” I think that is a wrongful attitude. I think I'd say a dereliction of duty and really ignores what Daubert is all about or even Frye for that matter. But, I do think that's a common traditional attitude: “I don't want to be responsible for this guy being acquitted, when, what I've heard so far, he's probably guilty.” And of course, forensic evidence carries great weight. It has an aura of neutrality that you don't have from testimony of accomplices, for example. So, I think judges are reluctant to keep it out. I'll mention a third factor, which is that most criminal court judges are former prosecutors. Relatively few are former defense lawyers. So, there's also, “oh yeah, of course. I always let this in, I used to do it myself. This is just routine. I recognize this” (J#1).*

This participant went further to provide another example and criticize a state Supreme Court decision in *Johnson v. Commonwealth* [57] in Kentucky that relied on judicial notice to admit microscopic hair analyses simply because it had been admitted previously without challenge. For example:

*Some courts, well, I will take the liberty of criticizing a court with apologies, which is the Supreme Court of Kentucky, which had for many years the Frye standard, then it adopted Daubert. Then the question came along, whether microscopic hair analysis met a Daubert challenge and a federal court in Oklahoma had already held that it did not. So, the defense lawyer in the case, Johnson v. Commonwealth* [57]*, a murder case, said we want to keep out this evidence, or at least we want a hearing, and the trial judge denied both and let in the evidence without a hearing. It went all the way up to the Supreme court of Kentucky, which held, with only one descending judge that, “well, all those years it came in under Frye and no one ever challenged it, so it must be good science.” I think that's bad logic. So, they went so far as to say that a court in Kentucky can take judicial notice of the fact that microscopic hair analysis is good science, which is a terrible decision (J#1).*

Finally, in wrapping up the broader topic of issues related to interpretation and reporting, we asked participants what they believed were the greatest challenges facing the pattern and impression evidence disciplines as it relates to examination and reporting methods. This question was intended to be a broad “catchall” question to allow participants to summarize what they believe to be the greatest need to support the pattern evidence disciplines moving forward. In response, participants pointed to a range of issues, often encompassing a scope much broader than just examination and reporting. Overall, however, defense attorneys, academic scholars, and judges all pointed to the need for more robust research establishing stronger empirical foundations and scientific rigor for many pattern evidence disciplines, including a better characterization of the limitations of those methods. Laboratory managers pointed to the need for additional resources to survive increasing caseload demands and to support foundational education and training needs for practitioners related to statistical issues and algorithmic tools that are being proposed. Prosecutors, on the other hand, pointed to partisan “attacks” from individuals or institutions attempting to undermine forensic evidence. Interestingly, this concern from prosecutors manifested throughout the interview and yielded an apparent contradiction. For instance, when responding to various questions throughout the interview, prosecutors were very deferential to scientists as to what they considered scientifically valid and appropriate as it relates to examination and reporting methods. For example, one prosecutor said quite explicitly:

*[W]hat drives my decisions here is what is legitimate science and what are the scientists saying? Not as much of what are the lawyers saying about it? What are the scientists saying about it” (P#2)?*

However, when prosecutors were asked if they were deferential to the scientists who have expressed concern over the validity and reliability of many forensic science methods, such as the President's Council of Advisors on Science and Technology (PCAST) [3], among others (e.g., Refs. [1,2,4]), prosecutors were quick to rebut the credibility of those reports and the individual authors. For example:

*… like the PCAST report, which, as you can probably guess, I think is not worth the paper it was written on” (P#1).*

*I found that virtually everything about that [PCAST] report was suspect. I don't have trouble with the statement that forensic scientists should be conservative and careful. … I think that seems self-evident, but if it was in the PCAST report, I don't think the report was honestly done (P#3).*

These views expressed from the litigators during the interviews, however, are not completely unexpected and are generally consistent with those that have been expressed by prosecutors and defense attorneys more broadly. Shortly after the PCAST report was published, it stimulated a flurry of responses from professional organizations involved in the criminal justice system. The National District Attorney's Association (NDAA), for example, published a response representing 2500 elected and appointed District Attorneys across the United States claiming “the NDAA takes issue with, and has substantial concern about, the logic of the [PCAST] report and the manner in which it portrays several forensic disciplines,” citing “the pervasive bias and lack of independence apparent throughout the report” [58]. Similar responses were made by other professional forensic science organizations, including the American Society of Crime Laboratory Directors (ASCLD) [59] and the Association of Firearms and Toolmark Examiners (AFTE) [60], among others, which disagreed with several of the conclusions issued by the PCAST. Defense attorneys, on the other hand, welcomed the report with open arms; for example, the National Association of Criminal Defense Lawyers (NACDL) stated the report “offers further evidence of the pervasive use of flawed analysis erroneously presented as grounded in science” [61], and the Innocence Project claimed the report “provided a blueprint for fixing one of the most critical problems plaguing the criminal justice system” [62].

This sharp contrast between prosecutors and defense attorneys is not only evident from the published responses but has also been noted through anecdotal observations by academic scholars who have looked into the forensic sciences from neutral, outside perspectives. For example, one scholar, (O#1), commented during the interview about this “ongoing narrative of forensic science in crisis.” This participant, (O#1), lamented that such narrative is “unhelpful,” and forensic science “can still be of assistance to the courts, …but we must be honest about its limitations” (O#1). Another scholar, (O#3), noted “it's principally in the prosecuting community that I see the most resistance. … I understand why, I understand what you're saying, but you're not being completely honest with the jury if you say that” (O#3). When asked why this participant, (O#3), considers prosecutors as the most resistant, the participant simply pointed to the adversarial nature of the criminal justice system. For example:

*What you have is a back and forth between two sides, presenting evidence. The point of the exercise is to convince the majority of the triers of fact that my side has done better on the argument than yours. So, if you have a tool in that process of back and forth that lends more credence to the points that we're making than the other side, then you're not going to want to give that tool up. The way that forensic science is currently structured, mostly that tool is something that prosecuting attorneys can use (O#3).*

Related, during the interview, when one judge, (J#1), was asked their view on a comment made by one prosecutor, (P#3), that they view the greatest challenge facing the pattern evidence disciplines as “trying not to fold in the face of that kind of pressure [from people] attempting to appease the defense bar,” (P#3), this judge, (J#1), responded by elaborating on the nature of the adversarial system and the emotion that runs high in the criminal justice system which only exacerbates such contrast between prosecutors and defense attorneys. For example, participant (J#1) stated:

*Well, I don't know [that prosecutor] means other than [the prosecutor] thinks [they are] always right, and those defense counsel exercising the right of the defendant under the Constitution of the United States are evil people who are trying to pervert justice. But, it is of course true that every prosecutor has sooner or later a case in which they think a guilty person was wrongfully acquitted, and the nature of the adversary system, unfortunately, is you always impute the worst motives to your opponent. So, even in civil litigation, I'm confronted repeatedly, “judge, you won't believe what that guy on the other side did! It's outrageous! It's immoral! It's illegal! It's wrong!,” and, usually it's some little squabble over nothing, but the emotions grab you. So, when the stakes are as high as they are in criminal cases, the emotions run even higher and you are very quick to impute to your adversary, “[they] only won that case cause [they] pulled the wool over the jury's eyes or whatever. One great privilege I've had as a judge is to talk to the jurors after each case, and I've had more than three hundred jury trials. There are some civil, but more criminal, and I am constantly impressed by how carefully juries take their obligation. They know the stakes in criminal cases are real and they take it very seriously. When I asked them, “well, how did you arrive at that decision?” They almost always give me good reasons. Occasionally I'll disagree with them. Like most judges, I'm more inclined to convict than to acquit if I were on the jury, but it's not because they're not giving me good reasons for the acquittal. And of course, acquittals are still a tiny, tiny fraction of the cases. To me, what should be bothering the prosecutors is the now indisputable proof that the system sometimes convicts innocent people. And who's responsible for that, Mr./Ms. Prosecutor, if not you? So, the very familiar word of Justice Jackson, when he was Attorney General of the United States, I still think should ring in every prosecutor's ear, which is [paraphrasing] “your job is to do justice, not to convict, not to exercise hunches, but to make sure that you have analyzed every case carefully and then go forward if you can objectively say that you have proved beyond a reasonable doubt. Not to view it as this is a competition, a game, an adversary process.” Hard to avoid that in an adversary system, but I still think that's the right attitude for a careful prosecutor (J#1).*

From these results we see that the pattern evidence disciplines are facing a myriad of perspectives from various stakeholders across the criminal justice system as they relate to interpretation and reporting methods in the pattern evidence disciplines. Overall, it appears that prosecutors' perspectives represent one extreme end of a spectrum and defense attorneys' perspectives represent the other extreme, particularly as they relate to the validity and appropriateness of traditional practices. Broadly speaking, prosecutors expressed the desire for practitioners to adhere to good scientific practices and argue that existing methods are appropriate. On the other hand, defense attorneys argue that existing methods go beyond the standards of good scientific practice, are invalid, and are inappropriate. Of course, this opposition is not completely unexpected given the adversarial nature of the American legal system. Responses from laboratory managers, judges, and academic scholars seemed to be less extreme, but still represented an affinity toward one side of the spectrum compared to the other. Perspectives from laboratory managers tended to align more closely with prosecutors in the sense that they maintained perspectives that traditional practices were acceptable (although maybe not ideal); however, judges and academic scholars tended to align more closely with defense attorneys in the sense that they were more overt with their concerns as it relates to traditional practices. Comparing these results to those of pattern evidence practitioners (e.g., see Ref. [47]), we would conclude that practitioners’ perspectives align most closely with laboratory managers. For example, from Ref. [47], we see most practitioners tend to maintain the perspective that traditional reporting methods using a categorical framework are appropriate and defensible, although there is a growing minority that believe probabilistic methods are a more suitable alternative. All stakeholders, however, including practitioners (i.e., see Ref. [47]), expressed concern that probabilistic reporting methods will bring new challenges that have yet to be fully explored.

Despite the nature of the discourse and diverse perspectives on this broader issue of interpretation and reporting, it seems that there were some areas in which stakeholders offered shared perspectives, particularly in relation to the benefits and limitations/risks of categorical reporting versus probabilistic reporting. Nearly every stakeholder recognized the need for forensic conclusions to be scientifically defensible *and* easily interpretable. The major critique of categorical reporting is that it is not scientifically defensible (at least, how it is traditionally expressed), but it is easily interpretable. The major critique of probabilistic reporting is that it can be more scientifically defensible^10^; but it is not as easily interpretable. Although we recognize that no single approach will satisfy all stakeholders, perhaps an immediate next step for the community to consider is a combination of the two. Admittedly, given the discourse on this subject to date, going into the interviews we held a belief that probabilistic reporting was going to be considered the ideal by many stakeholders, particularly defense attorneys. Although most stakeholders did express the superiority of probabilistic reporting over categorical reporting, the responses left us skeptical as to whether the superiority was truly because of the benefits of probabilistic on its own, or merely because it was the better of the two without any other alternative when presented in a binary context. Recognizing that defense attorneys represent an extreme end of the spectrum in terms of the various perspectives, suggesting traditional categorical claims were inappropriate, interestingly, they did not seem to wholly endorse probabilistic reporting outright as the preferred alternative. Instead, defense attorneys were most concerned about ensuring the limitations of the methods (and all sources thereto^11^) are clearly explained. In that sense, probabilistic expressions that account for the strength of evidence are relevant, but information related to empirical measures of error rates^12^ were equally, if not more, important in their view.

#### 4.1.1 Use of algorithms

Participants offered generally consistent perspectives related to the use of algorithms in court and the benefits and limitations of them. All stakeholders were receptive, at least in theory, to the use of algorithms, and pointed to several benefits algorithms could provide, such as better means of reflecting the strength of evidence, promoting greater objectivity and consistency in examination results, and enabling examinations to be performed more efficiently. Stakeholders differed, however, as to how they viewed the limitations of algorithms. Prosecutors, while generally receptive to algorithms, questioned whether they were truly necessary compared to traditional methods in pattern evidence disciplines (versus their necessity for DNA interpretation, for example). Prosecutors seemed to be most concerned whether algorithms would unduly complicate reporting and testimony, making it more difficult for lay-fact finders to understand the testimony. Laboratory managers, defense attorneys, academic scholars, and judges, on the other hand, were most concerned about the transparency surrounding these systems, the underlying validity of the systems, and the risks of analysts and lay fact-finders blindly relying on the output of algorithmic tools without fully understanding and accounting for their limitations. Laboratory managers were concerned about delegating decision-making responsibilities from the analyst to the algorithm, suggesting that algorithms would be most useful as tools to supplement their judgment rather than supplant their judgment. Defense attorneys were most frustrated about the lack of access and proprietary protections that have been placed around algorithms in the past preventing their disclosure of the underlying source code. Academic scholars were most receptive to algorithms provided they were sufficiently validated but speculated about whether algorithms can be thoroughly validated such that all limitations and boundary conditions are known, particularly as the algorithms become more complicated and less transparent. Judges were most concerned about ensuring algorithms were trustworthy, reliable, and fair, and that defendants are afforded the opportunity to challenge the evidence against them and exercise their due process rights granted under the Constitution.

A salient theme in the conversation about algorithms was how they could be trusted for use in court thereby having an impact on human liberties. All stakeholders suggested that trust requires that the algorithm be validated, and validation requires that the algorithm be shown to be “reliable” through performance testing. Academic scholars, defense attorneys, and judges, however, suggested that trust also requires that the algorithm be shown to be “fair,” which may not necessarily be determined through performance testing alone. These stakeholders, in addition to some laboratory managers, went a step further and pointed out more nuanced details that are required, in their views, for an algorithm to be appropriately validated. The design and conceptual operations of the algorithm must also be understood to ensure that the validation testing is appropriately designed and that the boundary conditions for which the algorithm is able to appropriately function can be established, such that the conditions for which the algorithm is expected to work well and the circumstances, or combination of circumstances, for which the algorithm begins to fail are known. Defense attorneys, judges, and academic scholars all noted that these details lie within the source code. Academic scholars recognized the value of source code but held back from suggesting source code was the only means by which that information could be ascertained. Defense attorneys and judges, on the other hand, argued that the source code and the software application containing the algorithm were critical to permit an independent evaluation and testing under conditions they consider appropriate given the circumstances of the case at hand. Consequently, these stakeholders expressed strong views that disclosure of source code is necessary to enable criminal defendants to mount an adequate defense, and failure to provide access to these materials could be considered an infringement of the defendant's Constitutional right to due process. As one judge (J#3) commented: “When we're dealing with due process and equal protection under the United States Constitution, we are now in a world where ‘the means to the end’ matter, [and] the means are contained within the source code.” Concerns about the capacity to meaningfully scrutinize algorithmic tools in the absence of source code and its impact on criminal defendants' Constitutional rights is not limited to these participants. It is a perspective that is held more generally (e.g., see Refs. [34,35,37,38]). Although prosecutors, laboratory managers, and academic scholars did not express such a strong view on the necessity for disclosure of source code as defense attorneys and judges did, all stakeholders suggested they would be amenable to the disclosure of the source code if desired by the defendants and they were in a position to disclose it.

The issue with disclosure, however, is that some commercial vendors of algorithms have exerted trade secret protections to prevent such disclosure, and some courts have therefore been faced with balancing these countervailing positions. When stakeholders were asked how courts should address these issues, nearly every single participant pointed specifically to protective orders or described a level of protection that is comparable to a protective order. Many stakeholders, particularly prosecutors, defense attorneys, and judges, were quick to dismiss trade secret protections as even being an issue given that there are existing mechanisms for protecting intellectual property, but also suggested the ideal situation is that these algorithms are open and publicly accessible without the need for such court orders, given that they are being used for human liberty decisions. Defense attorneys and judges both pointed to civil litigation as examples of established precedent and procedures for how to permit disclosure while still protecting intellectual property concerns from commercial vendors. Given these perspectives, when judges were asked why courts have failed to mandate disclosure, some judges were openly critical of those rulings. One judge (J#3) went so far as to claim they were “wrongly decided,” for example:

*I think they're wrongly decided …. We do know how it's done, and there's a whole body of case law that can be utilized from the civil side and transferred over. So, it is possible that what we're seeing is just a lack of experience by some of the state court criminal judges with the disclosure of the super-secret stuff. The federal judges ought to know how it's done, because we did it all the time, and they do it all over (J#3).*

Interestingly, one defense attorney (D#3) suggested that prosecutors should not proffer evidence from an algorithmic tool unless they can disclose the source code under discovery. When presented with this perspective, prosecutors claimed that if they did have access to the source code, then it would be disclosed under existing discovery rules. However, they often do not have access to it but also don't believe it is critical to possess before proffering such evidence. When asked how prosecutors can assure the evidence they are proffering from an algorithmic tool is trustworthy without having access to the source code, the prosecutors often pointed to the forensic scientists for such assurances based on their validation testing. When laboratory managers were asked whether disclosure of source code was a factor that they considered when procuring an algorithmic tool, they all claimed it would be taken into account, but was not a governing factor in the decision. Laboratory managers tended to be more focused on the performance characteristics and capabilities offered by the algorithmic tool versus issues related to disclosure but recognized the benefits of procuring an algorithm for which the vendor was willing to disclose the source code when requested.

Having discussed what stakeholders considered were necessary for algorithms to be trusted and the role of source code in that assessment, stakeholders were asked their opinion about the use of algorithms based on AI/ML methods, which are often “black boxes,” even to their developers, and that human interpretable source code is effectively nonexistent. While the specific responses varied between individuals both within and between stakeholder groups, most individuals across all stakeholder groups expressed even more caution and skepticism with the use of AI/ML algorithms compared to their existing concerns related to “traditional” algorithms based on straight programming and rule-based approaches (i.e., non-AI/ML-based algorithms). Although most individuals were receptive to the idea of using AI/ML algorithms in theory, participants from each stakeholder group expressed a number of concerns about their transparency and whether they can be sufficiently tested such that the boundary conditions are known to permit an appropriate validation for practical application. Laboratory managers recognized the additional complexity of these types of algorithms but seemed to be the most receptive to their use provided they were sufficiently validated and demonstrated superior performance characteristics. Academic scholars were the most concerned about the practicality of performing all the testing that would be necessary in order to fully trust the algorithm and whether such testing was practical, for example, as one scholar commented: “I don't think the idea is actually practical [and] realizable. I don't think you could actually implement it” (O#3). Prosecutors, defense attorneys, and judges expressed an additional layer of caution with reference to potential concerns on a Constitutional dimension, such as whether the application of these algorithms could be an infringement on due process and confrontation rights. Ultimately, these stakeholders suggested that the admissibility of these types of algorithms would require careful consideration on a case-by-case basis depending on what information was available about the algorithm, such as design, inputs, parameters, weightings, training data, validation data, etc., and how those details relate to the circumstances in the case at hand. The various responses from these participants across all stakeholder groups, and general hesitation concerning the use of AI/ML algorithms overall, illustrate that many of these issues have yet to be fully fleshed out. Indeed, it is a novel subject that legal scholars are just beginning to discuss (e.g., see Ref. [38]). For example, as one judge (J#3) made clear:

*What we need is a national conversation on what that means and how to create trustworthy and reliable algorithms that can be used for individual liberty determinations. That's where the rubber meets the road. … The greatest risk is that we allow complex design and complex tools to just snow us a little bit … [and] that we don't have these conversations as to what fairness means and what fair design is and what trustworthiness is in time (J#3).*

Given the concerns that have been expressed about the use of algorithms, participants were asked their opinion about whether they should be regulated, and, if so, how. This question stimulated several diverse responses, and some stakeholders took this opportunity to express their views on the regulation of forensic science more broadly. Prosecutors and laboratory managers tended to be deferential to the forensic science community to establish applicable guidelines in a centralized and coordinated fashion, but then leave it to the legal community to enforce those guidelines, where appropriate, on a case-by-case basis. These participants seemed to be generally satisfied with how the legal system has regulated forensic science practices more broadly and believed the legal system would be similarly effective with the regulation of algorithms. Defense attorneys, academic scholars, and judges, however, rejected the idea that the legal system could be effective at regulating algorithms. For example, these participants went so far as to claim the legal system “has proven to be an utter failure” (D#2) and “defective” (J#1) in its ability to regulate forensic science more broadly. Instead, these participants suggested algorithms, and forensic science overall, should be regulated by an independent entity with both oversight responsibilities and approval authorities. Participants, however, were not as aligned on whether the entity should be part of the federal government. Academic scholars also pointed out that such regulation should address the entire method rather than just the algorithm itself (e.g., inputs, personnel, testimony, etc. associated with the use of the algorithm). The perspectives from defense attorneys, academic scholars, and judges are not limited to just these individual participants; instead, they generally align with one of the key recommendations from the 2009 NAS report [1], which is to create an independent Institute of Forensic Sciences staffed by high level scientists, which one judge (J#1) lamented never received enough traction to materialize. Although academic scholars recognized the OSAC as a step in the right direction, there were mixed perspectives as to whether the OSAC is able to provide the central coordination desired by some or assess the appropriateness and rigor behind the validation of forensic methods. To illustrate the concerns related to the topic of regulation more broadly and to highlight the need for greater consistency across the forensic science community as it relates to resources and practices, when one academic scholar (O#3) was asked whether they trust forensic science overall today based on what they have observed as an “outside scientist” over the last several years, they responded:

*It depends on where I am. Literally. It literally depends on my physical location, because if I'm in a location where I have some confidence that the forensic scientists are appropriately supported, with the proper amount of resources to perform at the highest level,yes, I would trust them. [But,] if I'm someplace where that's not the condition, [then] no,I'mnotgoing to trust them (O#3).*

Finally, in wrapping up this broader topic related to the use of algorithms, we asked participants what they believed were the greatest challenges facing the operational use of computational algorithms for court purposes. This question was intended to be a broad “catchall” question to allow participants to summarize what they believe to be the greatest need to support the pattern evidence disciplines as algorithms become more available. In response, participants pointed to a range of issues, often encompassing a scope much broader than just the use of algorithms. Overall, all stakeholder groups (except for judges)^13^ pointed to the need for greater investments in foundational education and training for the forensic science and legal communities—specifically practitioners who will be expected to use the algorithms and judges who will be expected to assess the admissibility of the algorithms. Prosecutors also pointed out the need for ensuring the algorithms are developed in a way that can be effectively explained in lay terms to fact-finders, recognizing that the more complicated computational methods become, the more challenging it is to present scientific evidence in court. Laboratory managers expanded on the need for better training and echoed their prior concerns related to lack of resources to support the validation and implementation into day-to-day practice. Academic scholars also pointed to the need to be clear about what type of algorithms are being considered (i.e., traditional rule-based programmed algorithm vs. AI/ML-based algorithm) so that stakeholders have a common understanding of the varying benefits and risks surrounding their use, the need to consider changing recruitment and selection of forensic practitioners to those who have higher aptitudes in physical sciences and mathematics, and the need for safeguards, standards, and oversight to be placed around the use of commercially developed algorithms to prevent financial interests from impacting the quality of their development and validation.

From these results we see that the use of computational algorithms in court is a complicated issue. While all stakeholders across the criminal justice system were welcoming of the potential benefits that algorithms can provide, they all expressed caution about the risks associated with them and the need to carefully consider the more nuanced details around their development and implementation—the central issue being how algorithmic tools can be trusted for court purposes that can directly impact human liberty decisions. We find that trust is a complicated and multi-dimensional concept, and stakeholders have similar but inconsistent and incomplete perspectives on what that entails. Overall, stakeholders held a variety of perspectives on these issues related to the use of algorithms, but all expressed a shared desire to ensure these systems are developed and implemented in a responsible and practical manner that upholds the values of fairness and equal justice under the law. How this can be done in a structured and consistent way requires a broader national dialogue. In recent work, we have begun to explore this in greater detail based on perspectives that have been raised in the literature thus far and provide some initial recommendations [41]. The perspectives expressed in the present study provide greater breadth and depth to these issues. While we believe they align with those that have been raised thus far in the literature, this study reinforces the need for this conversation to occur sooner rather than later. It will only be a matter of time until these algorithmic tools are introduced for court purposes, and it is critical that we have a shared perspective and mutually agreeable framework for how to address these issues before we find ourselves in a legal quandary.

It should also be noted that participants' experiences related to algorithms were widely variable. Although all participants had direct knowledge and experience dealing with algorithms in the criminal justice system in one capacity or another (e.g., related to their use, development, validation, or litigation), many of the questions related to this issue required participants to speculate in general terms without a single specific algorithm to point to, and were focused on the use of algorithms for court purposes which have direct impacts on decisions impacting human liberty. Some participants noted a distinction with the use of algorithms for other purposes in the criminal justice system, such as for investigatory leads or general purposes to augment traditional policing practices, and recognized that their perspectives might vary as it relates to algorithms designed for those purposes, since their benefits and risks can be very different. Although issues concerning the use of these types of algorithms were outside the scope of this evaluation, it is relevant to note that some stakeholders suggested the risks associated with the use of algorithms for those purposes can be much lower compared to the risks associated with the use of algorithms for human liberty decisions. Other stakeholders, particularly defense attorneys, however, asserted that algorithms used for investigatory purposes should be held to the same standards as algorithms intended for court. Although this perspective was not broadly shared across other stakeholder groups, the primary concern expressed from defense attorneys is that these types of algorithms eventually make their way into court and once they do, they can significantly influence fact-finders’ decisions that impact human liberty. Considering the nuances that often impact stakeholders’ perspectives on these issues, additional research is needed to explore the implications of algorithms used for purposes other than court, such as investigatory purposes, to better understand whether, and under what circumstances, they could be used that are generally amenable across stakeholders.

## 5 Conclusion

Over the last decade, there have been increasing calls for the introduction of probabilistic reasoning and validated statistical methods into forensic practice—particularly in the pattern evidence disciplines—to formally recognize and articulate the uncertainties inherent in forensic interpretation and reduce the heavy reliance on subjective judgment. While probabilistic reasoning can be achieved without the need for sophisticated technology, computational algorithms are often a means by which empirical measurements are made and probabilistic values are assigned to the evidence. In recent years, various approaches have been proposed. However, reactions to probabilistic reporting and the use of computational algorithms in forensic science have been mixed. Some commentators have argued that probabilistic reporting and computational algorithms promote more scientifically defensible reports and provide more objective and greater scientific capabilities to the evaluation of forensic evidence. Others, however, have argued probabilistic approaches unduly complicate the issue, and the opacity of algorithmic tools makes it challenging to meaningfully scrutinize the evidence. Consequently, the forensic community has been left with no clear path forward on how to navigate these mounting concerns as each proposed solution seemingly has countervailing benefits and risks. In order to better understand these issues, this study elicited the perspectives of key criminal justice stakeholders, including forensic laboratory managers, prosecuting attorneys, defense attorneys, judges, and other academic scientists and scholars on issues related to (i) interpretation and reporting practices (with or without algorithmic tools) and (ii) the implications of the use of computational algorithms as a means of calculating the probabilistic values assigned to forensic science evidence in the American legal system. This study was conducted as one-on-one semi-structured interviews of fifteen individuals (three from each stakeholder group) resulting in over 20 h of recorded interviews and over three hundred pages of written transcripts capturing their perspectives on these issues. Although the number of individuals from each stakeholder group prevents broad generalizations, these individuals are considered prominent in their fields and have various marks of distinction, such as occupying senior level roles in their disciplines, served on boards and committees steering policy and practice recommendations, and are influential in the practices of others across the broader community, either directly through supervision or indirectly through training and continuing education activities. Participants’ responses were rich with information illustrating their diverse viewpoints on various issues and providing valuable insights into the different perspectives affecting the current discourse in forensic science.

As it relates to interpretation and reporting practices, we found that the pattern evidence disciplines are facing a complex myriad of perspectives that has effectively stifled the ability to find consensus on nearly every issue. Generally speaking, prosecutors' perspectives often represented one extreme end of a spectrum and defense attorneys’ perspectives represented the other extreme. Perspectives from laboratory managers tended to align more closely with prosecutors in the sense that they maintained perspectives that traditional practices were acceptable (although maybe not ideal); however, judges and academic scholars tended to align more closely with defense attorneys in the sense that they were more critical and expressive of their concerns as it relates to traditional practices. Nearly every stakeholder recognized the need for forensic conclusions to be scientifically defensible *and* easily interpretable. However, stakeholders differed on how that should be accomplished. Further, although stakeholders generally agreed on the roles and responsibilities of experts and the importance of ensuring opinions expressed during testimony are accompanied by the underpinnings or statistical data to support those opinions, they differed in their views related to whether forensic practitioners are adequately fulfilling those roles and responsibilities and whether disclosing that information is necessary from scientific and legal perspectives.

As it relates to the topic of the use of computational algorithms in court, we found that stakeholders recognize their potential benefits and, in theory, were receptive to their use. Generally, stakeholders pointed to the benefits algorithms provide as being a better means of reflecting the strength of evidence, promoting greater objectivity and consistency in examination results, and enabling examinations to be performed more efficiently. Stakeholders differed, however, how they viewed the limitations of algorithms. Prosecutors seemed to be most concerned whether algorithms would unduly complicate reporting and testimony making it more difficult for lay-fact finders to understand the testimony. Defense attorneys, judges, academic scholars, and laboratory managers, on the other hand, were most concerned about the transparency surrounding these systems, how to ensure the underlying validity of the systems, and the risks of analysts and lay fact-finders blindly relying on the output of algorithmic tools without fully understanding and accounting for their limitations. These concerns highlight the need to carefully consider the more nuanced details around their development and implementation—the central issue being how algorithmic tools can be trusted for court purposes that can directly impact human liberty decisions. However, we find that trust is a complicated and multi-dimensional concept, and stakeholders have similar but inconsistent and incomplete perspectives on what that entails. Overall, despite stakeholders having a variety of perspectives on these issues related to the use of algorithms, they all expressed a shared desire to ensure these systems are developed and implemented in a responsible and practical manner that upholds the values of fairness and equal justice under the law. How this can be done in a structured and consistent way requires a broader national dialogue to occur sooner rather than later. In our view, computational algorithms are now beginning to be introduced for court purposes, and it is critical that we have a shared perspective and mutually agreeable framework for how to address these issues before we find ourselves in a legal quandary.

Looking forward, participants pointed to several challenges facing the forensic science community. First and foremost, there is a need for more robust research establishing stronger empirical foundations and scientific rigor for many pattern evidence disciplines, including a better characterization of the limitations of those methods. As this research develops, and computational algorithms become more accessible, however, the challenges will become even more complex. As we consider the use of computational algorithms, we need to be sensitive to the diverse perspectives related to their use from different stakeholders operating within the criminal justice system. Overarching all else, there is a need for greater investments in foundational education and training for the forensic science and legal communities—specifically practitioners who will be expected to use the algorithms and judges who will be expected to assess the admissibility of the algorithms, as well as greater allocation of resources for forensic laboratories to support these investments while maintaining the caseload and throughput demanded of them. Second, we need to be conscientious that these algorithms need to be understandable and explainable to lay fact-finders, recognizing that the more complicated computational methods become, the more challenging it is to present scientific evidence in court, and that starts with how the algorithms are designed and developed. Third, we need to be clear about what type of algorithms are being considered (i.e., traditional rule-based programmed algorithm vs. AI/ML-based algorithm) so that stakeholders have a common understanding of the varying benefits and risks surrounding their use. Fourth, we need to consider changing recruitment and selection of forensic practitioners to those who have higher aptitudes in physical sciences and mathematics. Finally, we need for policy-safeguards, standards, and oversight to be placed around the development, validation, and application of forensic science methods, including algorithmic tools. Overall, these growing concerns and diverse perspectives illustrate a need for additional research and a national conversation to continue across the criminal justice community on how to navigate a path forward most effectively in a manner that is both cognizant and respectful of the different views and generally amenable across all stakeholder groups. Until that occurs, we can expect growing divisiveness and continued frustration amongst different stakeholders as we seek a more effective administration of justice.

## Declaration of competing interest

The authors declare that they have no known competing financial interests or personal relationships that could have appeared to influence the work reported in this paper.

## Footnotes

1 The term “computational algorithms” refers to automated or semi-automated computer implementable processes designed to compute mathematical outputs for purposes such as forecasting, predictions, statistical evaluations and decision making. For purposes of this paper, the term “algorithm” and “computational algorithm” are synonymous. The term “algorithmic tools” refer to devices enabling the applications of computational algorithms.

2 This participant, (J#2), was first appointed by the state Governor in 2015 to fill a vacancy and elected to start a new term in 2016.

3 The majority of the interview with this participant, (J#3), focused on issues related to the broader topic of “computational algorithms” for court purposes. Many questions related to the broader topic of “interpretation and reporting” were omitted and, therefore, are not discussed.

4 Participant (J#3) did not provide a specific perspective on issues related to this topic.

5 Participant (J#3) did not provide a specific perspective on issues related to this topic.

6 Participant (J#3) did not provide a specific perspective on issues related to this topic.

7 Participant (J#3) did not provide a specific perspective on issues related to this topic.

8 This participant, (O#1), was the only non-U.S. centric participant.

9 This participant noted the awkwardness of the question to suggest the doubt be “reasonable.” The wording of the question was intentional and correctly represented how it was phrased in the survey to practitioners—as “reasonable doubt.” See Ref. [47] for the wording of the question as phrased to practitioners.

10 Provided the basis for the probabilistic statement is disclosed (e.g., human judgment versus statistical methods), along with those limitations and numerical references are empirically demonstrable.

11 In this sense, we distinguish between the uncertainty of an association based on a coincidental match and the error rate based on the performance of the expert.

12 Error rates in this context refer to blind performance testing of examiners under normal casework conditions.

13 Due to time limitations, judges (J#1, J#2, and J#3) did not provide a specific response to this question.
