# Supplementary material for: Probabilistic reporting and algorithms in forensic science: Stakeholder perspectives within the American criminal justice system
Source: Forensic Sci Int Synerg. 2022 Feb 12;4:100220. doi: 10.1016/j.fsisyn.2022.100220 (PMC8850671; doi:10.1016/j.fsisyn.2022.100220)
Supplement: Multimedia component 1 [file mmc1.pdf]

Appendix 1

PARTICIPANT INVITATION LETTER

Dear Participant,

I am a doctoral candidate pursuing a degree in forensic science through the University of Lausanne, Switzerland, under the direction of Dr. Christophe Champod, Professor of Law, Criminal Science and Public Administration. The focus of my research is on the development and implementation of computational algorithms for forensic science. As part of this research, I am conducting a study to explore the perspectives of criminal justice stakeholders (laboratory managers, prosecuting attorneys, defense attorneys, judges, and other stakeholders [e.g., academic scholars]) as it relates to issues concerning the use of probabilistic reporting (with or without algorithmic tools) and the use of computational algorithms in forensic science for court purposes.

I am writing to invite you to participate in this study and contribute to this broader effort. Our interest in this topic is broad and includes technical, operational, and legal dimensions. Our ultimate objective is to characterize various stakeholder perspectives on these issues to enable a path forward for the forensic science community as it relates to the use of probabilistic reporting and computational algorithms in forensic science. We aim to enroll approximately fifteen participants (three from each stakeholder group). This study will be conducted as a semi-structured interview of each participant lasting approximately one-hour. Identities of participants will be kept confidential and not publicly disclosed. Participation is by invitation only and selections are based on participants having been actively engaged in issues concerning forensic science policies, procedures, and practices.

For your convenience, I have attached the following items:

(1) Participant information and consent form
(2) A short description of the purpose and background of the study
(3) A one-page guide outlining the structure and questions that will guide the interview
(4) My curriculum vitae outlining my professional background and experiences related to issues concerning forensic science

I hope you will accept my invitation to participate. Please let me know if I can answer any questions or be responsive to any concerns you might have.

NOTE: Although my academic pursuits are through the University of Lausanne, I am physically located in Washington, D.C. and the research is focused on issues concerning forensic science practices in the United States.
