# Supplementary material for: Probabilistic reporting and algorithms in forensic science: Stakeholder perspectives within the American criminal justice system
Source: Forensic Sci Int Synerg. 2022 Feb 12;4:100220. doi: 10.1016/j.fsisyn.2022.100220 (PMC8850671; doi:10.1016/j.fsisyn.2022.100220)
Supplement: Multimedia component 2 [file mmc2.pdf]

Appendix II

PARTICIPANT INFORMATION AND CONSENT FORM

Title and Summary of the research

Probabilistic Reporting and Algorithms in Forensic Science: Stakeholder Perspectives within the American Criminal Justice System

Over the last decade, with increasing scientific scrutiny on forensic examination and reporting practices, there have been several efforts to introduce probabilistic reasoning and computational methods (i.e., algorithms) into forensic practice. Although various approaches have been proposed, reactions to probabilistic reporting and the use of algorithms in forensic science have been mixed. This research is aimed at exploring the perspectives of key criminal justice stakeholders (laboratory managers, prosecuting attorneys, defense attorneys, judges, and other stakeholders [e.g., academic scholars]) to improve our understanding of the issues related to the use of probabilistic reporting practices (with or without algorithmic tools) and the use of algorithms in forensic science for court purposes.

The current study is conducted by Henry Swofford, under the supervision of Dr. Christophe Champod, Professor at the School of Criminal Justice, University of Lausanne, Switzerland.

A) PARTICIPANT INFORMATION

1. Objective of the Study

This study focuses on the issues concerning the use of probabilistic reporting and computational algorithms in forensic science for court purposes by exploring the perspectives from various key stakeholders in the criminal justice system (e.g., laboratory managers, prosecuting attorneys, defense attorneys, judges, and other academic scholars). Our interest in this topic is broad and includes technical, operational, and legal dimensions. Our ultimate objective is to characterize various stakeholder perspectives on these issues to enable a path forward for the forensic science community as it relates to the use of probabilistic reporting and computational algorithms in forensic science.

2. Procedure

If you agree to take part in this study, you will be asked to participate in a semi-structured interview lasting approximately one hour, at a time of your convenience using a virtual meeting platform.

This study aims to enroll approximately fifteen participants (three from each stakeholder group). Participation is by invitation only and selections are based on participants having been actively engaged in issues concerning forensic science policies, procedures, and practices.

3. **Confidentiality**
   The interview will be digitally recorded to facilitate subsequent analysis. The information gathered will be kept strictly confidential and will be destroyed at the end of the study. You will be assigned a unique identifier within your respective stakeholder group; however, your personal identity will not be disclosed or publicly attributed to any specific statements.

4. **Voluntary Participation/Withdrawal from the Study**
   Your decision to participate in this study is entirely voluntary and no compensation will be provided. You are free to withdraw at any time by simple verbal notice. Any personal data gathered prior to your withdrawal will be destroyed.

5. **Conflict of Interest**
   No one on the study team has a financial interest related to this research.

B) STATEMENT OF CONSENT

1. **Declaration by Participant**

   I have read the preceding information thoroughly. I have had the opportunity to ask questions, and all of my questions have been answered to my satisfaction. I understand the purpose as well as the nature of the study.

   I hereby consent to take part in this study:

   Participant's Name:____

   Signature:______________________________Date:____________

2. **Declaration by Researcher**

   I have given a verbal explanation of the research project to the participant, and have answered the participant's questions.

   Researcher's Name:____

   Signature:______________________________Date:____________

C) CONTACT

For additional information about the research, please contact Henry Swofford at henry.swofford@unil.ch or Dr. Christophe Champod at christophe.champod@unil.ch.
