# Supplementary material for: Probabilistic reporting and algorithms in forensic science: Stakeholder perspectives within the American criminal justice system
Source: Forensic Sci Int Synerg. 2022 Feb 12;4:100220. doi: 10.1016/j.fsisyn.2022.100220 (PMC8850671; doi:10.1016/j.fsisyn.2022.100220)
Supplement: Multimedia component 3 [file mmc3.pdf]

Appendix III

STUDY PURPOSE AND BACKGROUND

Title and Abstract of the research

Probabilistic Reporting and Algorithms in Forensic Science: Stakeholder Perspectives within the American Criminal Justice System

Over the last decade, with increasing scientific scrutiny on forensic examination and reporting practices, there have been several efforts to introduce probabilistic reasoning and computational methods (i.e., algorithms) into forensic practice. Although various approaches have been proposed, reactions to probabilistic reporting and the use of algorithms in forensic science have been mixed. This research is aimed at exploring the perspectives of key criminal justice stakeholders (laboratory managers, prosecuting attorneys, defense attorneys, judges, and other stakeholders [e.g., academic scholars]) to improve our understanding of the issues related to the use of probabilistic reporting practices (with or without algorithmic tools) and the use of algorithms in forensic science for court purposes.

The current study is conducted by Henry Swofford, under the supervision of Dr. Christophe Champod, Professor at the School of Criminal Justice, University of Lausanne, Switzerland.

Purpose:

To explore perspectives from key criminal justice stakeholders related to interpretation and reporting practices (with or without algorithmic tools) and the use of computational algorithms in forensic science for court purposes.

Background:

For purposes of this study, we focus on the use broad use of computational algorithms in traditional forensic science disciplines (e.g., DNA, facial recognition, fingerprints, footwear, firearms, handwriting, etc.) for court purposes. Computational algorithms used outside of traditional forensic science disciplines or for non-court purposes, such as for investigatory or intelligence purposes, are outside the scope of this study.

For purposes of this study, the following terms and definitions apply:

- Appropriateness: the quality of being suitable or proper in the circumstances.

- Validity: the quality of being logically or factually sound. E.g., the extent to which a conclusion is logically justified and factually supported by the measurements resulting from the examination performed.

- Categorical (reporting): The expression of results in a manner which does not formally recognize or articulate the uncertainties inherent in the interpretation or the possibility for an alternative proposition to be true. NOTE: categorical reporting includes unadorned statements of “match,” “identification,” or “individualization” without articulation of an associated error rate, statements that attribute two samples as being made by the same source, and statements that encourage uncertainties to be disregarded (e.g., “practical impossibility,” “negligible,” “discounted”).

- Probabilistic (reporting): The expression of results in a manner which formally recognizes and articulates the uncertainties inherent in the interpretation and the possibility for an alternative proposition to be true. NOTE: probabilistic reporting includes statements that are expressed as a likelihood ratio, posterior probability (of a proposition), match probability, or any other expression which is accompanied by an explicit statement of recognizing the possibility for an alternative proposition to be true.

- Computational Algorithm: A broad term to describe any computer and mathematically-based prediction method, such as statistical models or other defined sets of computer implementable mechanical processes used for forecasting, predictions, statistical evaluations and decision making. NOTE: computational algorithms include human-interpretable rules or processes as well as non-human interpretable processes, such as those developed through artificial intelligence and machine learning (AI/ML).

For purposes of this study, the following terms are intentionally not defined to ensure we do not limit participants’ responses: “benefit(s),” “limitation(s),” “risk(s),” and “trust.”
