# Supplementary figures and images for: Probabilistic reporting and algorithms in forensic science: Stakeholder perspectives within the American criminal justice system
Source: Forensic Sci Int Synerg. 2022 Feb 12;4:100220. doi: 10.1016/j.fsisyn.2022.100220 (PMC8850671; doi:10.1016/j.fsisyn.2022.100220)

## Appendix IV

### INTERVIEW GUIDE

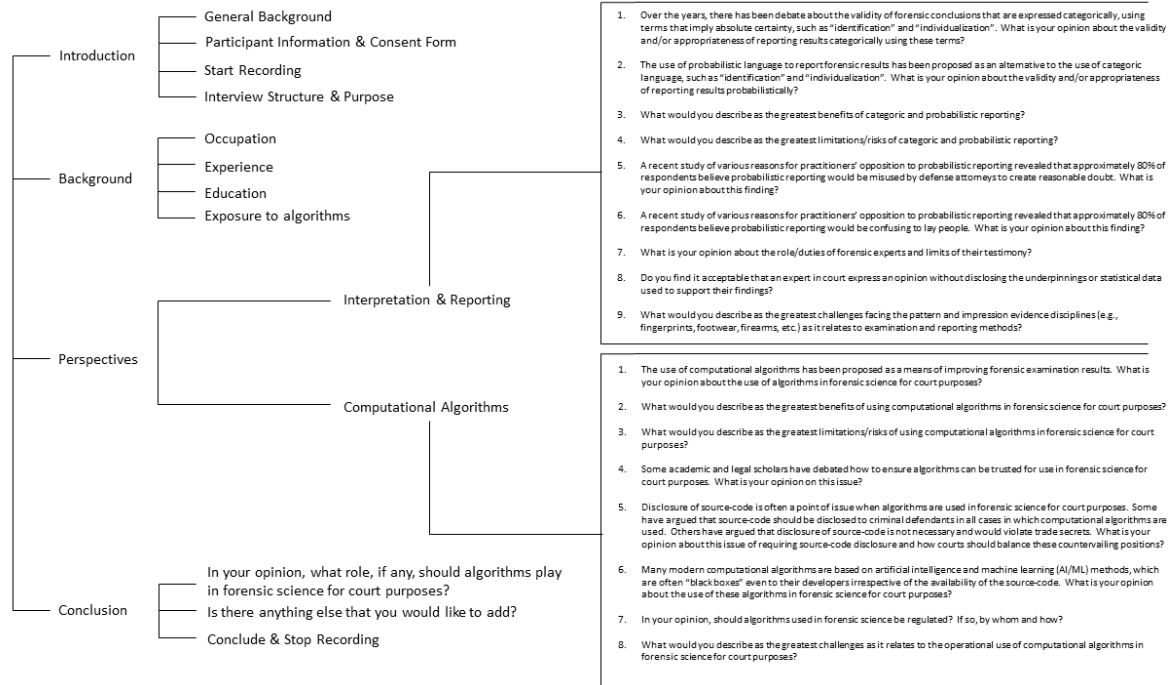

Supplement: Multimedia component 4 [file mmc4.pdf]
