# Supplementary material for: Probabilistic reporting and algorithms in forensic science: Stakeholder perspectives within the American criminal justice system
Source: Forensic Sci Int Synerg. 2022 Feb 12;4:100220. doi: 10.1016/j.fsisyn.2022.100220 (PMC8850671; doi:10.1016/j.fsisyn.2022.100220)
Supplement: Multimedia component 5 [file mmc5.pdf]

Appendix V

PARTICIPANTS' ELABORATED RESPONSES RELATED TO INTERPRETATION AND REPORTING PRACTICES

Participants' responses related to the validity and/or appropriateness of reporting results categorically:

Laboratory Managers

> *I know there's been a lot of controversy on [expressing conclusions categorically]. ... And I do understand there's comparisons where people have maybe overstated their data, a bite mark, smudged fingerprints, [for example; however,] if I've got a broken taillight with tremendous detail where I can fit it back together, I'm not doing anybody a service of saying "it could have come from there or really hedging my opinion when anyone, even a layman, can look at it and go, 'that came from there'" ... it is my opinion. Even though it's considered maybe by some, a little taboo, it's still individualizing. There are still cases where that's the case. ... [but] I do believe people are overgeneralizing [to lump all comparisons together] (LM#1).*

> *Nothing's absolute. I get the drive of why those answers came to be, [such as] "beyond a scientific certainty," or whatever other dumb language gets used. The reality is nothing, nothing is absolute. Everything has some level of uncertainty to it. So fundamentally, I guess, I disagree with the kind of categorical absolute statements. It's just not the case. That being said, I do understand how that gets driven there because the question in court is "how certain are you?" Well, how do you put some kind of framework around that? And I think every discipline actually struggles with that. ... Not to jump off into anything too entirely philosophical [but] there is uncertainty in the entire system. I think it is an inherent obligation to try and express the limitation of the result. And that limitation can take a lot of different forms, but that you have at least attempted to express the limitation of it. ... I've read through a number of [pattern evidence examiners'] testimonies ... [and] they say, "my opinion is," not "the result is" or "these two are a match," but [they say] "in my opinion ... this is my opinion." They have been very careful to qualify it as an opinion. I can live with that because they are the instruments and their assessment of these images is their trained opinion. It can be a very weighty opinion, but given their opinion how do you provide any kind of uncertainty on how good is their opinion? Well, blind testing starts to give you a bit [of insight] (LM#2).*

> *Absolutes and conclusions, I think, are probably inappropriate. I, however, do not have a problem with experts giving their opinion. I think we have very good experts. I think expertise matters. I think exposure to casework matters. I do agree with a lot of the defense experts and the academics that we need a reasonably good way to express uncertainty. ... It's a little fuzzy right now [of how to express that uncertainty, but] this is the nature of black box studies, [as well as] ... talking about the person's experience, talking about the number of samples this person has looked at, or the number of positives*

they've made, the number of exclusions they've actually made, the amount of training [they have had], the breadth [of their training]. ... The black box studies, I think, are an attempt to provide numerical, objective measures of those conclusions that experts actually draw. ... It's really a holistic approach, not just an absolute number that you get with digital data (LM#3).

Prosecutors

I don't think saying identification implies absolute certainty. And, individualization, I don't think that to a layman that implies absolute certainty. ... And I know that in the past, specifically in latent prints, and then also I believe in firearms and tool marks, there would be additional certainty statements beyond that, like, to the exclusion of all other firearms in the world, or a hundred percent certainty. I agree that those types of statements should not be used, those outside certainty statements. But I actually think that the categorical approach and pattern evidence, at least based on what I've seen so far, is probably the most appropriate, scientifically appropriate, and also easily understandable way of reporting conclusions to both litigants and ultimately the fact-finder, be it a judge or a jury (P#1).

I'm not sure that is the case. I think there's a debate that's going on in the forensic community and certainly amongst some of the legal community about whether those terms imply [absolute certainty]. I don't think that they imply that. ... Now there's going to be limitations to what they can also say related to that identification. And I think that's well put by the Department of Justice Uniform Language with Testimony and Reports. ... But the statement itself, that this is this expert's opinion, that this is an identification. I have no pause about that (P#2).

I don't have a problem with the use of a categorical response. It's easy to understand. It's easy for the jury to grasp, and I believe that it is the true opinion of the scientist who's giving us that opinion. I think that the backing away from the certainty and the different locutions that have been proposed, I think those are imposed on the analysts or the scientist. ... I think that given their own druthers, they would say forthrightly, it's a match. And I don't think they would say it's a match if they didn't believe it was a match (P#3).

Defense Attorneys

I think it's particularly problematic to be offering something like an individualization conclusion. ... My personal opinion is that fingerprints probably are unique, but that's not a scientific validated fact, and we shouldn't express those opinions in court as though they were. I think what's probably more important is that we don't know how similar prints can be and in latent print examination we are dealing with very small amounts of data. ... I don't think it ever should be that person to the exclusion of every other human ever born on the planet. You know, it's funny that virtually anybody will agree with it when it's couched like that in this hyperbolic language. But the reality is that's virtually every opinion that you ever get introduced in court is essentially that. ... If you're going to

make an association at all, it should never be categorical, and the association should always allow for the possibility of error or the possibility of a random match (D#1).

These terms as they have been used by forensic examiners have always been misleading and they have resulted in systematic overstatement of the forensic evidence in ways that have been detrimental to people charged with crimes all over the country and in a way that most in the forensic science community have failed to come to grips with even today. … The desire to cling to claims of certainty have been so overwhelming and blinding that it largely took scientists and other concerned parties from outside of the forensic science community to tell forensic examiners to stop claiming such certainty (D#2).

There's a tremendous amount of concern. Specifically, because there's essentially no scientific foundation for the claims of identification that are being made in almost all of the pattern disciplines. We've been objecting to that for many, many years and trying to explain to the courts why the validation studies that are put forward in some of these disciplines don't support the statements that are being made (D#3).

<u>Judges</u>

As I think many people know, bad forensic science has been an element in the conviction of innocent people. ... One of the reasons that those inaccuracies [in forensic science] came about [was] because the science itself was much more subjective than was represented to courts and to juries, [and] because they were presented as being certain conclusions. [For example,] in many states to this day, forensic scientists cannot testify unless he or she is prepared to state that they have reached their conclusion to "a reasonable degree of scientific certainty." Now, if you look at the case law, the case law often says that the courts regard that as nothing more than more probable than not—very far from certain—but what the jury is hearing and what judges who are not familiar with this case [law] are hearing is "scientific certainty." There's almost no part of science that can claim certainty. If you talk to physicists or chemists or whatever, they won't claim that. Yet here it is, in effect, being claimed by forensic science. ... [Although experts are allowed to express their opinion,] in the federal system, it's not good enough for someone to say, this is what I believe. They have to show that it has a scientific basis through a Daubert analysis. ... [Among several other factors] you have to show that it doesn't have a significant error rate. This is where a lot of forensic science fails. You have to show that the methodology used is reliable so that it could be used again and again, with the same results and with two different examiners coming up with the same results. That often is not correct or not true in certain types of forensic science. ... [This is applicable] [i]f it's being offered as science. Now, if it's not offered as science, all those requirements still apply, but they are applied more flexibly (J#1).

I think it's very challenging to use [categorical statements] for purposes of how to report a result. Part of that is because I don't know that they're particularly well-defined and my experience seems to be that attempts to define those categorical statements do not always align with what a lay person's understanding of the definitions would be. ... So, from that standpoint, I do think that it can be challenging. ... [Further,] as far as an expert

testifying based on the "training and experience" [and] "to a reasonable degree of blank certainty," whether that's "ballistic certainty" or whatever, I think that has no place in a courtroom, period. I don't think it has a definition. ... But, as far as source attribution itself, ... as long as [the examiners] can back it up, especially if, as they explain[ed] their analysis, they explained the things about their opinion that have to do with the uncertainty so that they're saying the parameters and limitations of what it is that [they] can tell you, [then] that that's fair game. ... [But] do we really know what ground truth is and how do we get there? How do I know that there's the foundational science to be able to say that, as we're doing this comparison, that I can make the statement, "yes, this impression came from this source?" We get into [things] like, "well, it's a match." Well, okay. It may be, [but] how do you know that (J#2)?

<u>Other (Academic Scholars)</u>

I would rather see a complete move away from using those terms. Accepting that that move takes time, in the interim I think there [should] be a clear explanation given alongside the terms, that they are only opinions and that two examiners wouldn't necessarily come to the same opinion in a more complex case (O#1).

I think it's clearly not justified scientifically. It's an overstatement of the value of the evidence. We know it's simply not plausible for a discipline, like fingerprinting, that a trained examiner can determine the rarity of the set of features observed [based solely on human judgment] with the precision necessary to know whether it's probability in the population is low enough to support the claim that it's a unique observation. From my standpoint, the psychology of human judgment, it's totally implausible that anybody could make that kind of judgment. ... It's not just unjustified, from my point of view it's a prosperous claim. It's a laughable claim. ... Even then, black box studies show the error rates are low, but not so low as to justify a claim of identification, so it's empirically debunked too. ... [Further,] Daubert [admissibility standard] doesn't or shouldn't allow you to get away with saying, "well, there's no scientific basis for this opinion, but it's my opinion anyway, so I'm going to say it." ... From my point of view, forensic scientists should not be claiming they can identify things because it's a clear overstatement of the value of the evidence. It's a claim that they can do something that they clearly cannot do. It claims too much. They need to acknowledge the potential for error and uncertainty. ... Even couching it as an opinion should not be allowed. I guess the question is do the legal standards for admissibility of evidence, such as Daubert, allow experts to present something which does not have an adequate scientific basis so long as the experts say, "but it's just my opinion." Answer: No! Saying "it's just my opinion" sort of softens the claim a little bit, [but] I think we'd be much better off if we had some algorithm that would put a weight or strength of evidence statistic on it, even if it supported claims far less extreme (O#2).

I think it is wrong. I think it's immoral to stand in front of a jury and make categorical statements if you are a forensic scientist because the word "scientist" confers in the minds of the jury that you are, well, one way that I heard it expressed is that the words have totemic power. I think it's wrong to abuse that level of trust. ... I'm all for making

sure that statements in front of the jury, in front of any trier of facts, when presented as forensic science must be accompanied by how certain I am based on the scientific principle about the statement. ... If someone is an expert then you can give an opinion, but if you claim your opinion is based in science, then that constrains you to give a scientific opinion. ... To say that I have absolute certainty on the basis of my experience, you have gone right outside of the role of science. ... Look, the way I view it, we can make categorical statements, but don't claim it's backed up by science (O#3).

Participants' responses related to the validity and/or appropriateness of reporting results probabilistically:

Laboratory Managers

I like [numbers] because it provides [context]. On the other hand, even numbers have their limitations. ... How do you throw somebody just a number and expect them to understand it? ... It's still not standalone. You just don't lob the grenade of the number in [the courtroom] and run out. You have to explain it. ... The danger is people just start to rely on a number, [but] what does that mean? That's where the expert [comes in and explains] what this means. ... Our job is as witnesses, as opinion witnesses, as expert witnesses, is to provide that interpretation. ... So, [the] danger is you let [numbers] do too much of the talking, but it's tremendously good when you use it to inform your opinion. And, that's part of the whole package. It's better with it than without it, if done correctly (LM#1).

From a philosophical standpoint, I think it is more appropriate. What I see though, is a hell of a lot of confusion on the part of the lay person and lawyers and juries. There is a seductive, unfortunate, seductive nature to it that it seems like it should be an appropriate way to express things that allow the fact-finder to understand how reasonable it is to make their decision on those data. The reality is as humans, we suck at basic risk assessment, and you are giving people a number to base against their personal experience and their biases to assess risk. So, I guess it's better than categorical, but it has its own pitfalls (LM#2).

I don't have a problem with [probabilistic reporting] per se. ... I think some standardization in the industry is warranted and good for juries so that when one person says it versus another person, we're at least talking on the same playing field rather than completely different contexts. Some of the terminology is difficult for juries to understand. ... I think we have to be concerned about how juries are going to take the information. I think this is why a forensic scientist as an expert is so important because they bring the human element to interpretation and explanation that I think is so critical. ... [That said,] I struggle a little bit with reporting a number unless you have a numerical basis for it. ... I have no problem with subjective interpretations [such as] "in my experiences," [or] "is very likely," just as a subjective conclusion, but if you're going to put a number on it, I think you need to have some basis [of] where you're pulling the number from (LM#3).

Prosecutors

So obviously probabilistic language has been used in reporting DNA results forever. ... I don't have any information or knowledge as to how something similar would be done in a pattern discipline. ... I would be open to considering it ... but I think that the analyst's report or the scientist giving their conclusion, about whether, for example, a particular tool could be the source of a particular tool mark is sufficient. ... [However,] ... prior to trial, the risks are the reader is going to misunderstand the value of the opinion. I think that risk exists, whether you use categorical language, or whether you use more likelihood kind of language in a report. And actually, I would say in my experience, the more complicated the written opinion in the report gets, the more likely that law enforcement and prosecutors, at least at first blush, are going to be confused by it without calling and having a conversation with the analyst. ... [Ultimately,] ... if the experts themselves decide that articulating their opinions in that manner is more scientifically accurate than in, let's say a categorical kind of statement, if they decide that they think that that more correctly states their scientific opinion, then I would be fine with that (P#1).

A probabilistic conclusion is a lot looser and as a result is much less clear what that means. So, when you say more likely, what does that really mean? More likely to whom? To you? To me? To the juror? What if we have different standards about what's more likely and what's less likely? What's negligible to me may not be negligible to you. So, I think we all have very different standards there and that looseness, I think gets us into muddy waters, and can cause more problems for both defense and for prosecution because both sides may manipulate that language in a way that's unfair since it's looser (P#2).

I think both of the statistics that we use in DNA, the RMP, which, describes the rarity of a profile in the population or a likelihood ratio, which pits two hypotheses against each other and tells you which one is more probable given the evidence. I think both of those are basically easy to explain to a jury and easy to explain to prosecutors and the court and it was relatively easy for me to understand, and I have no science background. ... But I don't see where the numbers come from in pattern matching. ... I just don't see how that's possible. ... Or where even if you're not using a numeric value, if you're using a verbal scale, I just don't know where that data comes from. ... I think it's less precise and I think it's actually way more subjective because, you know, strong support, very strong support, ... what does that mean? I think it means different things to different people and numbers mean different things to different people. ... So, I think it's problematic (P#3).

<u>Defense Attorneys</u>

I think as we sit here today, when those types of conclusions are offered at trial, there's no real empirical support for that. ... What I think is that there would be appropriate black box testing that shows that with the quality of evidence at issue in a particular case with the same examiner or an examiner of similar level of experience and expertise that those examiners get the "right" answer X percentage of the time, and given that, in these circumstances, I would expect to get the appropriate conclusion x percentage of the time.

And I think that as we sit here today, that's about what is the most scientifically valid statement that you can make. ... In the meantime, if we can develop a fingerprint database, we can do the statistical work that needs to be done and develop some empirical evidence for the uniqueness or the rarity of particular types of prints and we can have some objective thresholds onto what is an appropriate print for analysis at all, then I would feel a lot more comfortable to talk about what kind of numbers that we were going to be offering for probabilistic testimony in latent print analysis (D#1).

I think the move towards probabilistic language for any forensic discipline that doesn't have reliable rarity data is really problematic. ... The other problem with that is that I know fingerprint examiners won't know what source of error is included in that statement and what sources of error are not included in that statement. They will make that statement without either recognizing or admitting to the other sources of error that could adjust that. ... If you gave me the choice between probabilistic reporting or categorical reporting accompanied by accurate statements about the weaknesses and limitations of the forensic method, I would take the latter every time (D#2).

As a litigator, my primary concern is what the jury does with the testimony that they receive. There's a significant concern that jurors, number one, don't really understand probabilistic language and that prosecutors will misuse it. ... The second concern is, I think there is also a dearth of validation demonstrating the validity of probabilistic language being used, either in numerical form or by verbal texts. ... At the end of the day, if there were studies to support that type of language, and if there was some way to ensure that jurors understood what it meant and it was not misstated by either the examiner or by the prosecutor, I think probabilistic language is probably preferable. ... I think probabilistic language better conveys the limitations to a jury, which is obviously essential and necessary. Whether it's reliably and accurately presenting the limitations, I think lies in the validation (D#3).

Judges

Well, I think [probabilistic statements] would be an improvement, but I worry again about two things. First, the ability of judges and juries to really scrutinize, in a meaningful way, when someone says it's this probability or that probability. And secondly, the validity of the underlying statistical methodology used, which varies considerably. ... [Additionally,] most forensic science involves a high degree of subjectivity. I don't think you can easily translate that into statistical probabilities. [What that means is that] there is not only the possibility of human error, but that there will be a considerable range between good practitioners and bad practitioners. So, it's not a question of overall statistical probability. Nevertheless, I think expressing it as a probability would still be better than expressing it as a certainty. But I do think it still has a great potential to confuse (J#1).

Probabilistic [approaches], I think, provides us with an overall framework that [can] cross disciplines and allows us to be able to talk about how we are actually putting a statistical model on something to give it value. At first, when I started working with

them, I was like, this is way too confusing and there's no way we're going to be able to do this in a way that's meaningful to people, but in some ways, I think there are some things about it that makes it more approachable. ... Probabilistic and mathematical statements having to do with the probability that one source is from the other provide a different layer of meaning, which, although far more confusing in many ways, it seems like once you start working with them, [they are] almost easier to define. [For example,] if I'm a juror, and I hear the word match and that word has been defined within this particular community as meaning whatever it's defined as, but I hear it as "they're the same and you know they're the same, like two pairs of socks match." I know what match means [colloquially] so I may not listen to the nuances. ... [That said,] I think that if you're going to use statistical modeling and probabilistics, you should be using numbers. If you want to assign a category, like a word to a category of numbers to make something easier, fine, but I think you have to be able to have some kind of modeling that you can do to be able to get to that answer (J#2).

<u>Other (Academic Scholars)</u>

I strongly believe that [probabilistic reporting] is the appropriate approach to take. I also firmly believe that we must have more effort to improve the data sets that we have available to us to make that probabilistic approach more robust. But even in the interim, where we don't have necessarily the best datasets, it's still better to use a logically robust framework and be open about the lack of data in some cases. I really think that we need to push forward both with the probabilistic approach and in parallel with the generation of data sets to help us with that probabilistic interpretation. ... It is much more scientifically correct and defensible to acknowledge that uncertainty in a probabilistic form (O#1).

I really don't see any way to avoid probabilistic presentations because there's not a scientific justification for categorical determinations. We live in a probabilistic world, so if we're going to be honest about it, the source of our knowledge that we gain as forensic scientists, we have to acknowledge the probabilistic nature of it. ... From my point of view, we basically are at a point where we have to do it and the real discussion should be what's the best way of doing it. I'd say, knowing what's the best way of doing it is an issue on which more research is needed. ... [As for the basis for examiners' conclusions,] I think there are a lot of situations where we just will need to continue relying on examiners' judgment. I do think that examiners' experience gives them some basis for making judgments. There's a whole literature on the accuracy of human judgments and particularly people's abilities to estimate the rarity of frequency of events. [However,] we know that people tend to be overconfident in their ability to do this kind of thing, so we know experts are going to tend to think they can do more than they can do, but that's not the same thing as saying that they can't do it. So, do we allow that person to present their results in court? I've sort of come around on this and said, "okay, well, I think that we probably should allow them to say something about it, but we need to be very careful about what we allow them to say because of this overconfidence problem." ... I would allow experts to give testimony in these areas just because I think it can sometimes be useful and maybe necessary, so I think it's evidence that we want to have in criminal

cases because I do think these experts know more than non-experts about lots of things, but I would really hem them in, and it needs to be transparent and the uncertainty needs to be fully acknowledged (O#2).

I have problems with [probabilistic reporting] too, but the problems don't lie on the side of the forensic science community, it lies on the side of the triers of fact. [For example,] I know for a fact, most people don't understand fractions ... I'm not sure why a statement of probabilistic interpretation of the data taken at a crime scene actually is better than making a categorical statement. To me, [it doesn't] pay deference to the capacity of the trier of fact to integrate properly [the] information. So, I'm not sure if probabilistic is better, but I know a lot of people are in favor [of it]. I don't think that's a great improvement. ... What I love to see is a commitment to study [and] education. ... I'm willing to give [forensic examiners] the benefit of the doubt [as to their expertise] as we perhaps move to a more efficient system, but the thing that I would also say is "let's test it." We should be testing this. I think the expertise is actually there, but I would love to be able to have a scientific test where I could stand up for my scientific colleagues to say, "we know that these people, although you don't understand how they're arriving at these right answers, that test showed that they can do what they say they can." ... To me, the most useful thing we could do while we're kind of waiting for the scientific foundations is to simply ask the question, "how accurate is the system performing right now?" It's a measure that will give me some comfort and say, "this is the best we can do in the current situation." ... [Ultimately,] if you can tell me how often you make a mistake, then that's something that a trier of fact can get their hands on and say that the scientific evidence tells us you make a mistake one in every 100 times or even one in a million times. That gives me confidence and trust [in] the other things that you're saying (O#3).

Participants' responses related to the benefits and limitations/risks of reporting results categorically and probabilistically:

<u>Laboratory Managers</u>

N/A

<u>Prosecutors</u>

When thinking about probabilistic reporting, especially when numbers were applied in statistics, it goes back to if you have a likelihood ratio, let's say the likelihood ratio considering this evidence is 10,000 times more likely to be this defendant versus some unknown person. Let's say that that's the conclusion. 10,000 means what to me? And what does it mean to you and what does it mean to a juror? And even sliding scales that give you that qualitative statement about the strength of that support? What is that based on? So, I think it gets messier the more you start complicating the conclusions in pattern matching disciplines (P#2).

The benefit for categorical is the certainty of the opinion. And that's one piece of evidence that the jury has. ... If you're not using numbers the way we do in DNA, then I think the weakness is that the descriptors are built on sand. I think there's way less foundation for those kinds of answers than for a categorical answer (P#3).

<u>Defense Attorneys</u>

I don't think either approach is more beneficial, absent accurate statements of the limitations and weaknesses of the method. ... If with a fingerprint probabilistic reporting scheme, large-scale studies showed that examiner agreement with each other a high percentage of the time when selecting probabilistic reporting language, and further showed that the language selected was an accurate statement of the value of the evidence, and further showed the jurors understood all of this accurately, we could then be in a position to discuss benefits. But any discussion of benefits in the absence of that research is nonsense (D#2).

The positive is that [categorical statements] are easy to understand. It's very clear what an examiner is saying, and from the perspective of a litigator and a jury, I would think that categorical statements are much easier for them to understand. But it doesn't really accurately convey the weight of the evidence. ... I think very clearly categorical statements overstate the evidence, and that is always a significant danger. I think that's the biggest problem. ... [On the other hand,] I think probabilistic statements they more accurately convey the weight of the evidence, [but] I think they are very difficult for judges, juries and litigators to understand. ... The prosecutor's fallacy was always a real problem for us when we were dealing with DNA litigation and that was, in my view, a simpler concept to convey and the prosecutors consistently got it wrong (D#3).

<u>Judges</u>

N/A

<u>Other (Academic Scholars)</u>

The greatest benefit of categorical reporting is [its] simplicity and ease of understanding, perhaps ease of people thinking they understand anyway. It's a very easy concept for a lay person to say "this expert thinks these two are from the same source." ... [However, the] greatest limitation [of categorical reporting] is that it doesn't acknowledge or is transparent about the fact that it isn't a black and white decision. ... Another weakness is that it doesn't allow the expert to give any indication to the court about what value there may be [for] that great big number [of comparisons] in the middle that are inconclusive [e.g., whether those comparisons support exclusion, or support inclusion]. [On the other hand,] when it comes to probabilistic methods, you're not trying to force a continuum into boxes with artificial distinction. You're not trying to make something that is all the way from white to black through gray, into white, black, or "I don't know," and you're able to give more information about more samples with more transparency. ... [However,] the greatest disadvantage [of probabilistic reporting] is that we have few

well-developed, deployable, validated methods to help practitioners to deploy those sorts of methods. Although I'm a strong believer in the likelihood ratio is the appropriate framework for reporting of evidence, my gut feel is that it is misunderstood widely by lay people and that lay people routinely transpose [the] conditional. So, if I had one biggest fear, it's that (O#1).

[Categorical reporting] is cut and dry—perfectly clear. It's easy for the jury to understand that the expert is telling them "it's him." It's easy to understand. The cognitive demands are low. [However,] from a scientific perspective, these [categorical statements] are problematic claims. So, do we allow experts to say things that are not scientifically justified because they're really, really easy to understand? Well, I don't think so. In fact, if that's your strongest argument, you're on shaky ground. ... I think this argument that we should continue making unjustifiable claims because they're easy to understand just becomes laughable. I mean, you're violating the prime directive, which is be a scientist. ... [On the other hand, probabilistic reporting] acknowledges the reality that our evidence is probabilistic. If we're going to be honest about the nature of the evidence that forensic scientists can offer, we're inherently going to be in a probabilistic world. ... [However, probabilistic reporting is] difficult to understand and interpret properly. It takes people outside of their common experience and comfort zone to be dealing with numbers of this sort and there are known biases and misinterpretations that people are susceptible to. So, it has to be presented with care and, I don't think there's a clear indication in the literature about the best way to do that yet. ... [Overall,] I think the standards that we're developing for presentation should require careful acknowledgements of uncertainty and transparency about where the numbers are coming from. So, I think given that we're in this uncertain period where we don't know, we don't have a consensus on the best way to present things, let's be really open, honest, and maybe a little conservative about how we approach it (O#2).

Participants' responses related to findings from a recent survey of forensic friction ridge practitioners indicating 80% of respondents believe probabilistic reporting would be confusing to lay people:

<u>Laboratory Managers</u>

I understand the concerns. It doesn't help anybody if that is not understood, but when you think about what our job is, in the shortest term, [it is to] maximize the value of evidence. If you have this car that is faster, quicker, does things. It could be more dangerous, [but] you still can't not use it. It's a better car, learn to drive it, apply the tools (LM#1).

Watching what I've seen happened with biology, yes, it will be confusing. Is it irrevocably confusing? No. I think everybody in the system can learn how to deal with it and how to explain it. We've been through multiple iterations already of refining how we explain it. You can see the growth curve in the lawyers that are involved. You can see the growth curve in the courts. Obviously, people may serve on one jury in their lifetime. It's not like they're going to come back and have learned something from the last time they were on a

jury, but people get better over time about being able to explain it. That window of that confusion narrows down. So yes, I agree, the lay people will be confused. The practitioners are confused by it right now. But that is (1) not a reason to not go there, and (2) not an indelible absolute. The confusion will subside. The confusion will abate and people will get better about explaining it (LM#2).

It probably will be. This is why I don't want it to be only probabilistic reporting. I think the type of testimony that we're currently giving plus this is the best model for the future. I think as it's used more and as we can standardize some things it will be easier because attorneys will know how to ask it and it will be used in a more standard fashion. So, it will be very frustrating, just like likelihood ratios still are right now, with juries (LM#3).

<u>Prosecutors</u>

I think that they should be worried about it to a certain extent. They should be cognizant of whether what they are saying at trial is an accurate description of their opinion (P#1).

There've been cases where prosecutors misunderstood DNA results in a case where there wasn't a match and inappropriately argued those results to juries and those cases were reversed. So, a lot of this is on the part of the scientists and the prosecutor to foundationally understand what the conclusion is, and the limitations of the conclusion are appropriately presented. When both of those things happen, I'm less concerned that a jury is going to misunderstand it (P#2).

<u>Defense Attorneys</u>

[My opinion is] [t]hat 80% of latent fingerprint examiners find it confusing (D#1).

It's clear that people struggle with probabilistic notions. Most of the studies are pretty clear on this. People don't know what's encompassed in a random match probability. People have illogical uses of probabilistic statements as well as verbal expressions of probabilistic statements and sometimes do use those in incongruent ways. So yes, I agree (D#2).

I actually tend to agree with that. Until there is a lot of effort put into: number one, doing the underlying validation, and then number two: figuring out how this is presented and how it's made clear, that we are not saying that X matches Y. That is a real danger. ... I actually do think that the forensic science community does have some obligation for thinking through how information should be accurately reporting. I actually do think it is within their purview because I think that, again, that's something that for years has not been, either intentional or unintentional, but there have been overstatements made in every discipline for years and years and years. So, I think it's important for them to understand that they need to convey information accurately and clearly (D#3).

<u>Judges</u>

Well, I do think there is a potential for confusion, but it's not as bad as the view that the jury will take otherwise, that it's an absolute fact. When the jury hears the opinion it's a match, their natural reaction is to say, "okay, it's been scientifically found that it's a match. Period." When they hear that, "well, there's an error rate of [X]%." Yeah, that may be hard for them to fully digest, but at least it is better than the clearly erroneous view that they are otherwise presented with that it's a hundred percent and no error or zero error rate and a hundred percent correct. I do think, if I had a magic wand, everyone would take a course in statistics in first year of college because it permeates so much of modern life. But, there's no reason why a good expert, on either side, you can't give the jury the basics of statistics so that they can appreciate what those statistics mean. I also think, even more globally, even jurors or judges without that background know the difference between a 2% error rate and a 20% error rate. And the last thing I say on this subject is, remember, we're not really talking in 97% of the cases about judges or jurors. We're talking about prosecutors, because 97% of all cases plead out so the person who needs to be educated here is the prosecutor so that [the prosecutor] doesn't fall into the kind of errors that a judge or jury might fall [into] because of their lack of education. I don't see any reason, for example, why, as part of the training that the prosecutors go through you couldn't have a half day on statistics. I think that would be a very valuable thing (J#1).

At first, when I started working with them, I was like, this is way too confusing and there's no way we're going to be able to do this in a way that's meaningful to people, but in some ways, I think there are some things about it that makes it more approachable (J#2).

Other (Academic Scholars)

I'm sympathetic with that perspective. I think it is the case. We know that even those of us who've been in this field for many years can, on a bad day, transpose [the] conditional and it seems inconceivable to me that lay people will not transpose conditionals, and that is just one symptom. I think of the fact that it is much harder for a lay person to really fully understand what they're being told in probabilistic terms. But on the other side of that, what they understand when they're being told in categorical terms is an over-simplification to the point of being untrue, to some extent. If someone is really reporting well and making sure that they say this is just my opinion and other people's opinions could vary, then no, it's not untrue. But, yes, I have sympathy with that perspective. I think there is a real difficulty in making sure that we explain these things well enough, but I don't think it is a strong enough reason not to do it (O#1).

I think they are right. It may be confusing to a lot of people, but I don't think that's a sufficient reason to go back to an unjustifiable alternative form of reporting. ... It's not easy to present statistics. We'll need to do it carefully and we need more research on how to do it best. But, from my point of view, we're stuck in a statistical world and we need to make the best of it. I don't think that the evidence supports claims that people are so hopeless that it's a hopeless task. ... I would say, although there is considerable

evidence that people can misunderstand statistics, and that is a problem that has to be dealt with, I don't think the errors will systematically favor one party or the other (O#2).

[I agree,] just ask someone on the corner and say, "I have this problem with fractions. I want you to solve it" and see what kind of reaction you get. So that informs me that for the average person who finds themselves on the jury, a deep understanding of probability is it's like asking them to solve Einstein's equations. It's just not going to occur (O#3).

Participants' responses related to findings from a recent survey of forensic friction ridge practitioners indicating 80% of respondents believe probabilistic reporting would be misused by defense attorneys to create "reasonable doubt":

<u>Laboratory Managers</u>

We want to make sure whatever we put out there is not going to be misused by either side so I want to make it very clear and I can understand people being concerned about it. … The last thing I want is to put something out there that can be misused. So, I understand the concern, but then that's up to us to write the reports in such a way that they're clear as much as possible through training that they can't be misused. … That's why you should have the opinion that we believe that this has a likelihood of association, then you throw in the number but you give the whole package as opposed to just reporting a number that potentially could be misinterpreted (LM#1).

I can understand that fear. I don't think it's a realistic fear. I think there will be a period of adjustment just as there has been with biology. There is a period of adjustment that people learning to understand what it means, but I can understand the logic of that when you're going from a circumstance of same things, categorically, without uncertainty to any uncertainty, how is that not reasonable doubt? ... [Whether this should be a factor that practitioners take into account,] I would like to say that people can be rational enough that this shouldn't be something that would be the driving factor, but reality is even people that pride themselves on being rational, really aren't. And that irrational fear of what may happen with a big fundamental shift like that, you can't ignore it. You have to respect the fact that as irrational as it may be, it's where people are at, so you can't exactly tell them they shouldn't worry about that because they're going to worry about it. … I am routinely struck with, even again, in rational laboratorians that seemingly are able to hold the dichotomy in their head of "I'm a rational scientist, I'm going to follow the evidence where it is, but let's go get the bad guy," or "I'm a rational scientist, I follow the evidence where it leads, but I'm an advocate for the downtrodden and those that are wrongfully convicted." Everybody struggles with that. I think there is a huge grade of the concerns that all come back to the fear of the uncertainty and they insert whatever their particular uncertainty is there. Whether it's, "we're going to lose cases that we shouldn't lose," or "I'm going to get beat up on the stand," or "I'm going to lose my job because I can't answer that question certainly enough" or "the juries are going to make the wrong decisions even though it's clear what this should be," their fear is if we change this, I don't know what's going to happen on the other side of it (LM#2).

It can be misused, yes. I don't know what the consequences of that will be. ... Could they be misused? Yes. Does that negate the importance of them? No. They need to be used appropriately. ... I think we as forensic scientists, we're always worried about how things are going to be misused. Things are always misused, for the benefit of trial and at least with the attorneys. If the probability studies had no benefit, then yeah, I'd say get rid of them because they're always misused, there's no benefit. That's not the case here. The probability studies are important. We're going to have to navigate through how they're used so that they're not misused. ... If you're implying that it's going to be misused, so it's bad. That's not what I'm saying at all. Everything can be misused by attorneys, so that's part of our business. Anything that's not complete truth can be contextualized to the point where you're not saying the whole truth. ... I think the staff need to know the limitations and the broad picture and how to explain, respond to certain objections, make sure that the assumptions the attorneys are making are correct versus false assumptions, so that [the] truth can come out. I think it's important for us to know how to deal with the information, but I don't think it means we should exclude it (LM#3).

Prosecutors

I guess that their concern is more like, oh, defense attorneys are going to use this as a way to try to undermine my opinion. ... Like, you're going to use a probability that maybe I don't really think is necessary or isn't really valid for whatever reason and you're going to try to make that seem like my opinion. You're going to try to use it to attack my opinion (P#1).

A defense attorney has an obligation to defend the interests of their clients. So, they can take anything in a case and try to create reasonable doubt. That's their job. So, whether they use a statistic and they get to take advantage of that statistic or the probabilistic reporting and use that as reasonable doubt, so be it. If I decide that this way of reporting is scientifically valid, I'm going to offer that. So, just because in any given case, any type of evidence may be an area of a reasonable doubt, we, as a prosecutor, who's going to offer that evidence needs to evaluate, well, is this truthful? Is it accurate? Is it something I should offer? And there's a lot of layers to the evaluation, whether we're going to use evidence, but for me, it would be improper to say, I don't want to use this type of science because a defense attorney is going to argue reasonable doubt. That's not part of the calculus for a prosecutor. ... The reality is, as a scientist, you should do what good science dictates. As a lawyer, we're going to argue on the law side, it's almost like, you know, scientists, you stay in your lane, you produce good stuff. Lawyers, we're going to argue stuff in court. And as much as you may try to please everybody, that will never happen, unfortunately. So, scientists keep doing good science. Lawyers, we're going to keep arguing (P#2).

I think that that we should be worried about conveying information clearly and cleanly. And so, if anything gets in the way of that then I think that's a problem. ... Shame on the prosecutor if they can't counter what the defense is trying to say. I mean, we see that all the time, you know, a defense attorney will say, well, reasonable doubt, they said they were 99% sure. That 1%, that's a reason for doubt, you know, there's all kinds of

locutions that lawyers use to try to create doubt. So, I don't see that as being particularly troubling (P#3).

Defense Attorneys

> *I think [forensic scientists] should stick to the science and let the lawyers worry about what we're going to say (D#1).*

> *I would call those results laughable if they didn't concern me so much. As we know for decades, forensic examiners have overstated the value of forensic evidence in just about every discipline. I don't remember seeing surveys of examiners concerned about overstatements at that time. ... So, the fact that 80% of examiners are fearful of that is not only laughable, but it's also concerning. Why are forensic examiners concerned about the outcome of the case? Why are they concerned at all about what jury determinations are in the case? Why are they concerned about reasonable doubt when that's the very thing that examiners should not be concerned about? ... The fact that 80% of the examiners in a survey are concerned about case outcomes based on shifts of how we report language to me shows the power of the unconscious bias in the criminal justice system. And it's really concerning that examiners are even worried about case outcomes. ... [This] would be shocking if I did not already believe that an overwhelming amount of pro-law enforcement bias exists in the forensic sciences despite the limited efforts of a few to identify and address it. ... Forensic examiners should be concerned about accurately stating the meaning of the forensic evidence and possibly whether the jury received the intended meaning (D#2).*

> *I think that probably the opposite is more likely. I think that it's much more likely that the jurors hear a probabilistic statement and they take it to be a categorical statement. I think it is far less likely that somehow defense attorneys would use it to present to the jury an argument that there should be less weight given to the evidence than what the underlying science shows. ... I just think that is ludicrous. I think that it's much more likely that the jurors are going to hear a probabilistic statement and take it to mean, you know, that X matches Y. ... I think that it's not for scientists to be opining on how the adversary system is going to understand or misunderstand the evidence. They need to present the science. Their concern that somehow something's being misused by the defense seems to be out of their lane, so to speak (D#3).*

Judges

> *I don't understand that objection at all. If you say it's my opinion this is a match and that's all you said, that's conveying a quality of certainty to something that in fact is not certain. If you, under cross-examination, are asked, "well, what's the error rate," what are you going to say? You're going to say, "I don't know," just probably the usual reaction. Then the jury is deprived of information that is available, that is out there, that if you had required a probabilistic response, the practitioner would have boned up on in advance and could give a response. I'm not sure what is meant by the objection that this might create a reasonable doubt. Well, that's what the system is all about, is finding out*

whether there is, or is not, a reasonable doubt. It sounds like those respondents didn't have much faith in juries (J#1).

I think we would need to stop being afraid of defense attorneys. I really do think that we just need to stop that nonsense. These numbers can be misused by everybody because they aren't being understood properly. I don't think a lot of it is even intentional. I just think that it is what it is. So, I think misuse happens for all sorts of reasons and it doesn't have to do with what side you're on. So no, I don't think that it should be a reason that we should not look at [probabilistic reporting]. Quite honestly, my experience has been that [many] defense lawyers are far more interested in actually understanding what the numbers mean and how those things are being generated versus the prosecution that seems to want to sort of just come into court and have it serve to them (J#2).

Other (Academic Scholars)

I think they don't want doubt introduced, [and] it scares me actually. It scares me that forensic scientists don't feel confident to talk through uncertainties and anything that is below a hundred percent. We, as scientists, should be comfortable in talking about the limitations of our analysis as much as the strengths of our analysis. It's the job of defense attorneys to introduce reasonable doubt, but it's our job to be sufficiently transparent to allow them to scrutinize the evidence (O#1).

[First of all,] creating reasonable doubt is what defense lawyers are supposed to be doing. If there's some reasons to doubt the finding, then the jury should know about them. So, the wording of the question kind of amused me—it's the presumption that creating reasonable doubt is a bad thing. ... [Second,] from my perspective, this portrays a mindset, which is that the goal of forensic science is to produce convictions and anything that gets in the way of producing convictions is a bad thing. I just have a totally different perspective on this. … We have to ask what is going to make our legal system operate most effectively. We're talking about optimal operation of a system. Usually, the optimal operation of the system requires getting the ultimate decision-makers the evidence they need to make a fair evaluation (O#2).

I would agree with this. Look, … what you have [in our legal system] is a back and forth between two sides presenting evidence. The point of the exercise is to convince the majority of the triers of fact that my side has done better on the argument than yours. So, if you have a tool in that process of back and forth, that lends more credence to the points that [one side is] making [compared to] the other side, then you're not going to want to give that tool up. The way that forensic science is currently structured, mostly that tool is something that prosecuting attorneys can use. … The "danger" of probabilistic reporting is that now the defense attorneys have this tool of creating doubt. So yes, I would agree with [the practitioners] … that it's likely to create opportunities for defense attorneys to abuse it. I don't disagree about that. [However,] that's why I want to put bounds around all this stuff. The bounds are proof of [the] range of reliability. That's what I keep coming back to (O#3).

Participants' responses related to the role/duties of forensic experts and the limits of their testimony:

Laboratory Managers

Our duty is to make sure that our testimony is framed appropriately—not underweighted, not overweighted. We don't want to have one side or the other to misrepresent, or for that matter, overstate what we're saying. At the same time, we don't want it to be lost in terms of [it] really didn't mean anything. ... It's got to be clear [and] it's got to stand alone. ... So, I'm a big fan of you really just can't just give a number runaway. You really should have some verbiage with it so people better understand. ... I get that there's been information that people [have conveyed in the past and they] shouldn't go that strong. ... I believe we're getting direction now to say we really shouldn't use these words anymore—it's overstating. So, I think while that might've flown [in the past] and still might be appropriate for [some situations], it's not appropriate anymore for [others]. You still might have that opinion, but guess what? The times have changed, the data has changed. ... I do believe there needs to be just a little more cohesiveness as an enterprise. ... I think we need to kind of come together a little more as an industry to make sure that we don't overstate or we understate (LM#1).

I think it is an inherent obligation on the part of the expert to convey those limitations and do the best they can trying to explain the inherent uncertainty there. Now the tricky part of that is that's not an easy thing necessarily to explain, even when you have quantitative measurements. ... It's almost more important that it is effectively conveyed on the report [rather than just in testimony in court] because [if] you think about it, our system of justice is not actually an adversarial court hearing. Our system of justice is a system of negotiated plea agreements that, at most, the decisions are made off that report. Approximately 97 to 98% of the stuff never sees the inside of a courtroom. ... [To claim plea agreements are made with full understanding of the limitations of forensic results] is bullshit. ... Most defendants that are dealing with that result have a harried, inexperienced, overwhelmed public defender who has no clue. [Compared to defendants that have the resources to hire competent counsel,] that playground is not level at all, not even remotely close to level. [However,] this is not saying that we have effectively managed to accomplish this, we haven't (LM#2).

I think all of us have an ethical obligation to understand the limitations of what we're saying. That's based on self-auditing, our experience, the papers we've read, how we come to our conclusions, following policies and the reasons why we have policy. So, all of that information that we actually use, I think ethically obligates us to present that information to the jury with a foundational uncertainty. ... [However,] most of the time the court hearings won't allow us [to express those limitations] unless they directly ask us. ... The laboratory isn't going to give you a number, because I don't have a way of showing you how I calculated it. So, articulating that uncertainty is something we're not perfect [doing] yet. But, it's also one of the reasons why we don't say to the exclusion of all others [for example] (LM#3).

Prosecutors

The roles and duties of forensic experts are to test the evidence and follow their rules and the best practices within their discipline and to accurately and impartially convey those opinions (P#1).

A scientist, in my opinion, should give their opinion as to what the science can say. The lawyer argues the value of that opinion and that's foundational in every aspect of a trial. … So, we, as lawyers, argue value of things, the evidence should speak for itself (P#2).

I think that forensic experts should bring their very best skill and training to whatever the task is and do it without bias. That's what I want. That's what I want from a witness. And that's what I want from my forensic scientist at every phase of the investigation and the trial, because sometimes the information is useful. Sometimes it's exculpatory. Sometimes it's inculpatory. Sometimes it doesn't bring anything to the table. ... You should testify about what the finding were in this case and that absolutely should be the limit of what your testimony is because you don't know what came before, what came after, you shouldn't know the " prior odds" in the case. So, I say stay in your lane (P#3).

Defense Attorneys

The role and duty is to not overstate the science based on a subjective belief in it, or what you've been told by a mentor that isn't verified in science. ... It's also dangerous for an expert to be offering subjective opinions as to the accuracy of their own opinions. That's layers of opinion. If you're giving an opinion in court, the presumption is that you're confident about it. Whether or not it's in your opinion as to this being the same source, that's for the jury to decide based on the associations that you've made and the appropriate reporting that you've done about your error rate under these same or similar circumstances, then it's up to the jury to decide. What we all know and what the social science research demonstrates is that lay jurors turn off their critical thinking when expert witnesses get on the stand, because jurors are looking for objective evidence to tell the story, they want somebody that doesn't have an ax to grind, doesn't have a stake in the outcome of the proceedings, and wants to do the right thing. They're going to be listening to an expert witness much more carefully and accept it much less critically than you would your typical lay witness whose biases were a little bit more easily exposed. ... We don't have a DNA expert coming in after they give the likelihood ratio and saying, and then in my opinion, it's from the defendant. You're just taking the data that exists that the science supports and the jurors are making their conclusion. ... And, your duty is if you make a mistake, or you fear that you made mistakes to go back and correct the record and that duty extends your entire career, your entire life, because when you're dealing in criminal law, you're dealing with life and liberty issues. ... There's an ethical obligation to do that (D#1).

Forensic experts have an ethical as well as a legal duty to accurately state the weaknesses and limitations of their forensic method. But forensic examiners don't take this duty seriously. In my 20+ years of litigating many forensic cases, I have never

encountered a forensic examiner who took this duty seriously. The limitations and weaknesses are never documented in written reports. And examiners never admit to them on direct exam. It is always a game of hide and seek for examiners. And this game of hide and seek is exacerbated by the fact that many forensic examiners refuse to discuss the fundamental literature in the field. The weaknesses and limitations of every scientific endeavor is reflected in the peer-reviewed literature. While this was not the case for decades as fingerprint examiners were overstating the probative value of fingerprint evidence, a lot of literature is now available. But too many examiners refuse to acknowledge it. They refuse to discuss the literature in pre-trial interviews. And they refuse to engage in meaningful discussions of it during cross examination, often enabled by judges who don't understand any of it. Examiners will continue to play this game until they are clearly directed to, one: accurately document the weaknesses and limitations of their method, and two: read, understand, and discuss the fundamental literature in the field. Without both of these, examiners will continue to overstate the probative value of forensic evidence and evade real discussion of the science, and whether a jury gets accurate information will depend on the chance that the defense attorney is unusually prepared and whether the judge grants a proper scope of cross by allowing discussion of the fundamental literature (D#2).

The role and ethical obligations are for [forensic scientists] to, one: clearly and accurately report the information that they intend to present. Number two: they have an obligation to be willing to meet with both sides and explain their findings and explain any limitations of their findings. Then, number three: when they present the evidence in court, they need to be clear about what the limitations are of their findings. ... [When] they're answering the question, they're answering the question fully and accurately. Frankly, I think that examiners set up a little bit of a straw horse where they say, "well, we're not asked that question." I think that most of the information they convey, if they think about what the question is, they could present a more robust answer then they do, and in some cases choose not to (D#3).

<u>Judges</u>

No (J#1).

I struggled with this question because I really do think that an expert who was on the stand really does need to be answering the questions that have been put to them by the lawyer, and we have mechanisms for how it is that we want to expound. If there's an issue that's raised by one side that the expert is not allowed to provide additional information on, the other side has the opportunity to elicit that information. So, there's a court process that is sort of layered over top of what it is that I think an expert can do in being proactive about explaining those things. On the other hand, I think that experts that do explain the basis for their underlying conclusions are far more compelling and better experts. I do think, frankly, the rules of court require that you have a foundation for your opinion. So, from that standpoint, I think that they should be allowed. How it is that an expert can be proactive about it, I was thinking is that some of that proactivity should really be being done at the front end and should be considered in what's being provided

as part of the report that's provided to counsel in the case, maybe it's part of the trial prep that goes on between counsel and the expert. I think those are places where experts have a lot more opportunity to be able to work with the lawyers about why it is that it's important for them to explain [and] what it is that they'd like to tell the jury. So, I think that it's just limited by the rules of court and the relationship between the expert and the lawyer, which, whatever lawyer it is that might be working with that particular expert. ... [That said,] if someone is being shut down about testifying about the limitations of a particular testing that was done, that is a place where it is that I think it's fair game for an expert to say, "I'd like to be able to answer your question, but the answer to your question is premised on some information that's also important." I do think that there's some of that that really is appropriate and it's really hard because I know experts get pushed into this all the time, ... lawyers are imposing language upon them [for example, language such as "reasonable degree of scientific certainty"], and they really feel pressured to respond in a way that they think that is what the listener wants to hear. So those are areas where I really do think it's worth pushing back to some degree on and you know what it is that's being elicited in a courtroom (J#2).

My view is that [would be] called ipse dixit—"it is because I said it is," and, under the Daubert standards, the Supreme Court standard for the admissibility of an expert opinion, that's not allowed. So, there would [also] be a good preclusion motion under a state court standard for admissibility, like the Frye standard that's even more exacting. So, it should not be allowed. Every judge should require that an opinion be backed up by the reasons for the opinion and that, if an expert gets up there and says, "based upon my experience, this is just the way it is," ... I would say that that's an unreliable opinion. It's ipse dixit (J#3).

<u>Other (Academic Scholars)</u>

I think the role of a forensic science expert is to assist the court, not the prosecution or the defense but the court, in its evaluating evidence and to use their skill and knowledge that lay people don't have to help evaluate the scientific findings in a way that is helpful to the court—that is transparent about strengths and limitations. ... If I was to balance what should an expert do in terms of expressing uncertainty, I think they have to err on the side of making sure the court really gets the point that there is uncertainty as opposed to erring on the simplification and ease of understanding side of things. ... I think it is the role of the court to conduct that final reasoning in the light of the uncertainty that exists. I don't think it's the role of the expert to take that uncertainty away from the court if they don't have the scientific basis to do so, [but] if they have the scientific basis to do so, [then] fantastic, fire away. I think we just need to be so careful not to try and be so helpful to the court in helping them to get rid of the uncertainty that they don't like [such] that we stray beyond what we can robustly and scientifically say. It's something that I would say I've observed anecdotally over the years. Forensic scientists want to be terribly helpful, and I think that pushes the community sometimes to give an opinion on something that is too uncertain to give an opinion on in the first place. ... I don't think it comes from any desire to do anything wrong. I think it comes from a desire to be helpful, but I think it's dangerous (O#1).

I think the first duty is to get it right—to say things that are justified scientifically [and] to not go beyond their expertise and not claim more than the science will support. That's duty number one. Do not make unjustifiable claims. Then duty number two is, once you've identified the various claims that might be justifiable, try and choose among them in a way that promotes better understanding for a wider range of people. When in doubt, maybe present the evidence in multiple alternative ways and focus on transparency and a fair characterization of uncertainty. ... [It's] not that opinions of forensic scientists aren't valuable, but we have to acknowledge our own limitations. There's a need for scientific humility. The overwhelming tendency of experts in multiple domains is toward overconfidence. If we're going to be good scientists, we need to combat that by adopting norms that emphasize when in doubt [to] make the more modest claim rather than a claim that may be too bold (O#2).

I would hope that a forensic expert would limit their testimonies so that it was scientifically defensible. That means avoiding statements that you cannot show having an observationally true basis. ... that are repeatably observable. ... We are implicitly talking about repeatability and reliability (O#3).

Participants' responses related to whether it is acceptable for experts to express their opinion in court without disclosing the underpinnings or statistical data to support those opinions:

<u>Laboratory Managers</u>

I would strongly encourage they do it because I feel it makes their opinion better, stronger. ... [However,] I think there are probably some straightforward circumstances [where it is not necessary] ... [and] you don't have to go into data. Other ones, it's probably not acceptable if you don't give that. ... [That said,] it takes a few seconds more. It's just a fuller testimony (LM#1).

There is my answer to this, and then there is where even we are at, [which] are two different places. No, I don't think that's acceptable, but there is an enormous effort between where the world is at, even on the well-funded, well-intended, pushing the envelope end, and where we need to be. There is still a huge gap there. And the gap extends beyond just laboratories. I can whip my people in having that answer every time and it still wouldn't work because on direct, you have a puppy DA that can't find their way out of a paper bag to ask the questions to allow them to make that answer. And on cross, they get cut off and you've got a judge that's hostile to anybody answering squat. And even when you've got an expert sitting on the stand, getting confronted with an inappropriate question that they are trying to say, "I cannot answer your question as a yes, no answer," the court won't let them do that. You're stuck. That aspect of getting all of that underpinning there is not just a laboratory issue. It is a prosecution, defense, and court issue. And all of those things have to get fixed for that to legitimately and routinely be there. ... And, it happens even more that you've got the puppy DA and the inexperienced, overwhelmed public defender. They aren't even asking the questions. They

aren't even giving the opening for you to be able to insert the answer. The court room environment is not allowing for that part to be there. … This is one of the things that I'm finding myself getting a little bit more worked up about these days, of this issue of it was the laboratory that didn't express the extent and limitations of the testing. No, the lab is willing to do that, the lab wants to do that, all the rest of the system cut it off at the knees (LM#2).

[Not disclosing the underpinnings is] not the best answer, and it would be better to talk about the certainty of that conclusion. We're not always allowed to do that. I don't think it's a wrong expert opinion to give. It's just not the best that could be given (LM#3).

<u>Prosecutors</u>

There are specific rules of evidence that govern expert testimony in any jurisdiction, and they differ jurisdiction to jurisdiction. [In my jurisdiction], technically the expert doesn't even have to discuss the basis of their opinion. But they can be asked about it on cross. Again, this is where I'm going to say, you know, it would be bad practice as an attorney to elicit an opinion from an expert without having them discuss the basis of their opinion. But if you're talking about statistical underpinnings and things like that, there aren't always statistical underpinnings factoring into an expert's opinion. So, a firearms examiner, I don't think necessarily has statistical underpinnings, when he's doing a side-by-side comparison, you know. If you're getting more at, do I think it's appropriate for them to articulate their opinion without getting into things like error rates as a condition precedent of them giving their opinion. Yes. I think it's appropriate. And I think that that's for the legal side to decide (P#1).

The rules of every jurisdiction are going to differ slightly in this way. But a lot of these issues that you're now raising are the specific issues that are dealt with in pretrial admissibility litigation and hearings about whether or not there's a sufficient foundation for the evidence to be offered in court. Once that hurdle is overcome, then I don't think there's a need to then further explain the scientific basis for it and all the research that surrounds it in trial. … I mean, if you think about it, it could really go down a rabbit hole there. If I was going to do that as a prosecutor, I would then offer every single study that refers to the reliability of the discipline. And we would then spend days reviewing that literature potentially then calling the designers of those studies to further delve into the methodologies and reliability of those conclusions. I feel like you start going into a way well beyond the scope of your trial. And that's one part of it, pretrial admissibility challenges. The other part of it is, the job of the defense attorney is to cross examine the witness. So, if they think that their conclusion is unreliable, then use whatever you think is necessary and appropriate to challenge that conclusion. And that's the essence of challenging a witness through cross examination (P#2).

I think there needs to be some data, but the data could be the two items and the fact that, you know, if you're in ballistics, you can explain how metallurgy works and every land and groove mark is going to be different. And you can explain the consecutively

manufactured barrel studies. All of those things are possible. I think any expert should be able to do that. Whether that's necessary in every trial? I would say it's not (P#3).

<u>Defense Attorneys</u>

No opinion should be entered into evidence without a thorough examination for the basis of it. The whole reason that we have a confrontation clause and cross examination is to examine the basis of the opinion. If you're just giving an opinion, then there's nothing you can even cross examine about giving that. Well, I just think that's so based on my training and experience, where do you go from there? It is so, because I say so, right. That's why they call ipse dixit, that's why science rejects that (D#1).

No (D#2).

[Forensic scientists] have an obligation to provide the supporting data, but also the limitations on that data. The forensic scientists that are more steeped in science tend to offer there's a hundred studies, but they don't tell you, for example, that 98 of them are closed set studies and what that means, or that in the two studies where the error rates are 0.01, that the majority of the people who returned the answer answered inconclusive and what that means. ... That's a real limitation on these studies that the examiner has never seemed to bring out, ... [and training and experience] are just not a legally sufficient basis for an opinion. It's something that the court considers, but it's one small factor along with a number of things. ... [It's been admitted in the past because] for years and years and years, the defense bar really was, frankly, not educated and did not do a particularly good job of starting to bring to courts the problems with all of these disciplines. So, there's this whole body of case law that's based on either no litigation or very poor litigation. ... Unfortunately, the courts rely on precedent. So, bad precedent builds on that precedent. I think judges and defense attorneys are starting to be much more educated and are beginning to understand many of the limitations of the forensic disciplines. So, the courts are now starting to limit them to what is scientifically shown or proven are valid. ... The education of defense attorneys has sort of upped the game (D#3).

<u>Judges</u>

No (J#1).

I struggled with this question because I really do think that an expert who was on the stand really does need to be answering the questions that have been put to them by the lawyer, and we have mechanisms for how it is that we want to expound. If there's an issue that's raised by one side that the expert is not allowed to provide additional information on, the other side has the opportunity to elicit that information. So, there's a court process that is sort of layered over top of what it is that I think an expert can do in being proactive about explaining those things. On the other hand, I think that experts that do explain the basis for their underlying conclusions are far more compelling and better experts. I do think, frankly, the rules of court require that you have a foundation for your

opinion. So, from that standpoint, I think that they should be allowed. How it is that an expert can be proactive about it, I was thinking is that some of that proactivity should really be being done at the front end and should be considered in what's being provided as part of the report that's provided to counsel in the case, maybe it's part of the trial prep that goes on between counsel and the expert. I think those are places where experts have a lot more opportunity to be able to work with the lawyers about why it is that it's important for them to explain [and] what it is that they'd like to tell the jury. So, I think that it's just limited by the rules of court and the relationship between the expert and the lawyer, which, whatever lawyer it is that might be working with that particular expert. ... [That said,] if someone is being shut down about testifying about the limitations of a particular testing that was done, that is a place where it is that I think it's fair game for an expert to say, "I'd like to be able to answer your question, but the answer to your question is premised on some information that's also important." I do think that there's some of that that really is appropriate and it's really hard because I know experts get pushed into this all the time, ... lawyers are imposing language upon them [for example, language such as "reasonable degree of scientific certainty"], and they really feel pressured to respond in a way that they think that is what the listener wants to hear. So those are areas where I really do think it's worth pushing back to some degree on and you know what it is that's being elicited in a courtroom (J#2).

My view is that [would be] called ipse dixit—"it is because I said it is," and, under the Daubert standards, the Supreme Court standard for the admissibility of an expert opinion, that's not allowed. So, there would [also] be a good preclusion motion under a state court standard for admissibility, like the Frye standard that's even more exacting. So, it should not be allowed. Every judge should require that an opinion be backed up by the reasons for the opinion and that, if an expert gets up there and says, "based upon my experience, this is just the way it is," ... I would say that that's an unreliable opinion. It's ipse dixit (J#3).

<u>Other (Academic Scholars)</u>

I think it is really important to disclose the basis of your opinion. I think when it comes to the actual courtroom, [however,] it depends on so many things—what you actually say in testimony. When it comes to your written statement of evidence and your case file, that contains all your notes, [however,] I think that underpinning has got to be disclosed so at least it should be available for scrutiny by whoever in the court process wants to scrutinize it. I think that when we just give unqualified opinions, it is almost impossible to challenge really, because if you're not giving a reason for your opinion then it just comes down to, "well, that's my opinion" (O#1).

No (O#2).

No (O#3).

Participants' responses related to what they would describe as the greatest challenges facing the pattern and impression evidence disciplines as it relates to examination and reporting methods:

Laboratory Managers

> *Keeping abreast of the technology and how the movement of the data and the philosophy of things are happening in a discipline that used to be very manual that is becoming more and more algorithm and computer assisted. Keeping up with that when you you're still giving opinion evidence is a real challenge because you have a mindset [shaped by] what you learned, and whatever you learn, [to you] it becomes right. ... The challenge for that group of individuals to keep up, to feel they're part of it, to stay on top of it, especially when you may have some people that are not as savvy in some of these things (LM#1).*

> *The trivial answer is just money. I say that's trivial because there's really a lot of nuances under that. Really it is a matter of laboratories, writ large, the entire system is so wildly under resourced. ... Then the expectation of what people think is occurring, just doesn't match the reality. I keep finding myself in many circumstances saying, "yes, there are very real issues of science that we can't lose sight of, [such as] how many points of minutia make up a sufficient circumstance for identification? [or] quality algorithms to be used on [fingerprints]." That's great, but when we're dealing with simply the evidence coming in the door, being fundamentally flawed, those matters of science, don't matter. ... Most of the stuff coming through the door is illegible, mixed up, damaged, contaminated, and really inappropriate to use. So, yes, there are real science things that we can't miss, [but the] biggest challenge is you've got shit coming in the door—of course, the answer coming out the other end is going to be shit. The biggest challenges are the enormous amount of effort and patience and capital that's sucked away in, at best, modestly competent information systems that the laboratories run on. Basically, you've got analysts working in near third world conditions. Those are really the biggest challenges. Yes, the answer of money is trivial, but until we solve some of these things that affect every case for the entire system, all the way through, honestly, the science-y stuff is a little a bit of a privilege to think about (LM#2).*

> *I think as a group we need to integrate and develop probability-based studies into our work quicker. We are moving too slow in this story, and I think some of this is architectural and practical. ... We need to get these models working in the laboratory side-by-side with the expert witnesses, that's the way we're going to be able to give the best information to the jury—by having both the expert witness and hard objective models. ... [But,] these algorithms aren't easy. There's a separation between academically available data and a crime lab available data. Labs and academics, research groups need to work closer together. There is still a little bit of resistance that you're taking away the expertise [the experts] already have and supplanting it with something else. That, to me, I think is completely false if you agree to integrate them both together. If you want to completely replace an expert, then I'm going to be opposing you because I don't think it's appropriate either. I don't have a problem with the numbers. I just don't think the numbers themselves are the best model. ... The other biggest reason is*

that [for] crime labs, it's not our mission to do research, unfortunately. I love research and it's wonderful, but we are under so much pressure to get casework done. We just don't have the time, energy or money to do it. It's unfortunate because we're really the best place to do it, but we just don't have the money to do it (LM#3).

Prosecutors

Lawyers. I mean, there's really no other way to say it. So, I'll give the caveat just because I feel I need to give it like, yes, I am a prosecutor, but I want accurate and scientifically sound forensic evidence. I don't want opinions that are inflated. I want you guys to decide what is accurate and what the limits within your discipline are. And then that's my evidence and I deal with it in my case. I think probably the biggest challenge that the pattern disciplines are facing is what I'm going to call sideways attacks. I think that your disciplines, sometimes under the guise of cooperation, are being undermined and encouraged to render yourself obsolete. I guess this would be the best way to say it. I really do think that lawyers right now are your biggest problem, lawyers and academics. You need to let us into a certain extent, but you got to kick us out too. And that goes for prosecutors too. Like you got to let us in to hear what we have to say, because it does help you. And, I know it looks great to play nice in the sandbox, but there is value to hearing what we have to say, but you have to know when to stop listening, and to realize that not everybody has a crystal-clear agenda (P#1).

I think it's a bigger issue that's happening in the community, is to understand what the conclusions are and what the limitations are, and to ensure that we're staying within those boundaries (P#2).

I think the challenge is that practitioners and people like you are attempting to appease the defense bar and that's never going to happen. ... You are never going to satisfy the defense bar because we are in an adversarial system. You're never going to have the defense bar saying, you know what, we're satisfied. You have done a great job. Because it's part of the adversarial system, but they are trying to dilute the impact of forensic evidence that implicates their clients while at the same time, and nobody ever calls anybody out on this, if the forensic evidence supports their theory of the case or tends to exculpate their client, then it's the gold standard of whatever the discipline is. So, I think that's the biggest challenge. It is this feeling among the disciplines that they're going to be irrelevant if they don't agree with the defense bar, because the defense bar has many seats at the table. You know, I look at the composition of the OSACs and I look at the composition of various committees, and I see as many defense attorneys as I do see scientists and prosecutors combined. So, I think that the challenge is trying not to fold in the face of that kind of pressure (P#3).

Defense Attorneys

This digging in on the way that this has always been done because of subjective belief that there were no problems with it or because there haven't been tons of wrongful convictions associated with it, is sticking your head in the sand. We know what's lacking

in these techniques, read the PCAST report, read the NAS report, read the AAA's report fingerprints. These are the top scientists in the country. Just because forensic folks disagree with them doesn't mean that the forensic folks are right. ... I should probably withdraw that . ... Overstating conclusions, you know, is a fast way for wrongful conviction and for us to have to go back and examine tens of thousands of cases where this has been done. The challenge is that courts will . . . . [well, . . .] I don't know, you know, actually, the truth is there may be no challenge, courts just may not care, because we don't care about the rights of the indigent defendants. In your typical criminal cases, the challenge is scientific integrity. The challenge is trying to claim science when you don't have any (D#1).

In pattern matching, I would say it probably continues to be the lack of empirical research. So, take fingerprints, we know so much more about it now than we did a decade ago in terms of all these empirical studies and they have been so important to me, at least in understanding the limitations of the method, and to their credit, the fingerprint discipline is way ahead of other pattern matching fields in that regard. So, all of those big studies, many of them have not been repeated in any way or in as good a way as fingerprints, and yet, even in the fingerprint discipline, there's more to do (D#2).

To do the research that's necessary and have it done by people who are independent of the discipline, who don't have any interest in the admissibility of it. Let's see what the research shows us, and then let's learn how to present it accurately in the courtroom. I can tell you that there will be far fewer admissibility challenges and far less litigation if that's done. ... I think it's in reach. It seems like there is funding now for this research, there is energy behind it, and there should be the incentive to do it (D#3).

Judges

Good, blind, scientific testing. That's not my conclusion. That was the conclusion of the National Academy of Science. So much of this has just been "seat of the pants." Most of the forensic sciences are developed by police as investigative tools and, for an investigator tool, it doesn't matter whether it's subjective or not. If it gives you a helpful lead that you can trace out and see whether it pans out or not, great. But then, beginning in the early 1900's, with fingerprinting, it began to be introduced as hard evidence in court and people forgot that it had never been subject, with the great exception of DNA, to serious testing. But it's not as if it couldn't be tested. So, I think that's the greatest failing (J#1).

Other (Academic Scholars)

I think that the sort of ongoing narrative of forensic science in crisis can be really unhelpful to these disciplines because there is a huge amount that we can confidently say in these disciplines. If we are honest about our limitations, then [forensic science] can still be of real assistance to the courts. I think one of the challenges, really, is this ongoing "until it's perfect." It's all awful narrative, which I think is just unhelpful. I think if there was more of an acceptance of imperfection, with clarity and disclosure of that

imperfection, then we would be able to move ahead in a more step-wise fashion and just keep improving rather than [what seems to be] a desire for a jump from, as I say, "terrible" to "perfect." That's just never going to happen. We just have to keep pushing at this from every direction (O#1).

If the field is going to continue with relying on the human brain and a human assessment of similarity as the major instrument for making assessments, then it's very important that we do assessments of the accuracy of that instrument. So, one challenge is validation of the accuracy and performance characteristics of human judgment. If we're going to do an algorithmic approach, then obviously we're going to have to validate that as well. So, I'd say regardless of how the examination is done, the greatest challenge for the examination is validation. Then, on the reporting side, the challenge is how to present the findings in a way that takes into account both the strengths and limitations of the performance of the method as revealed by the validation studies, and if we're in an area where validation has not been done or is incomplete, how to acknowledge that in a forthright manner. ... I [also] think it would be a mistake to assume you can do a single black box study and [assume] you're done. The "one and done" approach that I've heard a lot of people take is clearly not realistic. ... Knowing what the limits of the human instrument are in terms of accuracy is really important for the overall operation of the system. From my point of view, validation and performance testing should be a continuing part of the job. It should be incorporated into lab work. If we incorporated that kind of routine empirical testing into the way we do casework, it would make us more of a scientific discipline (O#2).

The greatest challenge that I've observed is actually resources. ... I have had a chance to see the conditions that real forensic scientists work under. They're not the conditions that Hollywood tells the public about. The real conditions are often overworked people [and] under-resourced people with no time to get the results out. I mean, that's the real world. To me, that's the greatest challenge to forensic science, to convince our society to put in the resources so that people can do the best job, so that this intuitive expertise that I [believe forensic scientists have], is actually allowed to work without having the pressure that can induce errors (O#3).
