# Supplementary material for: Probabilistic reporting and algorithms in forensic science: Stakeholder perspectives within the American criminal justice system
Source: Forensic Sci Int Synerg. 2022 Feb 12;4:100220. doi: 10.1016/j.fsisyn.2022.100220 (PMC8850671; doi:10.1016/j.fsisyn.2022.100220)
Supplement: Multimedia component 6 [file mmc6.pdf]

Appendix VI

PARTICIPANTS' ELABORATED RESPONSES RELATED TO THE USE OF ALGORITHMS

Participants' responses related to the use of algorithms in court and the benefits and risks/limitations of them:

Laboratory Managers

> *I think that's an excellent thing to assist in better understanding why you came up with this opinion. But the danger is that people then rely too much on the number and it's really for the expert to frame that, to help you with understanding how much weight there is on what I'm saying. The algorithm is not testifying today. I am, and this is what it means. I think [an algorithm] just really is going to help them. If framed and used correctly, recognizing that much of the time we're not testifying, it's a report that has to fly and we just don't want to throw up the number without all the basis for it, the strengths, the limitations, just so people have a crystal-clear understanding because if they're going to base their decision, plea bargain, [or] whatever, it needs to be as clear as possible for them—on both sides (LM#1).*

> *Yes. I think ultimately they have a very real and very large role. I think the greatest benefit on the algorithms is the relative consistency of the result case over case. The ability to engineer the system for accommodating the fact that these things [our heads] are biased engines. That's not bad thing, [but] there's a lot of advantage in the compliment of that bias engine with a [algorithm] that's going to do the same thing every time, [it] could get you much closer to reliable results. ... There's going to be less variance because the person's kid is having trouble in school, or they're not feeling well, or, they're grossly underpaid, or it's cold, or they haven't slept. It's going to even some of that stuff out. ... [Further,] they are a force multiplier for analysts. No way, is anybody ever going to cough up enough resources for there to be actually enough analysts to do what everybody thinks is getting done. So, somehow algorithmic compute-based tools are going to have to amplify what analysts we do have. So, I think there is [also] an enormous role in simply from [a perspective of] building capacity. ... [However,] I think the biggest risk is becoming overly reliant and we just exchange the categorical certain answer from the spectacle nerd for now, an infallible algorithm. ... The pitfalls are the desire to want it to replace the analysts—the desire to view it as a cost savings thing that lets me get away from having all these analysts. ... And, I would hope that a lot of these facial recognition things are a cautionary tale for everyone that depending on what those algorithms are originally trained on, builds biases into the algorithm, and those are biases that you can't wash them back out (LM#2).*

> *I agree with using algorithms. I think it's something we need to do and should do. I do not agree with that being the only thing we do. I want to use algorithms and I want the expert with their experience and so forth—both hand-in-hand going to court. ... The benefit of*

using the algorithms is [that] it's a little bit more standardized methodology across the industry so the rules are a little bit clearer for everybody on what to use [for examination and interpretation purposes]. ... The disadvantage is that we stop there and don't use the other expertise and training and methodologies that the person has, which is huge, as human beings are so good at looking at things without breaking down to individual pieces, looking at things holistically and saying there's something wrong or something different. That skill, while it may not be objective and something you can attach a number to in a computer, is very, very valuable. So, the negatives here are doing one without the other. They both have their strengths and they both have their weaknesses—[particularly] when they're done exclusive to the other (LM#3).

<u>Prosecutors</u>

For the pattern disciplines, based on what I know at this point, which I know very little about what's available or what the underpinnings of them would be. I don't think that it's necessary. I think it would overly complicate things and I would not be in favor of it at this point. Again, my mind is open to be changed as if I was presented with evidence that said, okay, here's how we can compute how rare this particular fingerprint impression is. I would be open to hearing that. ... Math is hard. People don't like math. ... I think that it can be confusing and, again, if there's an accurate way to report something that's less confusing, that would always be my preference across the board. But I do think with DNA, it is necessary (P#1).

Algorithms can add value to the case in so far as giving weight to whatever the conclusion is. So, in that way, it's helpful. ... The more data that exists that underlies whatever the algorithm is, I think the more likely it is that there's going to be buy-in from all stakeholders. ... As long as it's based on appropriate data, I have no problem with it. ... [But] the risks go back to whether there's a sufficient body of data that supports whatever the conclusion is. ... And in context of the case, does it depend on the robustness of the database which is being. Let's say it's fingerprints, are we looking at the likelihood ratio in relation to [my entire city], just [my] county, [my entire] state, country-wide, worldwide? What is the database? And then, how strong is that conclusion and how appropriate is that likelihood ratio based on the evidence (P#2).

[Algorithms] allow the scientists to do computations in seconds that would be undoable in a human timeframe, and so it gives you way more information and helps you weigh the evidence. I think more information is always good, so, yeah, I'm totally in favor of that ... if it were valid. I think it's working very well with the DNA [but] I do not see how we establish the numbers or the levels of confidence in pattern matching, because quite frankly, unless you are completely confident, it's an exclusion, it's not a match. ... Again, I don't see how we come up with one that's valid [but if we did] the challenges I think are explaining it to a jury. ... [Ultimately,] anything that increases the accuracy of the forensic science, I think is useful. The bottom line is if you, as a forensic scientist, have confidence, then I have confidence in the result. So, whatever makes you more confident, I think works for me and works for the court system (P#3).

<u>Defense Attorneys</u>

As long as we have transparency. You can't go into criminal court and just say, the black box told me this, and so therefore it's so. I spent a lot of time litigating around that issue. I'm for progress, but not by proprietary companies seeking to make money and not being transparent about the data and allowing opposing experts access to that data to examine the basis for these opinions. ... The greatest benefit would be is that you move away from unsupportable categorical claims into something that has some empirical basis to it and that you would actually have a number that's based on a valid statistical database, a population frequency database that is transparent and known. That's progress. That's something where there's real scientific efforts to be more accurate, more precise about what it is you're saying when you're "matching" a latent to a person. [But,] I'm never not going to be concerned about proprietary software being used in these circumstances. ... [Overall,] I think that there's a place for [algorithms]. I don't want to say anything that would retard progress, but there are some things that look like progress and sound science-y that aren't, and I think it's particularly dangerous to introduce new technology or new ways of expressing conclusions that are no more grounded in empirical data than they were previously. They may sound more modest, but in many ways they're even worse, because now we've got some algorithm, now we have a machine, and so therefore it's better, it's smarter. We, as human beings, the faith that we place in technology is [significant]. We don't need any more evidence than you and I are talking right now—the computer said so (D#1).

I don't think the use of algorithms is inherently good or bad in forensic science … there are some obvious and undeniable benefits [such as] speed and expense. Algorithms can handle things much faster than humans and on a much bigger scale … but, I think one thing that's fairly inherent in the criminal justice system and the use of the algorithms is that they are often used before we know how good they are, before we know the strengths and weaknesses, before we know how to judge whether it's operating well in this case or any case … [and] police, prosecutors, and judges accept the evidence because it is computer based, believing that because computer code is involved, it must work. ... Experts who testify about algorithms have no idea about the human imposed parameters of those algorithms, so they can't even begin to explain any decisions that went into how that algorithm operates and any limitations or weaknesses and how those limitations or weaknesses might impact this case and this testimony and this evidence. … In my experience, it's way more difficult to figure out when these systems fail in the criminal justice system than elsewhere. Ground truth is so murky. It can be really hard to figure out when these systems work and don't work in the criminal justice system. [That said,] I think algorithms should have a role [in forensic science]. I think, when algorithms replicate the ability of human examiners in their interpretation, I'm much more comfortable with that use of an algorithm. And if I'm comfortable that proper validation has been done, that there has been meaningful oversight of that validation by people not impacted by its implementation and that examiners give proper caveats about the outputs of those algorithms, then I say go for it. … [However, I am concerned that] inevitably they will be used in the criminal justice system in a role that far exceeds what I'm calling for (D#2).

That's a complicated question. I think in the long run it is something that will improve forensics. In the short term, I think the problem is there needs to be significantly greater transparency. The fact that there's objectivity is what is great about algorithms … [but,] the limitations of algorithms are all the assumptions that go into the creation of the algorithms and the ability of the person presenting the results, as well as the end users understanding how the algorithms work, and what the limitations are of the information that's being presented. I think that's challenging. ... There needs to be scientists from a variety of different backgrounds involved, weighing in, and there also needs to be some significant oversight. ... If they're used, there needs to be some type of accreditation and enforcement. ... [Overall,] I think there's a role [for algorithms], but I think it's just really important for everybody to understand globally that the machines are only as good as the information that they consider, and there are real limitations. I think we are just moving way too fast and we need to take a pause and really start to understand and accept that machines have all of these same limitations that humans have and they are not the answer to all of world's problems (D#3).

<u>Judges</u>

I think algorithms can be helpful, to a degree, if they are totally transparent. When you say, "oh, here's an algorithmic formula," you give the impression that, "oh, this is something like calculus." But, in fact, it involves all sorts of choices on the part of the person who puts the program together. So, that has to be fully transparent and open to peer review so that you know whether it's a good algorithm, a bad algorithm, an algorithm that has a high error rate, an algorithm that has a low error rate, and so forth. ... I think really good algorithms could reduce the subjective portion of the analysis. ... [However,] some companies are obscuring inquiry through trade secrecy laws, but even where that doesn't operate it's very hard for even defense counsel [to review]. ... Even in those states where the trade secrecy law objection is overruled, they have to hire an expert. You can't expect a defense counsel to be an expert in these algorithms. In many states, there's no money available to hire that kind of expert. Many states, even where you can hire an expert on the other side, the expert gets very limited disclosure (J#1).

I don't necessarily have a problem with using algorithms. I think there are likely reasons that [they are] beneficial in doing some analyses that we just aren't capable of doing without the use of computers. For me, the biggest thing is transparency. I think if you're going to utilize algorithms that the algorithms that are being utilized need to be transparent as far as access to their source code [and] access to the assumptions that are being placed into that algorithm, making sure that there's equal access, for everyone, to be able to either utilize the software in whatever way it is that they want to utilize it for answering their own questions or at a minimum that there's access for purposes of doing appropriate research. ... The best way I can say is transparency. For me, that's what it's mostly about. ... If they're being utilized with assumptions baked into them, that aren't known, I think there is risk for the potential for misuse. ... [Further,] if you have something that you're not transparent with, [then] the assumption is you're hiding something. This is very big in the culture, I think, in criminal law in particular, and a lot

with defense lawyers [who] assume that if they aren't given access something, it's because you're hiding something. I think the community is getting more and more to the point where if you aren't being transparent with something, they think you're hiding something. So, from [that] standpoint, if we are utilizing algorithms that we don't understand and that we haven't provided enough transparency around how it is that they're actually doing what it is that they're doing, I think that we erode the confidence in the analysis as well as potentially in the system itself, and that's where I think it becomes really concerning (J#2).

I think that algorithms are here to stay. ... There's a great potential [with algorithms], [if] done correctly, to create criminal justice reform to a degree that we've never seen before. There is extraordinary potential for that, because there will be an ability, if these tools are designed correctly and they're validated correctly, and they have the right degree of trustworthiness, including [this concept of] fairness, they have an ability to take out some of the human biases that have plagued the criminal justice system. So, I think there's great potential, ... but there are certain risks. ... What we need is a national conversation on what that means and how to create trustworthy and reliable algorithms that can be used for individual liberty determinations. That's where the rubber meets the road. ... The greatest risk is that we allow complex design and complex tools to just snow us a little bit ... [and] that we don't have these conversations as to what fairness means and what fair design is and what trustworthiness is in time (J#3).

<u>Other (Academic Scholars)</u>

It depends on the algorithm. ... [In general, the benefit] is performance [and the ability] to program algorithms to do things that humans can't do. ... I think that using computational algorithms that the reporting scientist understands the basis of and is able to explain is a really good thing. Using, let's say at the other end of the spectrum, algorithms based on machine learning, which have come from, let's say a manufacturer who won't disclose the training set, and that the reporting scientist doesn't understand [or] can't explain and come talk about any biases, for example, or any limitations [of it], is deeply problematic. ... [Further,] I'm not convinced that there is a legal basis on which to introduce that evidence, because who is the expert is the question that I then come to, is it the algorithm? It can't be the algorithm because you can't cross examine an algorithm, or is it the expert who's giving the results of the algorithm in which case that expert has to be an expert [of the algorithm] and has to understand what they're talking about. So, algorithms, yes. Algorithms with a lack of understanding, even by the manufacturer [where] nobody knows what they're really doing and the basis of their decision-making, I think is really problematic. ... I think that would need a whole set of legal safeguards around it that is different from the legal safeguards that already exist around expert evidence. So, I'm not saying it could never be done, but I'm saying that I think legal scholars need to think very carefully about the safeguards that would need to be in place for machine learning algorithms to be accepted on their own, without explanation as evidence. ... [In those types of algorithms,] I think it'd be quite difficult to make sure that the validation was sufficiently comprehensive [such] that you never questioned the output because that's what it would be coming to, you don't question the

output because there's no one who can answer the question. So, the extent of validation that you would need to do that you would never need to question the output would be phenomenal, I suspect (O#1).

I certainly think it's possible to use algorithms for court purposes. ... It all depends upon the success of the algorithm and whether it's been validated and is appropriate. I think algorithms may well be preferable to human examiners giving opinions based upon experience because the use of the algorithm reduces the chances for bias and it may allow better estimation and calibration of that strength of the evidence. ... [However,] these models tend to be very complicated and difficult to assess. Algorithms have advantages, but it's going to require a whole new realm of expertise to evaluate them. ... One area where I'm a little bit worried is [whether] the practitioners have enough expertise to be able to assess whether it's working properly in a given instance, in other words, case specific evaluation of the appropriateness of the algorithm. I think it's important to have somebody knowledgeable look at it and say, you know, is the machine in doing this analysis making assumptions that seem plausible in light of what we know about the data? Or is the machine going off the rails? ... Has it done something inappropriate? ... There is a risk that practitioners will use the models without fully understanding them… (O#2).

It depends on what the algorithms are applied to. ... The greatest benefit [of algorithms] is actually to let humans do what they do best. ... The way that I think of the use of algorithms in forensic science is [that] there are things that I think can be done more efficiently by algorithms, and then that frees up the human expertise to deal with the more difficult things. That's the kind of deployment that I'd like to see happen. So, that's the greatest benefit because that gives the people who have the expertise more time. ... To me, the application of algorithmic techniques that are not tied to demographic factors, I'm in favor of that. DNA is an example of that. [However,] algorithms that are tied to large characterizations of populations—that's where I think it becomes dangerous. ... I'm terribly worried [about that], and we already see this. There are algorithms that police departments use, predictive algorithms, about crime, for example, and there have been a number of studies that show that, as one might expect, African-American communities don't fare well for these algorithms. ... So, the biggest danger [is] that [people] will use algorithms inappropriately where bias can come into their views without even knowing (O#3).

Participants' responses related to how algorithms can be trusted for use in court, including issues concerning the disclosure of source code:

<u>Laboratory Managers</u>

That goes to validation and I'm a super big believer in validation. Everything we do should be tested [using] mock samples within the range and scope of anything you're actually going to apply it to, ... [and validation] should be well within the understanding of the expert to be able to answer those questions and [explain] that it's fit for purpose before we used it. I understand the concerns [of trust], but that just means we've got to

do our job in showing these tools are valid before we actually apply them to the case. ... I do believe that having appropriate validation data and showing that you don't have to see in the black box to see that it's reliable. ... I think largely revealing source codes is just a tactic. Nobody's going to take that source code and go "Aha!" ... I'm not going to say it's useless, but to spend the kind of money to make it of any use, in practical terms [it's] virtually never going to happen. That side [requesting source-code] is just going to ask for something, to eventually get the answer of "no," so, they in turn, they have something go "well, if only we had that thing, then we would have been able to show something." ... That said, even though I respect this strategy, if we're being a hundred percent transparent, [if] you want the thing, knock yourself out, here [it is]. ... But I do respect that that is competitive [so] bind it to that particular case only and throw on some significant penalties if it's leaked out, because that is the person's livelihood. ... Frankly, as I said, it's a tactic, and if you give it to them, it's not going to be of much use. ... It's a waste of time, but you know what, knock yourself out, here it is as long as it's protected. ... [Ultimately,] my goal is to maximize the value of evidence. ... If somebody isn't willing to turn [the source code] over and there's a percentage of the time that [the evidence] is compromised in court, I'm going to have to [take that into consideration] ... I'm duty bound to pick the one that's a better product, [and source code disclosure] is a feature. ... I don't have to agree with it or disagree with it. We know what happens, so I'm going to have to choose what's best for our cases, which is the one that gives more value, which is going to be the one that is okay with [disclosure]. I think [the vendors] just have to get past those legal hurdles and [realize] it's just part of the reality of the environment in which they're doing business (LM#1).

[The issue of trust comes down to] how you set up your framework as part of validation and before you start assessing an actual case of what valid data means and looks like, and essentially, how you put into the datasets, the traceable control. What does control mean? ... You need to think through what is basically both that positive and negative control that can be put in as an internal standard within the data collected as part of validation. But then not only is this part of [initial] validation, but it is routinely inserted into all cases so there is an internal standard in everything you do. These algorithms [can] change over time, they [can] learn, they are not [necessarily] static. ... What that internal standard is for a latent print, I don't know, but there needs to be something that's in there that is an assessment of [whether] the algorithm is behaving as I expect on that particular application. ... [That said,] by and large the source code is specious—it's a red herring. These pieces of software are fantastically complex. ... Who else on the planet is going to be able to actually assess looking at the raw source code what anything means there? It's nonsensical. What is sensical is the internal validation of that result. That's the part that should be there. ... It's the standard that is put into those data so that every case, every instance can be self-validated (LM#2).

I think the more open the models and peer discussions are about how these things are done [will improve trust] ... so [that] you're actually able to do peer review and testing, and people could talk about the limitations and benefits. As long as that's done, I think we can advance a lot quicker and to everybody's benefit. But when it's done in a proprietary fashion ... you [have to] feed a lot of unknown "black" data into the system

and you get a result at the end. I can look at the results and see if it's good or bad, ... but I can't get a fully good understanding of what's under the hood. ... There's intellectual property, I get that, but the more open we are, the better [understanding] we're going to have about limitations. ... The problem with validation is I don't have a perfect world [and] validation is subject to some limitations based on what I fed it. ... In a perfect world, what I would like is for the analyst to know the algorithms that are used so manually I could pick apart an algorithm. ... All that guesswork is gone. So now a computer can do all the grunt work for me, and I can actually do it manually and say, this is why I'm attaching a number. That's what I would like to see in a perfect world. It doesn't mean the validations are not important. They are, but they are only black box validations. I don't know what's in the box. ... [That said,] I'm a big proponent of intellectual property, but that's not necessarily for courtroom use. ... [In] the perfect world, if you're dealing with people's lives in the courtroom, knowing everything about how decisions are made is a better approach. So, where do we go from there? We, in [our jurisdiction], have chosen, in DNA mixture interpretations, we've chosen software where they disclosed their algorithms, and we did that for a reason. I think the best way to do this is under protective order, so for purposes of that case, you disclose it, but it's not open to the general public. ... [However,] whether it's absolutely necessary [to disclose source code], I'm not at the point right now that I would say it is. I think you can validate [the algorithm] copiously to the point where you can get reasonably good inferences about its efficacy. It's limited and it's not perfect, but I think it's still usable (LM#3).

<u>Prosecutors</u>

[Trust is] a valid concern and maybe another reason to just not go there unless we need it, which I don't think we do, at least based on what I've seen so far. ... I don't think [source code] is something that necessarily should have to be disclosed in the first instance. However, if the defense wants it, then I think that they should have access to it. I think that steps can be taken to protect any proprietary interests in the source code. ... I think that when you create software that is going to be used as part of the criminal justice system you have to realize that constitutional rights are going to come into play. But I also don't think it's something that automatically has to be disclosed, at least under the rules in my jurisdiction, and I'm guessing most. But if requested and if a good cause is shown, then it's something that could be disclosed (P#1).

[Trust] just goes back to whether there's a sufficient body of data that supports whatever the conclusion is. That's really my only question. Again, if it's scientifically valid and the scientific community is saying this is good science, then as a prosecutor, I'm behind it. Honestly, my opinion is who am I as a prosecutor to stand in the way of scientists saying this is legitimate science, you know, and we agree by and large that this is what should be offered. To me, what drives my decisions here is what is legitimate science and what are the scientists saying? Not as much of what are the lawyers saying about it? What are the scientists saying about it (P#2)?

We have discovery and defense experts in cross examination to settle those questions and explore those questions, and I'm all in favor of giving the defense every tool that they need to investigate the algorithm. ... I trust it because I understand the process of validation. I understand developmental validation and I understand the validation that the lab does to test the limits of the software or the technology in their lab. ... So, if my lab has a great deal of confidence in it, then I have a great deal of confidence in it (P#3).

<u>Defense Attorneys</u>

Transparency is number one. ... The source code has to be turned over to an independent software engineer for the defense to examine and to test on the evidence at issue, you have to have full access. ... There's no counterbalance at all. Trade secrets is absurd. It's absurd that we're even having this conversation as relates to criminal justice period, full stop. ... There is no other, including commercial litigation, where [source-code] wouldn't be turned over and examined. It's good enough for Apple versus Sony and it's not good against the people v. Smith? That concern will never go away. ... They should not get in the business if they don't want to turn it over. This is not Kmart (D#1).

Trust is hard, but really robust validation is a part of that; however, it's not the full answer because what we know is that, for instance in DNA, there are so many variables that can affect the reliability of an outcome that we never test for all of them. We never know the combination of those that might, at some point, affect reliability. Validation can be a big part of [trust], but validation is never without holes. ... We [also] know there've been studies that show that criminal courts are very bad gatekeepers of forensic evidence in a way that they're not in civil court, that they failed time and time again to assess forensic evidence in criminal cases with the same kind of eye that they do in civil cases. [Further,] we know that in civil cases judges have never really disallowed one side in to get access to source code ... and trade secrets is a non-issue because usually they get it in the context of a protective order. It's never been questioned [in civil litigation], but for many years it has been questioned in the criminal justice system because of all the biases that are part of defending somebody who's charged with a violent and maybe vile act. Judges treat criminal cases differently than civil cases, [there's] just really no doubt about that, and because of that, defendants in criminal cases for many, many years have been denied access to source code. Although more recently that trend has turned. ... What would I need to be comfortable with widespread use and acceptance of an algorithm in the criminal justice system? First, I would need source code. ... Developers should not work in any forensic space where the results of their algorithm operation are intended as evidence unless they are willing to publicly disclose their code. ... Second, I would need some kind of oversight board—a team of neutral academic experts—provided with the time and resources to analyze the code, stress test it, and publish understandable reports about the assumptions underlying the code, the limits of operation based on stress testing, recommendations for improvement, and recommendations for testimony caveats based on their work. I wouldn't accept that work with open arms from either the developer or from the forensic science community in general. ... I think the forensic science community has proven time and time again that they are incapable of describing the caveats that should accompany forensic opinions.

Third, a pilot period of years, during which a limited deployment in casework is constantly reviewed by the neutral academic team to make sure that the system is being used as intended and that experts do not misstate the value of the evidence in court (D#2).

The most important thing is transparency. The algorithms and the software have to be made available and they have to be able to be used by experts from both sides. They should be tested to see what the limits are, to push them to their limits to see when they fail. I think that's the whole premise of testing algorithms—you try to make them fail. That's the whole premise behind you doing any kind of validation study at all. So I just think availability [and] transparency is probably the most important issue. ... [Further,] I think giving access to the source code into the software to experts who are working for both sides is important, and giving them the time they need [to have] the ability to see when the software works and when it doesn't work. That is really, really important. ... If prosecutors are going to offer this service, then they should be prepared to turn over the discovery, and the discovery that I'm talking about in this context is the access to source code and the software, as well as all validation information and et cetera. ... Source codes are turned over every day in civil litigation with protective orders. ... The measures that criminal judges have taken to prevent defense attorneys from getting access to the source code are not seen anywhere else. There is no reason why defense experts can't have decent access to source codes. The trade secrets argument doesn't fly (D#3).

<u>Judges</u>

I think [source code] absolutely should be disclosed in every case. I don't see how you can tell the judge, let alone the defense lawyer, [they] can evaluate whether it's a good algorithmic approach or not if you don't know how what went into the source code and what its components were, how they were arrived at it, and so forth. And, give me a break about trades secrets. I appreciate that companies like to make money, but we're talking about human liberty here, and that has to trump any concerns over trade secrets (J#1).

I personally think that it should be open source codes, period. ... I respect the fact that there's intellectual property issues and so forth that's around that, but I think that we have mechanisms to assist in protecting that (J#2).

I think that what it means to be trustworthy is very close to what it means to be reliable, but I think it incorporates something else. Reliability is simply, "does the tool work as it is intended to work?" And it's almost like, "does it calculate in the correct way," is it reliable in that way. Trustworthy certainly incorporates that, but it [also] incorporates something else, which is a concept of fairness, and that has got a subjective component [and] sort of normative component as well. What I would say is that we have got to determine first, what is our standard for that form of fairness that we're aiming towards? ... A trustworthy [algorithmic] tool would achieve both a reliability in terms of functioning as the tool is intended to function, so it has, for instance, an output score [or] an outcome that is expected, but also achieves a level of fairness that I think is quite a

complex question, but it's both of those things. ... I think that source code is important because it goes not only to understanding reliability, but you can tell reliability with output, but source code tells you something else about the selection of the inputs and the weighting. ... So, to sort of reduce the importance of source code down to a memorable phrase, I would say "the means to the end matter." The source code matters because in the criminal justice area, we are in a unique area in the American system where we have through our Constitution set out a framework for liberties, where there is due process and due process on an individual level. When we're dealing with due process and equal protection under the United States constitution, we are now in a world where "the means to the end" matter, the means are contained within the source code. ... In my view, if an algorithm is going to be used for a liberty-based decision, a criminal defendant is entitled to have access to the source code, and I would say for an adequate defense, just as a criminal defendant is entitled to the experts that he or she can demonstrate are needed to put on an adequate defense, that same individual is entitled to an expert who can then help them analyze the algorithm. ... [On the issue of trade secrets,] the one thing that courts know how to deal with are trade secrets, because it is frequently the case that there is information disclosed every single day in courts all over this country that is top secret and is under protective orders, even a highly confidential, or attorney's eyes only, kind of a restriction on the protective order. ... The reality is that there is not a reason for a court to deny access to source code based upon competitive issues. That's not what these defendants are interested in doing. They're not going to go running out and open a competing business (J#3).

<u>Other (Academic Scholars)</u>

I think that we really need to be as transparent as we can be for legal purposes, and this is where I do continue to separate machine learning algorithms from straight programmed algorithms. On the programmed algorithm side, we need to make sure that we are validating them in a careful, risk-based manner and understanding the risk points of the processes, the weak elements, [and] the combinations of circumstances that would make their use more problematic. [Further,] we need to be taking it as a whole system—not just the algorithm, but the data that we're feeding it, the person who is making decisions, ... [and] the person who's explaining it at the other end. So, rather than just concentrating on the algorithm, I think that validation needs to concentrate on the end-to-end system and really understand those weak points. ... For the machine learning algorithms, I think that there really has to be some explicit thinking about where the safeguards are going to be and how and when those machine learning type algorithms would be admissible before anyone tries to put them through as evidence. ... [As for source code disclosure,] I don't think there's a real place for secret science in the criminal justice system. I don't think the basis of an algorithm should be kept secret. I think that the models that [the algorithm] uses and how it works should be in the public domain. ... However, I just feel that the circumstances in which [source code] would be required are pretty rare. ... [Now,] if it happened that in a particular case, the functioning of the algorithm was so central to the actual issue of relevance in the case that it required disclosure of the source code, then yes, disclose it. ... [The problem is] if you were to disclose [the source code]. Who's going to make sense of it (O#1)?

There has to be empirical studies with known source samples that are carefully chosen to match the kind of samples that are processed in casework. And then the proof is in the pudding, you have to see how the method performs under circumstances where you know what the right answer is. You have to do that to assure that it works well for the typical case, and then you have to continue doing it to explore limiting conditions [and] to push the system until it breaks. You need to know the breaking point. Your worry is people are going to be over-confident or there's a problem or error in those borderline cases, and you can get into trouble working with borderline or inappropriate cases if you don't know where the borderline is. ... [As for source code disclosure,] I think framing it as a balance of countervailing risks or issues is the right way to put it. ... The benefit is that the parties, by reviewing the code, might find some issues or some problems that otherwise wouldn't be found if they weren't exposed to outside critical scrutiny. ... Review of the source code by defense experts has in fact uncovered several instances of problems. Although specific examples haven't necessarily been terribly consequential, at least there's the claim that "reviewing of source code is utterly useless," which I've heard people make, has been disproved. ... On the other side, the risk of course is that the source code is intellectual property, and people who have invested thousands or millions of dollars into developing it don't want it to be stolen. ... Has intellectual property theft occurred as a result of defense disclosure? As far as I know, there's like zero, none. ... So, from my point of view, if you balance it, should courts be allowing this disclosure? Yeah, I think it should be done (O#2).

Well, two things: transparency and performance testing. Transparency, because if I were in some sort of legal situation where an algorithm played a role in determining my freedom or even more consequentially my life, I would want my attorney(s) to have the ability to bring their experts to look at the algorithm [and] to make sure that I wasn't a victim of bias. So, transparency is for me, the first thing. The second thing is [that] I want these algorithms tested on a regular basis, looking for failure modes. I want the reliability testing as part of the use of it. ... [As for source code disclosure,] I'm very much aware of the issue of proprietary software and I also pay deference to it. So, if source code is exposed to examination, it should be done so under the conditions that those who are doing the examination are legally, with severe penalties, required not to disseminate that information. In other words, you want to build a fence around your experts so that if they disclose outside of the boundary case, they pay a heavy penalty, which can mean jail, as far as I'm concerned. If you can't get that kind of trusted system in place, then you fall back on reliability testing. Again, you should have a right to test the reliability of the codes as they produce probative evidence (O#3).

Participants' responses related to the use of algorithms based on AI/ML methods:

<u>Laboratory Managers</u>

I can test the black box and show it's fit for purpose. ... Here's my acceptance criteria. I do my testing. It meets the criteria. It works. It's fit for purpose. Now that I determine it's fit for purpose, the better you can make that, if it's self-learning, it [has a] competitive

advantage. So now I've got these two [options], this one is static, and [the other] one is self-learning. The fact that this one can get better [distinguishes it] from the other one. I'm going to choose the better [option]. ... So, you can't turn over source code, [well] I didn't really see that as being a real problem before. ... If it provides a better value of results, which I should show through my validation, my ongoing testing, I should always be picking the one that's better (LM#1).

I think that it is appropriate to use them. You need to have appropriate both positive and negative controlling that are inherent in the data set every time, to demonstrate every time that the result has come out as appropriate. ... [Not knowing the full limits of a black box system] is a concern, and part of validation needs to press as hard as you practically can at where the limits are. I am a big fan of saying test to the point of failure. How do you really know where you need to back off if you can't find a point where it breaks? So, provided you worked hard to try and make the stuff fail [during] validation, [then,] yes, my concern is mitigated on the continuing control and setting what that internal standard is, where you try and bracket your expected results. The perfect circumstance would be, I've got controls that bracket my expected result line so I can demonstrate either side and really everything is an interpolation between those controls, not an extrapolation beyond the limits of those controls (LM#2).

I don't have a problem using them, but again, I think the uncertainties and the fuzziness needs to be fully understood. I think the people that are advocating for using it need to be the first people that talk about the limitations of the methodology. ... A lot of this will come out in validation studies. ... I don't think using it is a bad thing, as long as you know the limitations. If we don't know those limitations, taking it to court then could cause more damage than good, and that's a problem. Those limitations have to be understood before it's actually used (LM#3).

<u>Prosecutors</u>

Who am I going to call as a witness at a [admissibility] hearing to explain how this system works that I'm trying to show meets the admissibility standard for my jurisdiction? … I'm quite certain that maybe comparable types of evidence are admitted in certain types of civil litigation, so there must be a way to do it—they have the same rules of evidence that we do, we just have additional constitutional limitations … [Overall,] I wonder if they could be admitted under current Legal rules regarding evidence and admissibility and then constitutional requirements such as due process and confrontation (P#1).

I would want to see the same type of data to ensure that there's reliability behind any type of machine learning as I would with any type of evidence that is being offered. I have to be able to take my own test drive and understand what it is before I would offer anything. … Prosecutors just can't blindly offer evidence. We have to know that it's reliable. So, whether it's a source code or something behind the source code, I still need to be comfortable. ... [However,] I think that part of what goes into training the algorithm [is where] the science is. Once it's been appropriately trained and then the data supports the

accuracy of it doing what it's called to do, then I have less pause for using a machine learning type algorithm. ... I think it all depends on if it's good information that's going into the software, then I'm hoping that there's going to be good information coming out of it (P#2).

I would think that you would test that kind of algorithm the same way you do any other technology by using known samples. I know what the findings are, and what kind of answer does the machine give me, and that's how you validate it. So, if it were properly validated, I don't see what the problem is. ... I can see the confrontation issue. I don't see a due process issue, but I can see the argument that would be made. Except I think I might have problems with the concept of something that is completely a black box, because you could put the developer on the witness stand to explain how they came up with it, what goes into it, what the considerations are, what the factors are, what the settings are, what the parameters are. So, maybe I'm having problem with the concept of something that's so completely a black box that nobody understands and nobody can explain, because if it's truly that opaque, then I don't see how I don't see how it's useful. I have trouble with the idea that something is so completely opaque that there's no explanation at all (P#3).

<u>Defense Attorneys</u>

No better to have a machine in there speculating. Probably worse than it is a human being. ... You can't have somebody who just turns on the machine and you're coming in and testifying. If we don't know exactly how the machine works, why it works, what its error rates are, how it was developed and why, then it should never be used in criminal court. ... It is, in my view, a sixth amendment violation, no matter what—if you were denied your right to confrontation, you were denied due process of law (D#1).

I think that the understanding of machine learning, even for the most highly trained computer scientists, is really [limited]—there's still a lot for us to know. So, implementing machine learning in forensic science is a scary prospect when it's still not that well understood in the broader scientific community. ... It's a tough question [if I would be comfortable at all]. It might in part go back to my answer about some independent board who not only assesses the algorithm itself, but who has a say so in the training data and how that system is trained so that the decisions made on training data makes sense. I'd be even more concerned about AI system unless we had some of those procedures in place to assess whether these algorithms are being developed and validated in a scientifically defensible way (D#2).

I'm not going to say that it should never be used in a criminal context, but it just seems to me, again, that's a bigger argument for more transparency and for a greater testing by people who are independent so at least when these things come out, they come out well before they're ever used in a criminal case so this is all known and it has all played out long before anybody tries to admit this as evidence against someone. ... I think [admissibility] would have to be on a case-by-case basis. ... I think the complication comes in when we try to find out what's behind the black box. It again speaks to the

importance of giving lots of people access to the algorithm and to the source code. ... I do think there are valid confrontation clause concerns that courts are really going to have to grapple with. But that's true regardless of AI. That's true when you're talking about just any kind of algorithm even if there is no artificial intelligence involved. ... For example, if the forensic examiner is testifying to [algorithmic] results in court and we ask them questions about how the machine is doing X, Y, and Z and how it's deciding to do this versus that, they can't answer that question. We can't ask the machine those questions so there is a level of hearsay and a confrontation issue. So, I do think there are valid confrontation clause issues that really haven't been litigated as robustly as they need to be (D#3).

<u>Judges</u>

At a minimum you need to know what the error rate is. ... But, also, I'm a little suspicious about any notion in the legal system where we say, "we don't know why X causes Y, but we know it does." You know, if you were in a toxic tort case and you said, "this drug causes cancer, but we can't tell you why. It doesn't fit any of our plausible knowledge about how the human body operates. We just know it does." I think a lot of scientists, a lot of lawyers, would be very skeptical about the use of that because ultimately the law depends on reason, not on assumptions. ... So, I am skeptical of the black box approach (J#1).

They fascinate me and scare me all at the same time. I can't say that access to the source code is the "be all and end all" of anything. I do think that there are some black boxes that we may not know, but I don't even know how to begin to assess that stuff. I think it's got all sorts of potential, potentially good applications, but it's [a] pretty open question. It's kind of scary. ... [Whether the use of these algorithms could be an infringement on Constitutional Rights, such as Due Process or Confrontation,] I think that's part of where it has [been] raised thus far in the litigation that's begun around the country—mostly with the component of open discovery laws and confrontation. I don't think that in the end, it's going to be an absolute barrier. There's lots and lots of courts around the country that have already approved the use of a number of different black box type models in DNA without requiring source code and without requiring things beyond sort of validation studies and so forth, either by the lab or by the industry that created it in the first place. ... But, I really think that if we're going to start using them, that we need to figure out what it is that we do need for purposes of making sure that there's essentially buy-in from everybody, that this is why this is working and that we can have some check on the fact that it is working in the way that we believe that it's working (J#2).

It is true that the source code can be a black box, and the source code can be perhaps incomprehensible to mere mortals. ... What are we going to see if this stuff is nearly incomprehensible? Well, first of all, there is a design information that's behind the source code. You can find a variety of instructions that are actually in the source code, and you can also talk to the designers of the source code. The "source code" we use is a shorthand—it can also be access to the design of the instrument. So, let's not limit ourselves just to getting a drive with a source code [file]. Let's think about it as access to

the design of the instrument. ... Understanding how the instrument was designed is absolutely critical to understanding the calibration of the instrument and the choices. ... They all had a human progenitor at one point in time who designed the objective of the tool, who designed the initial manner in which the tool was going to work. ... So, there's a whole bunch that goes into a source code. What are we going to learn? We don't know until we look. Are we going to reach a point where the source code is no longer informative? Maybe, but I would suggest to you that either we're not there yet, or we don't have to be there yet. There are ways in which we can ask the tool itself to give us information on what it's using as inputs, what it's using as its weightings. We can review those and determine whether they correspond to our sense of fairness. ... If you were in Europe and you were under a GDPR framework, you would be required to make the logic of the output understandable to mere mortals. It is doable. So, I don't believe, and I'm not ready to accept right now that the black box of source code means that we back away from it and say, "ah, it's too complex," because then we have given up extraordinarily important constitutional principles to this black box algorithm. We can't do that. We don't have the right to do that. There is no principle under the American justice system that allows us to do that. ... [Ultimately,] I think there are serious due process issues with a defendant being denied access to understanding information that underlies a tool being used for liberty decision. ... We should not just assume away the importance and the benefits of cross examination [of the algorithm through the expert] because of the complexity of the tool. ... [If the expert is unable to adequately describe the details underlying the tool,] I think at that point in time, the defense counsel could argue that there should be an exclusion of the evidence, without access to that tool, because they're unable to explain what's underneath it, and so we have no idea, we're unable to test it. These tools are unregulated right now. ... What we're doing is we are making assumptions based upon an unregulated set of design criteria that the tool has been made in the right way. I say, it's too important a decision to either leave it completely unregulated or not allow at least examination into the underpinnings of the tool (J#3).

<u>Other (Academic Scholars)</u>

I think if [the algorithm] is not understood to the developers and it's a total black box, then I struggle to see on what basis that there is fair transparency in the [legal] proceeding. ... You can validate black box, [but] you're going to be limited because, to me, validation is based on risk and risk is based on understanding. If you don't know where the weak points are, it's very difficult to do a validation that is sufficiently comprehensive, that it will pick out all the weak points when you've had nothing to inform that. ... You could validate forever and not get to the end of the set of circumstances [for which the algorithm could be applied]. So, how do you find the weaknesses (O#1)?

That's a little scary ... for those [types of algorithms], maybe we just need to rely more on the validation on known source samples. ... I think we should take full advantage of the AI and other approaches to improve our accuracy, but it's particularly important in those cases that if we don't actually understand how it's doing what it's doing, then we may not fully understand how it could break down and where the limits are. So, I would say for

those kinds of algorithms, it's even more important to have testing that explores the limitations and where they break down (O#2).

I am not supportive at all in that case, because with modern machine learning, even the people who develop the AI don't know what it is doing. So, in those circumstances, reliability testing is the only thing I know that you can bring to bear. ... [Although] they'll be black boxes, ... you can give them these large data sets and you can watch how they perform so that you can quantify [and] measure how inputs and outputs are related. ... Theoretically, it could be acceptable to use these systems if we have reliability testing. [The problem is], the testing has to be large and broad because you don't know where the failure modes are and therefore have to do the equivalent of stress testing—you apply the most severe scenarios that you can to test the reliability since, in the case of ML, that's the best you can do. ... [That said,] is this type of reliability testing practical? What I've talked about is the ideal. I don't think the idea is actually practical [and] realizable. I don't think you could actually implement it (O#3).

Participants' responses related to whether algorithms should be regulated and, if so, by whom and how:

<u>Laboratory Managers</u>

I feel that a weakness of our forensic science enterprise is that we don't have a cohesive, guidance mechanism as much as I think maybe we should. I do respect that people don't want to have big brother saying that this is the only way to do it. Yet at the same time, there's danger to our disciplines if we fragment. ... Now that is evolving. We've got OSACs and we're moving in a better direction, but there really is no office of forensic science. There is no central coordination. People can still disregard right now. ... [There is also] a tremendous amount of duplication of effort [across the enterprise]. ... I think [full regulation] would probably be considered by many as an overreach, but the court system in a way should be self-regulating to a point. ... I think it's been fairly reasonable so far and I think the defense community is pretty well interconnected that when [issues] come out, they're on top of it and that information diffuses. So, I think there is some fairly successful self-policing. I just would like to see a little better organization and cohesion to do that (LM#1).

I'm not sure I've got a good answer for that. ... It's not a broad practice, but there's at least the concept of licensing analysts [and] accrediting laboratories. What is really different about a piece of software? ... Why wouldn't you license a piece of software? It gives a framework for some audit and accountability. It's certainly going to increase the cost of everything. ... [But] having some kind of framework of audit and accountability probably has some merit. Who? That was a tough one to say too. The logic would be something that is more [on] the national level, [such as] NIST or [an entity] like that. However, you're dealing with adjudication of these laws being a state's right. How does that quite work? You basically have to have a nationally licensed tool for something that is a state's right. I could see an awful lot of push back. ... I'd love to think [that an oversight regulatory body] was an advantage, but I've seen a lot of places where it gets

to be a hindrance really quick. ... [As opposed to a regulatory oversight body that approves specific algorithms or algorithmic tools], maybe it's more about the structure of the requirement for the system of internal standards and controls and the demonstration time over time over time, every case, is really more of the way of doing it (LM#2).

I do think they should be standardized. Regulated, I don't know. I don't know if I have an answer to that. I'm a proponent of ultimate standardization and the industry deciding what's best practice. After that's done, if regulation would help implement, fine, but I've seen too many things have been regulated that shouldn't have been regulated. So, I would rather that the industry itself develop best practice like we're doing with OSAC and like we do in academics before the government actually steps in. Every court case is different. I think the attorneys and the judges should be able to have the flexibility to use the information appropriately (LM#3).

Prosecutors

Certainly, it should not be regulated in any way by the legal side, the legal system. I think that would be a question that would fall within the relevant scientific community. ... I don't think it should be the lawyers at all. I think what you're going to do and how you're going to do it is something that falls on the scientists because you all are the scientists. And then, if we can use it and how we can use it, that falls on us (P#1).

I think [algorithms can be regulated] in the same way that forensic science is already being regulated. It's being regulated through best practice committees and through the court system, and I think that those are putting sufficient limitations around forensic science in general, and that would apply the same with algorithms (P#2).

I think that regulation in a reasonable way gives everybody confidence in the science. ... [However,] I'm not sure what that regulation would look like, and I'm not sure how, for lack of a better word, political, as opposed to scientific, that regulation would be. I've seen things become political very fast, and so I don't know how you stop that from happening. If the regulatory group or body becomes political then it becomes useless as far as I'm concerned. ... I feel like they get hijacked by non-scientists who have a very definite agenda. That does not work very well because the science question has been left way behind and the argument is all about something else (P#3).

Defense Attorneys

Yes [algorithms should be regulated, and] I think it should be an independent scientific entity or something like the Food and Drug Administration, or it should be housed in NIST [(National Institute of Standards and Technology)], a scientific body that's a measurement-based science. That takes the pressure off of particular practitioners or particular cases or prosecutors. [This way] you can understand the limitations of a forensic technique and [ensure it is] validated outside of criminal court. We do that for every other consumer product, but we don't do it forensics (D#1).

There should be independent bodies to assess their function, their validation, how they operate, who should be able to review training data, who should be able to require the appropriate caveats during testimony, who should be able to require that proper standards are used to develop [the algorithms], whether it's IEEE standards or others. ... [The notion that the legal system could regulate algorithms is] really a laughable position. The criminal justice system has proven to be an utter failure as gatekeepers of forensic evidence. We've opened the door to bite mark evidence that wasn't validated. We've opened the door to bullet lead analysis that didn't make sense. We allowed decades worth of misstatements of hair comparison, evidence that overstated the value of it. Fingerprint evidence had overstated the value of it. DNA mixture interpretation that overstated the value of it. And never, even when presented with the other side that raised questions about that, almost never did the criminal justice system have the capacity to properly assess that. That capacity will be even less when it comes to computer-based systems. Judges will continue as they have with human-based systems to utterly fail as meaningful gatekeepers of forensic evidence (D#2).

Yes, and by IEEE or something along those lines. It seems like there should be a minimum [set of] requirements. ... I think it has to be an approval authority [and] they also have to regulate how the algorithms are used (D#3).

<u>Judges</u>

Yes, [but] not just algorithms. I think there is a real need for an Institute of Forensic Science staffed by a high-level scientists who could tell us with the neutrality that we deserve, this is good forensic science, this is bad forensic science, this is possible forensic science but it has to be improved and here's how to go about improving it. That was essentially the recommendation of the 2009 National Academy of Sciences report, and I'm very disappointed that it's never developed much traction. ... [While some stakeholders might think the legal system is an appropriate means of regulating forensic science], I think it has proven to be defective. [With] all these cases where there was forensic science introduced and then the guy turned out to be innocent, I don't think that can be brushed off. These are human beings who are being sent to prison, often for very long terms, and [it's] not just one or two, although that would be bad enough, but hundreds because of defective forensic science. Now, can a legal system make that less likely? I think Daubert was a step in the right direction. I'm very disappointed that it hasn't worked in the criminal context the way it has worked more successfully in the civil context, but I don't think the legal system, ultimately, is well positioned to regulate forensic science. Judges know beans about science. Lawyers know beans about science. The natural thing when you have that kind of problem is to turn it over to the people who do know about science, the scientists. So, I think that would be a better approach (J#1).

Yes, [but] the by whom and how is a much harder question. ... [Whether the legal system is an appropriate means of regulating forensic science,] no, [but] I will also say I'm not sure the federal government is the place to regulate it either (J#2).

In my view, there should be a form of regulation that is for any liberty-based decision. It's a broad question in terms of algorithms and any kind of forensic science, ... [but] if it's going to be used for a liberty-based decision for a human being, then they need to meet the constitutional standards, so they should be regulated. But, they need to be regulated in a very careful way, by people who are in the field and who are responsible for upholding the constitutional standards in the criminal justice area. ... I think there does need to be some form of regulation. The, how, I think, is extraordinarily complicated, but I don't accept that it can't be done (J#3).

<u>Other (Academic Scholars)</u>

It's not the algorithms that need to be regulated, it's the methods, and the methods include the people, the algorithms, the data, and everything else. ... I think that if we regulate an algorithm, we're not regulating inappropriate use of the algorithm. So, we're much better [off by] regulating the method ... because [that] enables us to make sure that people aren't just putting any old [junk] in and that they validated it, that they are trained and competent people that are able to interpret it, [that they] are able to explain in court the conceptual basis of it, and so on. That's what I think that needs regulating rather than just that little bit in the middle, [just the] algorithm, because otherwise [junk] in, [junk] out (O#1).

Yes, I still think it would be nice if we had a national institute of forensic sciences contemplated by the NAS report in 2009. I think the OSAC approach to creating standards is beneficial, but I think that the OSAC approach does not do well for, um, assuring rigor of rigorous validation. ... Right now, we're stuck with the regulatory authority being exercised by judges who, for the most part, have not shown a willingness to apply rigorous quality control with regard to validation of forensic science. ... Years ago, when I first started out interested in this field, I thought the path forward was going to be through litigation. I thought that litigation under Daubert and Frye was what was going to establish a quality control for forensic science, and it just needed people like me to come in and explain to these judges why they needed to set rigorous standards. That didn't work. I spent a number of years litigating cases and I ended up feeling I was getting nowhere. I mean, judges are not well-positioned to evaluate science and they're not competent to do it. They really want to see the evidence admitted [and] they don't want to hold up criminal prosecutions because of uncertainties about some nuance of evidence. ... I ended up thinking that litigation is the worst possible way to try to resolve scientific dispute about validation—it polarizes everybody into opposing camps. So, I'd like to see more federal involvement with agencies that have the ability to make some scientific assessment and set regulations on their own. I think that would be appropriate (O#2).

My intuition is, yes, but I don't know how you could do that. I don't know what a regulatory machine looks like, so I don't really feel qualified to answer this question. ... But [if you ask me] as a citizen and potential member of a jury, then I don't want them in the court. ... If the algorithms are based on machine learning that are total black boxes, I don't want them in the court (O#3).

Participants' responses related to what they would describe as the greatest challenges facing the operational use of computational algorithms in forensic science for court purposes:

<u>Laboratory Managers</u>

> *Resources. To stay on top of how quick things are developing, it's taking more and more resources. We all have backlogs and we're focusing on those. To take people off of [casework] to train them, then get these new things up to speed and implement them and then change people's minds [takes resources]. ... How can we do a job in a technological field without the resources to bring in these new things? Not only are algorithms coming, they're already here. It's allowing us to do a better and better job. But it takes resources to do that (LM#1).*
>
> *Resources. Because software itself is expensive, even more so though, is the training and implementation arc of getting people to accept it and understand it, to be able to use it and use it correctly. That's an expensive effort. And let's face it, labs are underwater already. ... Trying to get a group that is underwater, desperately overwhelmed, that can't catch their breath between [cases], to have enough bandwidth to even be able to accept a new tool and not see it as just, "oh my God, you have one more thing." That's going to take time. And, even we don't have bandwidth in there [despite being a relatively well funded laboratory compared to others]. ... That's what's going to face all of these algorithms. ... It's not that people don't see the advantage of them or see the potential benefit, but how do we get from here to there when everybody is madly trying to decide which horrible, awful crime they're going to put first and which horrible, awful crime goes second. So that's what's under that trivial answer of resource. Then, you also think of all the rest of the infrastructure that goes with being able to effectively use these algorithms—the compute, the storage, the data management—where do we put all of these results? How do we store all of these results? How do we maintain that output, which has probably got some proprietary aspect of the outputs in such a fashion that 20 years from now I can still access those results and be able to explain it? Again, it comes back to a resource issue of all of the infrastructure that goes around the use of that algorithm (LM#2).*
>
> *It's the difficulty in actually developing and implementing [the algorithms]. Getting public data [to support the development], because we have privacy issues and so forth, and then the resources that are needed for the practitioners to begin integrating into their day to day (LM#3).*

<u>Prosecutors</u>

> *We want science to evolve, so we're happy to embrace new things as things get better. But then assuming we got past those [admissibility] hurdles, I would just say making it more complicated—taking evidence that right now I don't consider to be that complicated and making it more complicated would probably be the biggest challenge for me. ... It*

would make the presentation of scientific evidence more difficult in trials. ... The more complicated you make that, the harder it gets for the scientists to communicate with the people that they need to communicate with—be it the jury, the attorneys, the cops—to explain what their findings are (P#1).

I think it's getting stakeholders to understand. ... I think [algorithms are] very foreign to people in the entire forensic science community. You're going to get pushback from current forensic scientists [and] you're going to get pushback from all types of lawyers. Judges are not going to understand it. It's just not something that we're used to. So, I think we really want to see the data and understand it. I think that's really the issue, is understanding and ensuring that it's reliable. I just want it to be something that is scientifically valid and clear for our presentation (P#2).

I think training the scientists within the labs, to validate it, and to understand it and have confidence in it. I'm not the scientist. I'm using the science and what I want is reliable science that is easy to understand and easy to explain to lay people. So, if the scientists from my public labs, if they're well-trained, they understand it, and they have the confidence in it—that's the challenge, to make sure that happens because under those circumstances, I think the entire system can have confidence in the output and confidence in the results, even though some parties in the courtroom may not be happy with the results (P#3).

Defense Attorneys

Probably the practitioners themselves. I think that every time [watch presentations] the presenter will get booed off the stage if they don't make it really, really clear that we still need the examiners and they still have to do exactly what they've always done and nobody's losing their jobs. ... It will [also] be money and education, because if you don't have the educational requirements that would be necessary to have people that are engaged in higher level math that it's going to require, then it won't happen. [It will depend on] the amount of money that we're willing to invest in forensic sciences to get them more scientific and also the amount of money that we're willing to invest in the education of our forensic analysts. ... You shouldn't have to fire all the latent fingerprint experts and go to Stanford and hire a bunch of Stanford grads. You have to be willing to invest in the training and get those examiners where they need to be, to understand how to use this machine. ... Status quo is an incredibly powerful force. You could just say that the status quo is going to be what's preventing it. You know, bite mark evidence is still admissible in all 50 states… (D#1).

I think [the greatest challenge is] the people occupying positions of judges without the interest or competency to understand. There's a long-documented history of criminal judges not understanding scientific evidence ... and they don't really have an interest in it. I've been in front of judges who have said on the court record, "it's not really my job to second guess scientists. That's not what I do. I am not qualified to second guess the scientist." Even though that is the very role of a judge, that it is inherent in the job, in the role of a judge, to do exactly that. ... They will continue to be the biggest hindrance to

the assessment of complex scientific evidence in criminal cases. ... It is hard to imagine a day when this reality improves enough that judges have a positive effect on forensic science rather than the effect they mostly have today, which is maintaining the status quo in favor of police and prosecutors (D#2).

[The greatest challenge] is these non-scientists understanding what this machine is doing and the limitations of what the machine [and] results are. [Further,] having a forensic examiner, very few of which have a background in computational . . . anything, explaining accurately to these lay people what this machine is doing and the limitations of what this machine is doing (D#3).

<u>Judges</u>

N/A

<u>Other (Academic Scholars)</u>

Shared understanding is one of them. ... I think we need to be careful that we're all talking about the same thing and that where there are differences, say between programmed and AI strict machine learning [type algorithms], and even within that category that we recognize differences between supervised and unsupervised learning and those such things. Another challenge is that there needs to be a scientific, and not an emotive, debate about the issues. So, rather than there being a whole series of high profile, court cases where the use of a particular algorithm is at the center of the case, I think we're much better to plan and set requirements ahead of time, and really think about if we're going to use algorithms, this is how we're going to use them—these are the validation standards that you have to meet, this is how you have to make sure that the end-to-end process works and not just the bit in the middle, and here is the legal framework within which they work. ... [Finally,] I think we need to really work on education of practitioners and our legal colleagues in terms of fundamentals of probabilistic [concepts], in terms of what it means to be transparent and to disclose limitations, and how we work with these kinds of new technologies (O#1).

Challenge number one is, can we come up with good algorithms? ... The technical capabilities of modern statistical techniques are impressive and there's tremendous potential to come up with machine-based approaches that have the potential to approve on human judgment. So, first challenge is let's come up with really good, robust algorithms and then evaluate them carefully to show that they work well. The second challenge will be implementing them by forensic science practitioners who don't have the training and background to fully understand how these things work. Most people who become forensic science practitioners are not very sophisticated about statistics and in fact find these things kind of frightening. I think the DNA people have been able to adopt and incorporate the probabilistic genotype, but I think it's been kind of wrenching for them. I think it's been really challenging for some of the analysts to come to grips with it. I see a lot of evidence that, even among practitioners who think they understand these things, maybe they don't understand them as well as they think they do. ... [Additionally,]

doing the training needed to operate these things in an effective way and make them understandable in court is another huge challenge. ... I don't think it's a reason not to develop algorithms. We need to be realistic about how easy it is to implement them. ... I think it's the future. I think it's the path forward. I think it will address and resolve a lot of problems that we're facing with human beings making these judgements so I think there are great prospects and making greater use of algorithms will improve forensic science, but it's not an easy fix. It's going to be challenging. It's going to require a lot of training. I think we need to think seriously about, given our movement toward these algorithms, the way we train forensic scientists and select them. So, picking people who have higher levels of mathematical and statistical aptitude training might be really important. At the same time, I think we need to be sensitive to current practitioners who are math phobic and, kind of ease them in and select more of those practitioners who have degrees in math and statistics, or the harder physical sciences and, thus, may be capable of moving into the new world with a greater degree of facility than we may see from the typical pattern matching person (O#2).

The greatest challenge is not to be caught by the race to the bottom, because if you're going to be doing this through commercial entities, it's going to invariably become the race to the bottom in terms of cutting costs. To me, that's the greatest challenge, is how do you do this without people pursuing pathways that cut corners, and therefore we risk the reliability of the systems. I mean, how do we stop that from happening? To me, that's the greatest challenge (O#3).
